# High-resolution atmospheric-pressure MALDI mass spectrometry imaging workflow for lipidomic analysis of late fetal mouse lungs

**DOI:** 10.1038/s41598-019-39452-3

**Published:** 2019-02-28

**Authors:** Vannuruswamy Garikapati, Srikanth Karnati, Dhaka Ram Bhandari, Eveline Baumgart-Vogt, Bernhard Spengler

**Affiliations:** 1 Institute of Inorganic and Analytical Chemistry, Justus Liebig University Giessen, Giessen, Germany; 2 Institute for Anatomy and Cell Biology II, Division of Medical Cell Biology, Justus Liebig University Giessen, Giessen, Germany; 3 Present Address: Institute for Anatomy and Cell Biology, Julius Maximilians University Würzburg, Würzburg, Germany

## Abstract

Mass spectrometry imaging (MSI) provides label-free, non-targeted molecular and spatial information of the biomolecules within tissue. Lipids play important roles in lung biology, e.g. as surfactant, preventing alveolar collapse during normal and forced respiration. Lipidomic characterization of late fetal mouse lungs at day 19 of gestation (E19) has not been performed yet. In this study we employed high-resolution atmospheric pressure scanning microprobe matrix-assisted laser desorption/ionization MSI for the lipidomic analysis of E19 mouse lungs. Molecular species of different lipid classes were imaged in E19 lung sections at high spatial and mass resolution in positive- and negative-ion mode. Lipid species were characterized based on accurate mass and on-tissue tandem mass spectrometry. In addition, a dedicated sample preparation protocol, homogenous deposition of matrices on tissue surfaces and data processing parameters were optimized for the comparison of signal intensities of lipids between different tissue sections of E19 lungs of wild type and *Pex11β*-knockout mice. Our study provides lipid information of E19 mouse lungs, optimized experimental and data processing strategies for the direct comparison of signal intensities of metabolites (lipids) among the tissue sections from MSI experiments. To best of our knowledge, this is the first MSI and lipidomic study of E19 mouse lungs.

## Introduction

Lipids are versatile biologically active compounds involved in important physiological and pathological processes. Lipids are the structural integral components of the cell membranes, function as signalling molecules in various cellular cascades, and support energy homeostasis, based on a huge diversity of lipid species with unique physico-chemical properties^1,2^. Disruption or imbalance in lipid composition or metabolism lead to various metabolic disorders such as diabetes, obesity, atherosclerosis, or infectious and neurodegenerative diseases^3^.

Lipid composition varies significantly from organ to organ and even differs within the various cells of an organ^4^. For example, the lung is composed of more than 40 different cell types and each cell shows a distinct lipid distribution pattern^5–7^. Furthermore, type-II pneumocytes - also called alveolar epithelial cells type II (AECII) - synthesize and secrete via exocytosis a complex mixture of lipids and proteins into the alveolar space, forming the pulmonary surfactant^8,9^. One of the major functions of the surfactant lipids is to reduce the surface tension at the air-liquid interface, and thereby decrease the tendency of alveolar collapse during end-expiration^9,10^.

In fact, lipids account for up to 20% of dry weight of the lung tissue^4^. These lipids are vital in the lung and participate in many cellular functions. Especially, they (1) neutralize free radicals and reactive oxygen species^11^, (2) protect lungs from inhaled particles and pathogenic micro-organisms^12^, (3) act as a potent antiviral agent^13–15^ and (4) are involved as biologically active secondary lipid messengers in the regulation of lung inflammation^16^. Indeed, abnormalities in pulmonary lipid/surfactant composition and/or metabolism are closely associated with various pulmonary diseases such as respiratory distress syndrome^17^, hyaline membrane disease^18^, asthma^19^, chronic obstructive pulmonary disease^20^, bronchiolitis^21^, cystic fibrosis^22^, pneumonia^23^, interstitial lung diseases^24^, lung injury^25^ and cancer^26^.

Several studies have analyzed the pulmonary lipid composition of different mammalian species in normal and diseased states^4,6^. The most widely used methods for lipid analysis are hyphenated analytical techniques such as liquid chromatography or gas chromatography coupled to mass spectrometry. Matrix-assisted laser desorption/ionization mass spectrometry imaging (MALDI MSI) is a prominent, sensitive analytical tool, which provides molecular information and spatial distribution of the analytes directly from the biological specimens. MSI has received increasing attention, since (1) labelling is not necessary, (2) hundreds of molecules can be detected in a single measurement and (3) spatial distributions of biomolecules in biological tissues are provided^27^. From its introduction in 1994^28^ until to date, this technique has been applied to visualize a broad spectrum of analytes such as lipids^29^, proteins^30^, peptides^31^, metabolites^32^, n-linked glycans^33^, drugs^34^ in various types of samples including mammalian tissues^35^, cells^36^, plant tissues^37^, insects^38^ and microbial cultures^39^.

A large number of studies utilized MSI techniques in lung biology, to investigate the distribution and quantify drugs in different types of lung cancers^40^, in tissues treated with several anti-tuberculosis drugs^41,42^ and to molecularly characterize various lung cancers based on their proteomic profiles^43^. MSI has been applied to visualize the major surfactant lipid dipalmitoylphosphatidylcholine [DPPC, (PC32:0)] in human lung^44^ and in xenografts from human lung cancer cells^45^. Berry and colleagues developed a modified optical cutting temperature compound as a new inflation and embedding material to map the major phospholipid species in adult mice^46^ and in human lungs^7^. Carter and colleagues employed MALDI MSI as a tool for the detection of novel molecular biomarkers in radiation-induced lung injury^47^. In line with these technical developments, three-dimensional (3D) imaging workflows have been developed to visualize phosphatidylcholine (PC) and sphingomyelin (SM) lipid species in entire adult mouse lungs^48^.

It is apparent that characterization of molecular information (lipids, metabolites and proteins, etc.) of the lungs in diseased states, during fetal and postnatal stages, is a key to understand the molecular alterations during lung development and the pathogenesis or pathophysiology of various pulmonary diseases, associated with these indispensable biomolecules^49,50^. We recently reported significant stage-specific differences in the individual lipid-species composition of mouse lung during postnatal pulmonary development processes^49^. However, so far no information is available on the lipidome of fetal mouse lungs, from either MSI or liquid chromatography-mass spectrometry.

In this study, high mass accuracy (≤2 ppm), high-resolution in mass (140,000 @ *m/z* 200) and space (10 µm per pixel) atmospheric pressure scanning microprobe MALDI MSI (AP-SMALDI MSI) was used for the first time to characterize the lipid profile of late fetal mouse lungs at day 19 of gestation (E19) in positive- and negative-ion mode. An optimized sample preparation protocol and data analysis workflow were developed for the reliable and reproducible relative quantification of lipids and other cellular metabolites in different tissue sections. To demonstrate the power of our optimized method for relative comparisons of distinct lung sections with alterations in lipid content, knockout (KO) mice with a peroxisomal biogenesis defect (*Pex11β*−/−) were used in addition to wild type (WT) sections, as an experimental model.

## Results and Discussion

MALDI MSI has become a fundamental qualitative and quantitative analytical tool for the analysis of a large variety of substances in biological specimens. The recent technological improvements in MSI such as incorporation of high-resolution accurate mass spectrometers (e.g. orbital trapping mass analyser), higher lateral resolution under close-to-physiological conditions (subcellular level, 1.4 µm spot size)^51^, molecular and topographical analysis of 3D surfaces^52^ has further advanced the field and its application value. Signal intensities of analytes in MSI depend on several critical parameters, such as sample handling and preparation, tissue thickness, matrix properties (choice of matrix, solvent, application method and local matrix concentration), intercomponent charge competition, spatial resolution, variance in the experimental, instrumental conditions and ion suppression effects^27^. Therefore, to achieve high quality reproducible information from MSI and to compare signal intensities of analytes in a set of tissue sections, a dedicated sample preparation and data analysis strategy within the imaging workflow is mandatory.

In the present study, we established a sample preparation protocol for optimal tissue processing, sectioning and lipid profiling of E19 mouse lungs using high-resolution AP-SMALDI MSI in positive- and negative-ion mode. A detailed data processing framework was optimized to compare the differential expression of cellular metabolites in distinct tissue sections to each other. As an experimental model to eventually show differences in the lipid composition between distinct lung tissue sections at late fetal stage, we analyzed and compared lipids in different lung sections of E19 WT and *Pex11β* KO mice. PEX11 proteins are peroxisomal membrane proteins which consist of three different isoforms (PEX11α, β and γ) in mammals and play an important role in peroxisomal proliferation^53^. *Pex11β* KO animals, which die immediately after birth (neonatal lethality), showed reduced numbers of peroxisomes and severe pathological features of Zellweger syndrome, such as developmental delay, hypotonia, neuronal migration defects and neuronal apoptosis^53,54^. The underlying molecular mechanisms of these pathological alterations in *Pex11β* KO animals are still poorly understood.

### Optimized workflow for late fetal mouse lung lipidome using AP-SMALDI MSI

**Figure 1:**
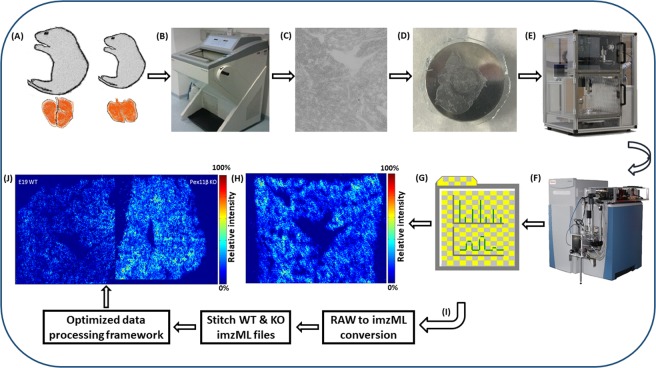
Optimized MSI workflow for characterization and comparative lipidomic analysis of late fetal mouse lung sections at day 19 of gestation (E19) using atmospheric-pressure scanning microprobe matrix-assisted laser desorption/ionization mass spectrometry imaging (AP-SMALDI MSI). (**A**) Breeding of WT and *Pex11β* KO E19 animals^53^. (**B**) Cryosectioning of fresh snap-frozen E19 mouse lung tissues for MSI experiments using a cryomicrotome. (**C**) Optical phase contrast image of a 12 µm thick section of E19 mouse lung and selection of optimal sections. (**D**) Tissue section fixed to the sample probe. (**E**) Homogenous deposition of matrices using an automatic pneumatic ultrafine sprayer (“SMALDIPrep”). (**F**) MSI data acquisition using an “AP-SMALDI10” ion source coupled to a Q Exactive mass spectrometer. (**G**) MSI data sets (.raw files). (**H**) Characterization of E19 (WT) mouse lung lipidome. (**I**) Optimization of the data analysis workflow for comparative lipidomic analysis. (**J**) Comparison of relative signal intensities of lipids between WT and *Pex11β* KO E19 lung tissue sections.

A scheme of an optimized workflow to characterize and compare lipid species directly from tissue sections of different E19 mouse lungs using high-resolution AP-SMALDI MSI is shown in Fig. 1. Briefly, sectioning of E19 mouse lungs, selection for section quality and ideal matching of section thickness, uniform deposition of matrix on tissue surfaces and MSI methodologies were optimized in positive- and negative-ion mode for comprehensive lipid mapping of E19 mouse lungs. E19 WT and *Pex11β* KO mouse lung MSI data sets were acquired individually under identical experimental and instrumental conditions. Thereafter, data sets (.raw) were converted to the common data format for MS imaging (imzML) using the in-house developed “RAW converter” version 1.1.0^55^. The converted WT and *Pex11β* KO imzML files were stitched together using “imzML converter” version 1.3^56^, and a detailed data processing framework was optimized for comparative lipidomic analysis of E19 WT and *Pex11β* KO mouse lungs. The individual steps in the optimized workflow are described in the following.

### Optimization of late fetal mouse lung tissue sectioning procedure for MSI

Sample preparation is the most crucial step in any MSI experiments. Initially, we obtained tissue sections from PFA-fixed OCT-embedded WT and KO E19 mouse lungs (Fig. S1A), which is the most-common tissue preparation method used for pulmonary histological studies. In general, OCT provides a smooth cutting surface and preserves the tissue architecture^57^. However, OCT contains benzalkonium salts as a preservative which suppress lipid ionization and form adducts with endogenous lipid species^46^. Furthermore, OCT contains polyethylene glycols and polyvinyl alcohol polymers, often causing ion suppression and strong background signals which can severely interfere with MS acquisition and data interpretation. OCT and other polymeric embedding materials are thus not compatible with imaging studies of small molecules (e.g. lipids, metabolites). Carter and colleagues recently demonstrated formalin-fixation via tracheal instillation and fixative inflation of the lung followed by gelatin embedding for lipidomic analysis of larger mammalian (monkey) lungs^58^. However, this method showed loss of phosphatidylethanolamine (PE) and reduced signal intensities of phosphatidylserine (PS) lipid species due to chemical crosslinking of primary amine groups^58,59^. Owing of drawbacks, these methods (e.g. formalin/PFA/agarose inflation and/or embedding with polymeric materials, etc.) are not appropriate to map the complete lipidome of lung tissues using MSI. Therefore, cryosectioning of tissue without using any embedding material is more suitable for MSI, which would aid to map a comprehensive lipidome and/or metabolome without any losses or chemical modifications.

The most challenging part in our approach was the preparation of E19 mouse lung cryosections for the lipid imaging studies. In order to detect all possible distinct lipid classes with less and/or no background signals, fresh frozen lung tissue was mounted with water on tissue holders for cryosectioning. 12 µm thick frozen sections were carefully cut and collected on a glass slide without embedding material. However, we frequently failed to obtain WT and KO tissue sections on a single objective slide for measuring both sections in a single experiment, a procedure which diminishes experimental artefacts in comparative analyses. Due to differences in lung tissue consistency of WT and KO sections, we often observed problems of different section quality on the same objective slide, WT sections sometimes containing a fold, or KO sections being broken several times in longitudinal direction (Fig. S1B). We therefore decided to mount WT and KO tissue sections on different glass slides to be able to select them according to their section quality and thickness. Even though always 12 µm were used as cutting thickness, we frequently observed variations in thickness of subsequent cryosections of the same tissue block of WT animals. These variations were even more pronounced due to the differences of tissue consistency between WT and KO animals (Fig. S1C,D). To check the effects of section thickness on MALDI imaging analysis we intentionally measured sections with distinct section thickness of WT animals. Indeed, we observed a drastic change in signal intensities of lipids between tissue (E19 WT) sections of varying thickness (Fig. S1E,F, data not shown). We therefore tried to develop a quick procedure to select for similar thickness of consecutive sections of WT and KO mice on different glass slides using water (ice) mounted tissue blocks (Fig. S1G,H). We optimized a quick photographic imaging procedure and digitally created full section phase-contrast images of all cut sections (see materials and methods). We then carefully compared grey values reflecting the section thickness and selected similar grey value images of WT and KO tissue sections with optimal tissue quality (criteria mentioned in Fig. S1) for preselection of the best region of interest in comparable tissue areas within images by avoiding tissue foldings and other sectioning artefacts.

Care was also taken that the same lung lobes were used for parallel comparisons, since development and alveolarization is different in apical or basal lung regions^60^.

### Lipidomic profiling of late fetal mouse lungs with high-resolution AP-SMALDI MSI

**Figure 2:**
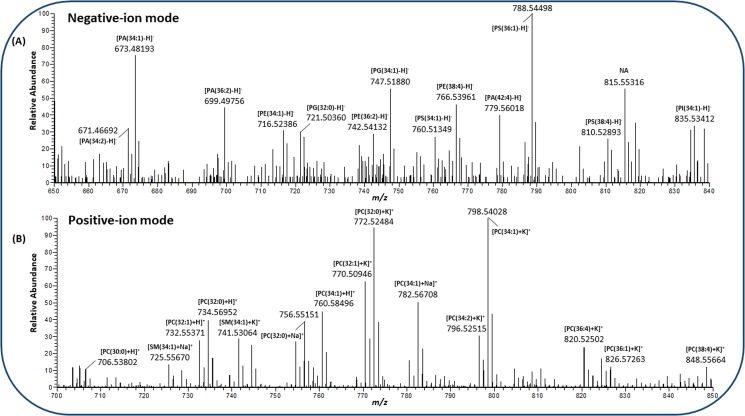
Single-pixel (10 µm) mass spectrum obtained from late fetal E19 mouse lung mass spectrometry imaging (MSI) experiments. (**A**) Negative-ion single-pixel mass spectrum for mass range *m/z* 650–850. (**B**) Positive-ion single-pixel mass spectrum for mass range *m/z* 700–850. Different classes of lipid species were identified based on high mass accuracy (≤2 ppm), labelled with measured mass and charge carrier.

After optimization of the tissue sectioning and selection procedure, lipids were characterized by scanning the E19 (WT) lung tissue sections at a high mass resolution (140,000 @ *m/z* 200) and high spatial resolution (10 µm per pixel) using AP-SMALDI MSI. Figure 2A,B represent a single pixel (10 µm) mass spectrum of E19 mouse lungs for the selected *m/z* range in negative- and positive-ion mode. The negative-ion mode analysis of E19 mouse lungs resulted in detection of different classes of lipid deprotonated species (Fig. 2A), whereas in positive-ion mode, mainly PC and SM lipids were observed as protonated, sodiated, or potassiated species (Fig. 2B). In general, PC, SM and ceramide (Cer) lipid species are predominantly ionized and detected in positive-ion mode, due to their quaternary amine groups, suppressing the ionization of other lipid classes. Phosphatidylglycerol (PG), PE, PS, phosphatidic acid (PA) and phosphatidylinositol (PI), on the other hand, are dominant in negative-ion mode^61^. Therefore, to provide comprehensive lipid information of E19 mouse lungs, MSI experiments were performed in both ionization modes.

In positive-ion spectra (Fig. 2B) the most intense lipid signals were observed at *m/z* 772.52484, 798.54028 and annotated as potassiated PC. The signal at *m/z* 772.52484 with a mass error of 0.60 ppm corresponds to potassiated PC(32:0) lipid. PC(32:0) [DPPC; most probably a combination of two saturated palmitic acyl chains] is the major surface-active component (≈40–70% of total PC or ≈30–60% of total surfactant lipid) of the mammalian pulmonary lipidome and is responsible for generating a lower surface tension (close to zero mN/m) during respiration^10,62^. Protonated PC(32:0) at *m/z* 734.56952 (0.12 ppm) and its sodiated homologue at *m/z* 756.55151 (0.18 ppm) were also detected with very high mass accuracy. In addition to DPPC, di-saturated (30:0, 34:0) and mono-unsaturated (32:1, 34:1) PCs, which are the major PC lipid species of mammalian pulmonary surfactant were also observed as intense peaks and detected with three different charge carriers^63^. Sphingomyelins are essential constituents of plasma membranes and are involved in pathogenesis of various pulmonary diseases^64^. Among the sphingomyelins, SM(34:1) was found to be highly abundant and was detected with three different charge carriers (Fig. 2B). Similarly, other individual lipid species, in the low *m/z* range various lysophospholipids were detected with high mass accuracy.

In negative-ion mode (Fig. 2A), important acidic phospholipids such as PG (the second most abundant phospholipid class in lung surfactant), PE (abundant in whole lung tissue), other minor phospholipid classes (PA, PS, PI etc.), cholesterol sulfate and free fatty acids were detected and annotated based on high mass accuracy. For instance, intense signals at *m/z* 716.52386 (0.27 ppm), 760.51349 (0.13 ppm), 835.53412 (0.11 ppm), 747.51880 (0.80 ppm), 673.48193 (0.74 ppm) were annotated as 34:1 lipid molecular species of PE, PS, PI, PG and PA lipid classes respectively. PG and PI lipids are synthesized from the same precursor (CDP-glycerol) and constitute up to 8–15% of the total surfactant phospholipid pool^10^. They are known to enhance the adsorption and spreading of DPPC over the epithelial surface^63^. The most intense signal in Fig. 2A at *m/z* 788.54498 (0.35 ppm) corresponds to the deprotonated PS(36:1).

**Figure 3:**
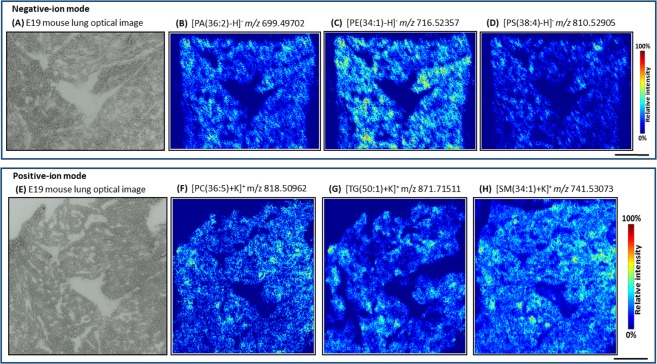
Characterization of late fetal E19 mouse lung lipidome using high-resolution AP-SMALDI mass spectrometry imaging experiments. (**A**,**E**) Microscopic images of E19 mouse lung. (**B**–**D**) Negative-ion MS images. (**B**) [PA(36:2) − H]^−^, (**C**) [PE(34:1) − H]^−^, (**D**) [PS(38:4) − H]^−^. (**F**–**H**) Positive-ion MS images. (**F**) [PC(36:5) + K]^+^, (**G**) [TG(50:1) + K]^+^, (**H**) [SM(34:1) + K]^+^. Scale bar 500 µm.

Late fetal E19 (WT) mouse lung lipid ion MS images were generated using MSiReader software (jet colour map, linear interpolation of zero order and no normalization). Figure 3 shows selected MS images in negative- and positive-ion mode. Figure 3B–D show negative-ion MS images corresponding to *m/z* 699.49702, 716.52386, 810.52905, annotated as [PA(36:2) − H]^−^, [PE(34:1) − H]^−^ and [PS(38:4) − H]^−^ respectively. Similarly, Fig. 3F–H show positive-ion MS images of *m/z* 818.50962, 871.71511, 741.53064, annotated as [PC(36:5) + K]^+^, [TG(50:1) + K]^+^ and [SM(34:1) + K]^+^. With our high-resolution MSI approach, various classes of phospholipids, glycerolipids, and sphingolipids were imaged and annotated based on accurate mass (RMSE ≤2 ppm, Tables S1 & S2, Fig. S2). The majority of the identified lipid species were ubiquitously distributed in the whole E19 mouse lung tissue sections. Due to the dense content of epithelial cells and typical fetal structure of the not yet air-inflated lung tissue, a homogenous lipid distribution pattern (mainly covering the peripheral region) was observed in the respiratory region of the E19 lung (later developing into the alveolar region of the adult lung).

On-tissue tandem mass spectrometry (MS/MS) experiments were performed to confirm the chosen lipid annotations. For instance, on-tissue MS/MS analysis of potassiated SM(34:1) at *m/z* 741.52910 ± 0.4 resulted in diagnostic fragment ions at *m/z* 682.45550 (neutral loss of trimethylamine, 59.0736), *m/z* 558.46357 (neutral loss of phosphocholine, 183.06553), *m/z* 162.95622 (potassiated cyclophosphate, a characteristic fragment ion for potassiated PC and SM), and *m/z* 184.07369 (phosphocholine). Similarly, HCD activation of sodiated SM(34:1) at *m/z* 725.55551 ± 0.4 yielded product ions at *m/z* 666.48207, *m/z* 542.48982 and *m/z* 184.07373. MS/MS analysis of protonated SM(34:1) at *m/z* 703.57337 ± 0.4 produced the predominant fragment ion at *m/z* 184.07373 (phosphocholine).

MS/MS analyses of lipid species in negative-ion mode allowed assignment of individual fatty acyl (FA) chains. For example, fragmentation of [PE(34:1) − H]^−^ at *m/z* 716.52344 ± 0.4 produced fragment ions at *m/z* 255.23252 (16:0; palmitic acid) and *m/z* at 281.24821 (18:1; oleic acid). On-tissue MS/MS analysis of [PA(36:2) − H]^−^ at *m/z* 699.49744 ± 0.4 showed an intense fragment ion at *m/z* 281.24843 (18:1; oleic acid). Similarly, different classes of lipid species showed diagnostic fragment ions for the individual lipid class (Table S3 and MS/MS spectra in Supplementary Data). For example, the most intense peak in Fig. 2A, PS(36:1) at *m/z* 788.54397 ± 0.4 yielded a fragment ion at *m/z* 701.51242 (loss of serine, 87.03155) which is characteristic for phosphatidylserine.

Taken together, our optimized method was able to detect and characterize all possible major lipid species from E19 mouse lungs, based on accurate mass and on-tissue MS/MS analysis in positive- and negative-ion mode. This lipidomic information may aid as a reference for the lung lipidome, for a better understanding of late stage fetal lung development and differentiation as well as of the molecular mechanisms of various pulmonary diseases associated with lipid alterations.

### Optimization of data processing framework for comparative lipidomics

**Figure 4:**
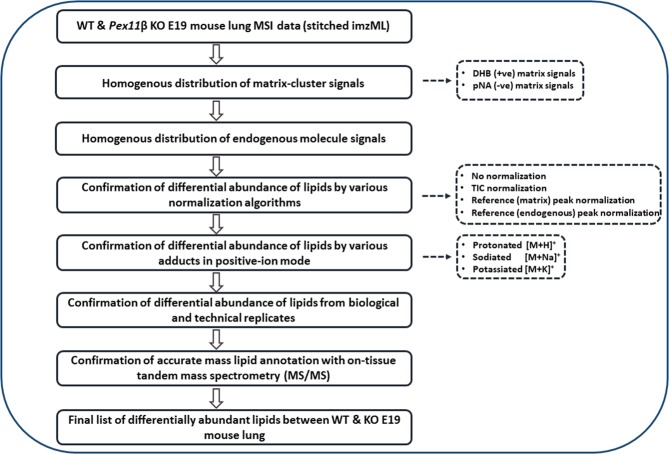
Scheme of the optimized data processing framework.

After lipidomic characterization of E19 (WT) mouse lungs, we optimized and established a data processing framework for the relative comparison of signal intensities of analytes in different tissue sections. A detailed scheme of the proposed data processing workflow is depicted in Fig. 4. Briefly, equally thick E19 WT and *Pex11β* KO lung tissue sections with optimal morphology were analyzed on consecutive days (biological and technical replicates) with identical sample preparation, experimental and instrumental conditions. The single MS raw files were converted to imzML files and stitched together. Data analysis and optimization of various parameters for the relative quantification of lipids using MALDI MSI is described in the following sections.

### Homogenous deposition of matrix

**Figure 5:**
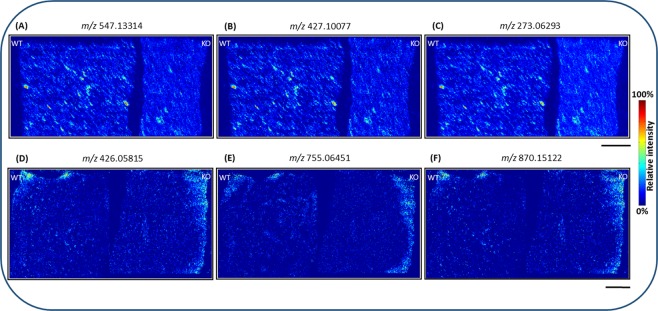
Distribution of matrix cluster signals in wild type (WT) and *Pex11β* knockout (KO) late fetal E19 mouse lung tissue sections from mass spectrometry imaging (MSI) experiments. (**A**–**C**) Evenly distributed 4-Nitroaniline (pNA) matrix cluster images in negative-ion mode. (**D**–**F**) Evenly distributed 2,5-Dihydroxybenzoic acid (DHB) matrix cluster images in positive-ion mode. Scale bar 500 µm.

In addition to the tissue quality and thickness of the tissue sections, matrix deposition is another critical parameter in MSI quantification. In order to compare signal intensities of analytes in two different tissue (WT and KO) sections, equal concentration and homogenous deposition of matrix on both tissue sections is necessary. In the present study, we obtained homogenously sprayed matrix crystals on tissue surfaces (crystal size ≤10 µm, Fig. S3) by optimizing the flow rate of the spray syringe (10 µl/min), nitrogen gas (1 bar), and rotation speed of the sample probe for pNA and DHB matrices using the SMALDIPrep system in negative- and positive-ion mode. No washing steps were performed before matrix deposition. Homogeneous distribution of pNA and DHB matrix in WT and KO E19 lung tissue sections in negative- and positive-ion mode are illustrated in Figs 5 & S4.

Figure 5A–C show the homogeneous distribution of pNA matrix clusters (A) *m/z* 547.13314 [4(pNA − H)^−^], (B) *m/z* 427.10077 [3(pNA − H)^−^ + H_2_O] and (C) *m/z* 273.06293 [2(pNA − H)^−^] between WT and KO tissue sections. Similarly, Fig. 5D–F demonstrate the homogeneous distribution of DHB matrix clusters (D) *m/z* 426.05815 [3DHB-2H_2_O]^+^, (E) *m/z* 755.06451 [5DHB-3H_2_O + K]^+^ and (F) *m/z* 870.15122 [6DHB-4H2O + NH4]^+^ among WT and KO tissue sections. Uneven matrix deposition and crystallization creates artefacts in MSI, influencing signal intensities of analytes. Therefore, in our study we carefully optimized the matrix application to attain uniform deposition of matrix layers on both WT and KO tissue section surfaces. This enabled us to obtain high-quality and reproducible results from MSI for semi-quantification (relative quantification) of biomolecules. Recently, we showed that pneumatic spraying of matrix increases the lipid signal intensities 8-fold and 30-fold, compared to sublimation-recrystallization and sublimation, respectively^51^. In addition, reproducible MSI data (in terms of number and intensity of ion signals, pixel coverage and image quality) can be obtained from samples prepared by automated pneumatic spraying^51^.

**Figure 6:**
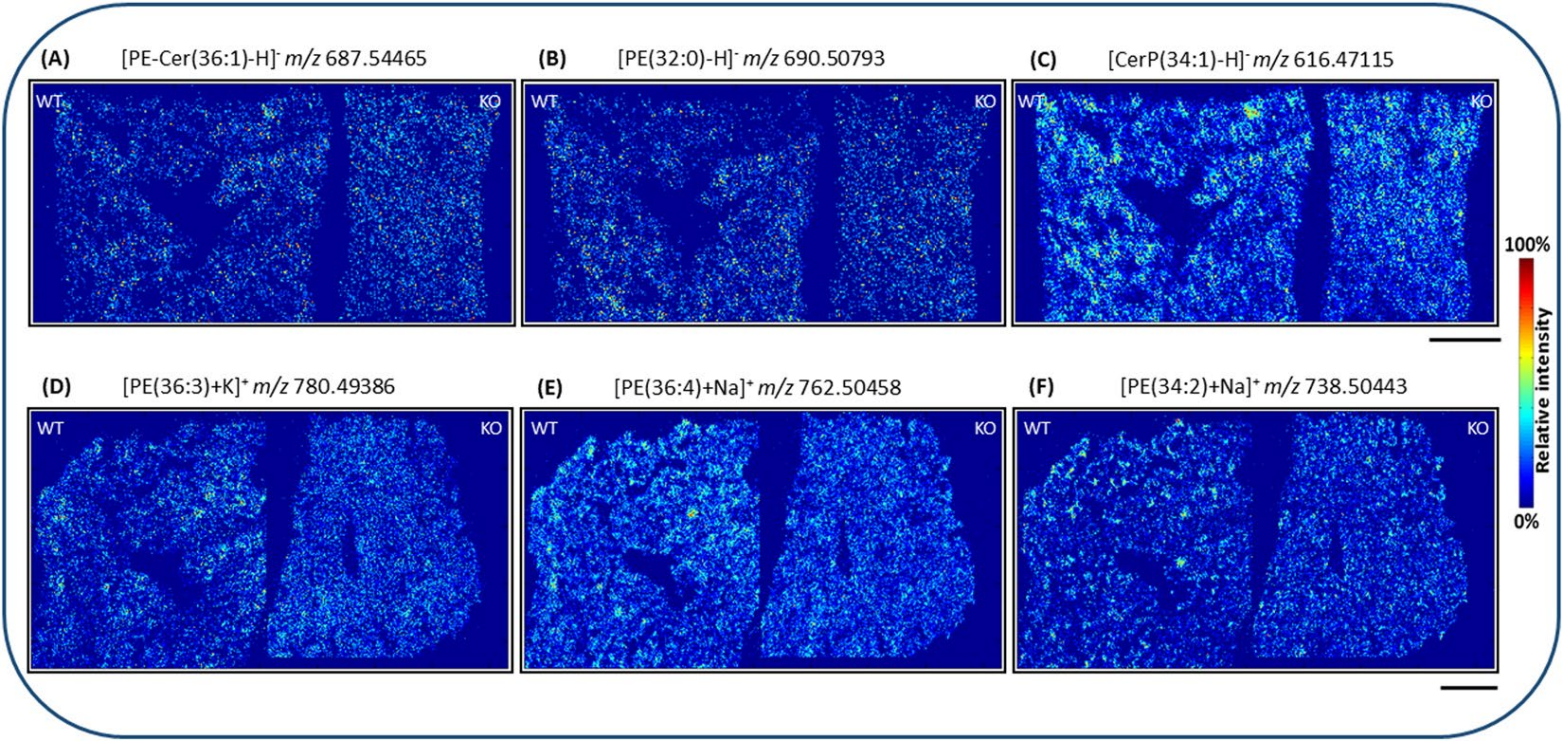
Homogenous distribution of endogenous compounds in wild type (WT) and *PEX11β* knockout (KO) late fetal E19 mouse lung tissue sections, measured by atmospheric-pressure MALDI MSI experiments. (A-C) Evenly distributed lipid species in negative-ion mode. (**A**) [PE-Cer(36:1) − H]^−^, (**B**) [PE(32:0) − H]^−^ and (**C**) [CerP(34:1) − H]^−^. (**D**–**F**) Evenly distributed lipid species in positive-ion mode. (**D**) [PE(36:3) + K]^+^, (**E**) [PE(36:4) + Na]^+^ and (**F**) [PE(34:2) + Na]^+^. Scale bar 500 µm.

In addition to matrix signals, we also detected endogenous molecules that were distributed uniformly in E19 WT and KO lung tissue sections in negative- and positive-ion mode. For instance, Fig. 6A–C) show even distribution of PE-Cer(36:1), PE(32:0) and CerP(34:1) at *m/z* 687.54465, 690.50793 and 616.47115 in negative-ion mode. Similarly, Fig. 6D–F) show positive-ion MS images of *m/z* 780.49386, 762.50458, 738.50443, corresponding to potassiated PE(36:3), sodiated PE(36:4) and sodiated PE(34:2), respectively. Uniform distributions of these endogenous molecules in WT and KO were confirmed for all three charge carriers, H^+^, Na^+^ and K^+^ in positive-ion mode. Homogenous distributions of the three differently charged PE(36:4) and PE(34:2) in WT and KO tissue sections are demonstrated in Fig. S5.

### Differential abundance of lipid species between E19 WT and *Pex11β* KO mouse lungs

**Figure 7:**
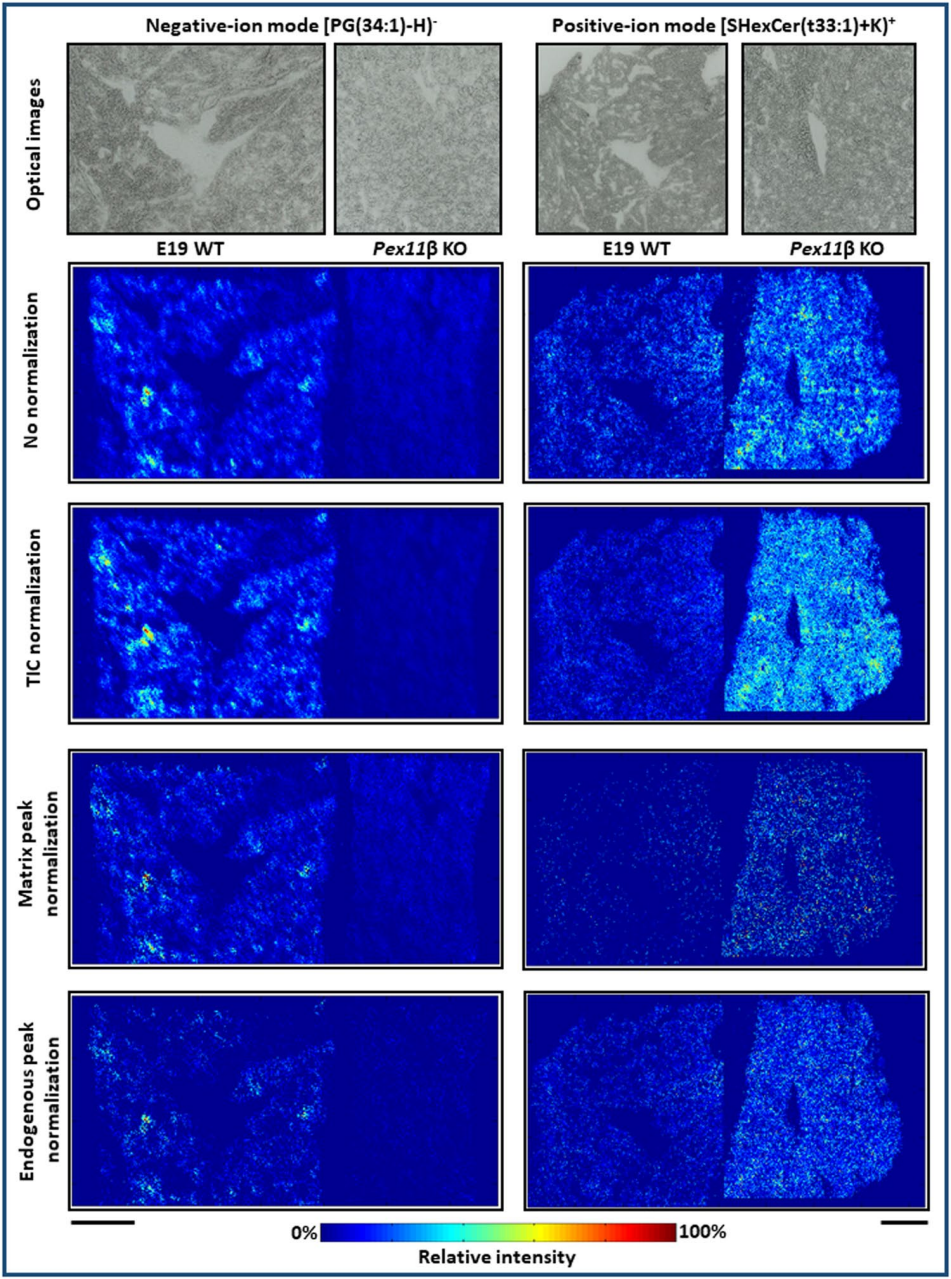
Differential abundance of lipid species in wild type (WT) and *Pex11β* knockout (KO) late fetal E19 mouse lung tissue sections in negative- and positive-ion mode. Downregulation of [PG(34:1) − H]^−^ lipid (*m/z* 747.51815), upregulation of [SHexCer(t33:1) + K]^+^ lipid (*m/z* 820.46415) in *Pex11β* KO E19 mouse lungs in negative- and positive-ion mode. The abundance comparisons between lipids in different cryosections were verified by applying various normalization algorithms: (i) no normalization, (ii) total ion count (TIC) normalization, (iii) normalization to homogenously distributed matrix signals and (iv) normalization to homogenously distributed endogenous signals. Scale bar 500 µm.

Our results showed a wide variety of lipid species and other cellular metabolites that were differentially expressed between WT and *Pex11β* KO E19 mouse lung tissue sections. For instance, in KO tissue sections, deprotonated PG(34:1) (*m/z* 747.51815) showed lower signal intensities in comparison to WT (Fig. 7). Phosphatidylglycerol (PG) is the second most abundant phospholipid in pulmonary surfactant, with PG(34:1) being the predominant lipid molecular species, playing a role in adsorption, alveolar stability and innate immunity of the lungs^9,10,14^. Furthermore, recent studies demonstrated that PG(34:1) (palmitoyl-oleoyl-phosphatidylglycerol, POPG) acts as a potent antiviral agent against influenza A and respiratory syncytial viruses^13,15^ as well as antagonizes the proinflammatory actions of mycoplasma pneumoniae^65^. In positive-ion mode, potassiated sulfatide lipid (SHexCer (t33:1), *m/z* 820.46415, based on MS1 annotation) showed differentially higher signal intensities in KO tissue sections compared to WT (Fig. 7). Sulfatides are multifunctional molecules, playing important roles in physiological processes of various organs. It has been shown that sulfatide (glycolipids) levels were elevated in many human cancer cells and tissues including pulmonary adenocarcinoma^66^. In our study, MS images clearly illustrate the differences in signal intensities between WT and KO lung tissues.

In addition to biological variations, several experimental and instrumental parameters may also cause variations in signal intensities across different imaging datasets. Therefore, to prove the correctness of observed biological variations from *Pex11β* E19 mouse lungs and to exclude technique-dependent differences between distinct cryosections, we applied several different normalization algorithms during image generation. In our study, MS images were generated (1) without normalization, (2) with normalization to total ion current (TIC), (3) with normalization to uniformly distributed matrix cluster signals, and (4) with normalization to uniformly distributed endogenous signals. Figure 7 shows MS images of differentially abundant lipid species in WT and *Pex11β* KO tissue sections, generated with various normalization algorithms. Regardless of the normalization method, our MSI data was found to be consistent, and normalization approaches did not induce significant changes in the determined differential abundance of lipid species in both ionization modes, proving the high quality of our optimized protocol used (Fig. 7 & Table S4).

Normalization is defined as a process of projecting all data to a common intensity scale for the comparison of spectra^67,68^. Many normalization strategies have been described in literature to correct the variations in signal intensities of MSI data such as TIC, root mean square (RMS), median intensity, vector normalization, normalization to matrix signals, endogenous signals, stable isotope standards, tissue-specific ionization efficiency coefficient and sliding window normalization, etc.^67–71^. Each strategy has its own limitations, a number of them are reviewed in detail in the context of quantification (relative and absolute) of low molecular weight compounds by MALDI MSI^71^.

TIC normalization is the most common method, and several studies demonstrated uniformly distributed matrix cluster and/or endogenous signals as “internal-standard-like” normalization for semi-quantitative analysis by MALDI MSI^68^. In many cases, normalization, interpolation and other post-processing parameters influence signal intensities of analytes in MSI, which often leads to wrong interpretations of the data. In such cases, it is challenging to draw reliable biological conclusions from MSI data, especially in the case of biomarker studies and pharmaceutical applications (drug discovery). Therefore, we optimized the sample preparation, experimental and instrumental conditions, and our data processing framework very carefully to produce highly accurate and reproducible MSI data for a direct relative comparison of signal intensities of analytes.

The differential abundance of lipid species in WT and KO tissue sections was confirmed using biological replicates (Fig. S6). For instance, deprotonated PG (34:1) and potassiated SHexCer (t33:1) showed similar trends of differential abundance between WT and KO tissue sections of different animals (n = 3).

### Confirmation of differential abundance of lipids with various ion adducts

**Figure 8:**
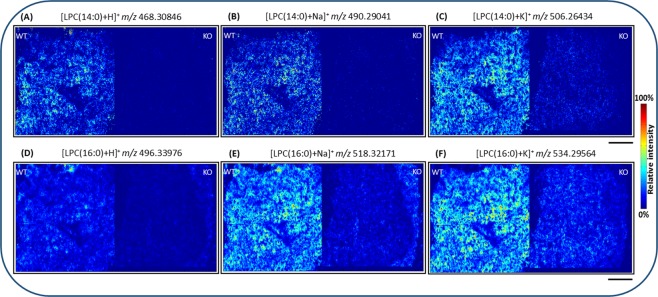
Confirmation of differential abundance of lipid species in wild type (WT) and *Pex11β* knockout (KO) late fetal E19 mouse lung tissue sections for various charge carriers in positive-ion mode. (**A**–**C**) Differential abundance of LPC(14:0) in WT and KO E19 mouse lung tissue. (**A**) [LPC(14:0) + H]^+^, (**B**) [LPC(14:0) + Na]^+^, (**C**) [LPC(14:0) + K]^+^. (**D**–**F**) Differential abundance of LPC(16:0) in WT and KO E19 mouse lung tissue. (**D**) [LPC(16:0) + H]^+^, (**E**) [LPC(16:0) + Na]^+^, (**F**) [LPC(16:0) + K]^+^. Scale bar 500 µm.

Differential abundance of lipid species in WT and KO tissue sections was also confirmed based on various ionic species in positive-ion mode. For instance, the lysophosphatidylcholines LPC(14:0) and LPC(16:0) exhibited reduced signal intensities in KO lung tissue sections compared to WT. LPCs are lipid mediators produced from PCs by the action of phospholipase A2 (PLA2), and involved in various pro-inflammatory and pro-atherogenic activities^72^. The observed differential pattern was found to be consistent in protonated, sodiated and potassiated ionic state. Figure 8 shows MS images of LPC(14:0) [(A) 468.30846, (B) 490.29041 and (C) 506.26434] and LPC(16:0) [(D) 496.33976, (E) 518.32171 and (F) 534.29564], containing the three different charge carriers, respectively.

### Structural confirmation of lipid species using on-tissue tandem mass spectrometry (MS/MS)

**Figure 9:**
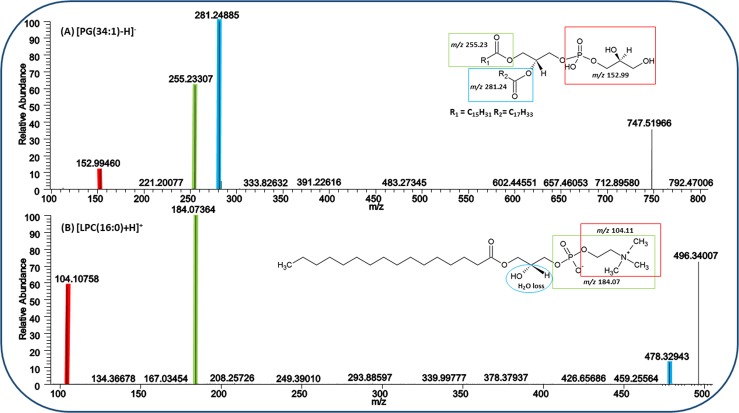
Structural confirmation of lipid annotations by on-tissue tandem mass spectrometry (MS/MS) in negative- and positive-ion mode, using high-energy collisional dissociation (HCD). (**A**) On-tissue HCD-MS/MS (averaged) spectrum of [PG(34:1) − H]^−^ *m/z* 747.51966 ± 0.4. (**B**) On-tissue HCD-MS/MS (averaged) spectrum of [LPC(16:0) + H]^+^ *m/z* 496.34007 ± 0.4.

The web-based “MetFrag” server was used to generate possible fragment ions of the lipid species^73^. The theoretical fragments generated by the software were identical to the fragments observed in our experiments. Annotation of differentially abundant lipids was further confirmed based on on-tissue tandem mass spectrometry experiments. For instance, deprotonated PG(34:1) (POPG) at *m/z* 747.51966 ± 0.4 was structurally confirmed with fragment ions at *m/z* 152.99460 (corresponding to the cyclic phosphate anion, red), *m/z* 255.23307 (corresponding to palmitic fatty acyl (FA 16:0), green), and *m/z* 281.24885 (corresponding to oleic fatty acyl (FA 18:1), blue) (Fig. 9A).

In positive-ion mode, protonated LPC(16:0) at *m/z* 496.34007 ± 0.4 was structurally confirmed using fragment ions at *m/z* 104.10758 (corresponding to choline, red), *m/z* 184.07364 (corresponding to phosphocholine head group, green), and *m/z* 478.32943 (corresponding to the loss of water [M + H-H_2_O]^+^, blue) (Fig. 9B). Similarly, sodiated and potassiated species were also confirmed with characteristic fragmentation patterns from both WT and KO tissue sections (Supplementary Data & Table S3).

## Conclusion

We report a comprehensive lipid map of late fetal WT mouse lungs at day 19 of gestation (E19) using high-resolution accurate mass AP-SMALDI MSI. We demonstrated optimized experimental and instrumental conditions as well as a detailed data processing framework for comparative analysis of biomolecules in different tissue sections of WT and KO mice (using the *Pex11β* KO mouse here as a model system). The optimized workflow will be useful for relative (semi-) quantitative analyses of lipids and other cellular metabolites from different tissue sections using MALDI MSI. Highly specific and accurate lipidomic information of late fetal stage E19 mouse lungs may serve as a reference for better understanding of lung developmental processes (e.g. late fetal differentiation) or molecular mechanisms of various pulmonary diseases, associated with lipid alterations.

## Materials and Methods

### Materials

Trifluoroacetic acid (TFA, spectroscopy grade), 2,5-Dihydroxybenzoic acid (DHB, 98% purity) and acetone (spectroscopy grade) were purchased from Merck (Darmstadt, Germany). 4-Nitroaniline (pNA, ≥99% purity) and water (LC-MS grade) were procured from Sigma-Aldrich (Steinheim, Germany). Glass microscope slides (ground edges, SuperFrost) were obtained from R. Langenbrinck (Emmendingen, Germany). All other reagents used were of highest purity and analytical grade.

### Animal experiments

Specific-pathogen-free (SPF) C57BL/6J mice were obtained from Charles River, Sulzfeld, Germany. Mice were kept on a regular laboratory diet and water *ad libitum* and housed in cages under standardized environmental conditions (12 hours light/dark cycle, 23 °C ± 1 °C and 55% ± 1% relative humidity). Generation and breeding of *Pex11β* knockout (KO) mice were described previously^53,54^. Pregnant dames with eventual *Pex11β* KO mice (autosomal-recessive inheritance with 25% WT, 50% *Pex11β* HTZ, and 25% *Pex11β* KO) were kept in the SPF central animal facility of Justus Liebig University Giessen, Germany. Pregnant dames were killed by cervical dislocation and the E19 fetus were killed by decapitation.

“Experiments were approved by the named animal welfare officers of the Justus Liebig University (administrative number M471) and performed according to the German and European animal welfare law”.

### Sample (E19 mouse lung tissue) preparation for MSI

For the perfusion of the E19 mouse lungs, the skin of the pups was opened, the right ventricle of the heart was punctured with an 18-size gauze needle. Thereafter, the left atrium was cut open and the animals were perfused via the right ventricle with 500 μl of saline. After the saline wash, lung tissues were perfused with 10 ml of a 4% paraformaldehyde (PFA) fixative in phosphate buffer saline (PBS), pH 7.4. Thereafter, the lungs were excised and further fixed from the outside by overnight immersion fixation. The PFA fixed lung tissues were embedded with the optimal cutting temperature (OCT) compound. Twelve µm thick cryosections were cut with a cryomicrotome (CM 3050 S cryostat, Leica Microsystems, Nussloch, Germany) and stored at −80 °C until further use.

Later, we optimized a sectioning procedure for fresh snap-frozen E19 mouse lung tissues without using any embedding material. For cryosectioning, fresh frozen E19 lung tissues were mounted directly on a sample holder of the cryotome, using deionized water (ice) as an adhesive. For MSI experiments, both WT and *Pex11β* KO E19 mouse lung tissue, serial sections were cut with an equal thickness of 12 µm at −20 °C with a cryomicrotome (CM 3050 S cryostat, Leica Microsystems, Nussloch, Germany). One section of a series was thaw-mounted on each glass microscope slide (ground edge, SuperFrost) and labelled with an appropriate number for later identification. For the purpose to get an overview on the quality and thickness of each entire cryosection, several overlapping images covering the whole tissue section were taken at a magnification of 40x with a Leica DMRD microscope (Leica, Bensheim, Germany) equipped with a Leica DC 480 camera (Leica, Bensheim, Germany) and thereafter stitched together with Image composite editor (Microsoft Research). Identical camera settings were used to be able to compare the darkness and grey values of all cut cryosections to optimize the selection procedure for high quality sections with similar section thicknesses. Care was taken to speed up the image taking procedure of each individual section. All other sections were stored in parallel prior to and after photographing in a slide box on dry ice. The best and optimally matched lung cryosections of the appropriate WT and KO animals were either used directly for immediate MS imaging experiments or stored at −80 °C until further analysis on consecutive days for replicate measurements.

### Matrix application

Prior to matrix application, the frozen lung tissue sections were brought to room temperature in a desiccator (about 15 minutes) to avoid condensation of humidity on the surface of the samples. DHB (30 mg mL^−1^ in acetone:water at 1:1 vol/vol, 0.1% TFA), and pNA (10 mg mL^−1^ in acetone:water at 1:1 vol/vol) solutions were prepared freshly for the matrix application. For positive-ion mode measurements, a volume of 120 µL of DHB, for negative-ion mode a volume of 50 µL of pNA matrix solution were deposited homogeneously on the tissue surfaces by using an automatic pneumatic ultrafine sprayer (“SMALDIPrep”, TransMIT GmbH, Giessen, Germany)^74^.

### MALDI mass spectrometry imaging

Immediately after matrix application, MSI experiments were performed using a high-resolution atmospheric-pressure scanning microprobe matrix-assisted laser desorption/ionization ion source (“AP-SMALDI10”, TransMIT GmbH, Giessen, Germany), coupled to a Fourier transform orbital trapping mass spectrometer (Q Exactive, Thermo Scientific GmbH, Bremen, Germany)^75^. Desorption and ionization of analytes were initiated by a nitrogen gas laser (LTB Lasertechnik GmbH, Berlin, Germany) with a wavelength of 337 nm, operating at a repetition rate of 60 Hz. The laser beam was focused coaxially with the ion beam by a centrally bored objective lens^76^. In all the MALDI MSI experiments, the E19 mouse lung tissue sections were measured with high resolution in mass (140,000 @ *m/z* 200) and space (10 µm per pixel). The step size of the sample stage was set to the desired pixel size (10 µm). The mass spectrometer was operated in positive- and negative-ion mode in the mass to charge number (*m/z*) range of *m/z* 250–1000 with a target voltage of +/−4.3 kV. Automatic gain control (AGC) was disabled, and the injection time (IT) was fixed at 500 ms (microscans = 1). The measurement speed in full scan mode (scan range *m/z* 250–1000) was about 0.8 pixels per second at a mass resolution of 140,000 (at *m/z* 200). Ions formed by 30 laser pulses per spot were accumulated in the C-trap before being sent to the orbitrap mass analyser for detection. Internal mass calibration was performed using known matrix ion signals as lock mass values (716.12462 and 409.09020 for positive- and negative-ion mode, respectively), which resulted in a mass accuracy better than 2 ppm root mean square error (RMSE) over the entire measurement.

### Annotation of lipid species

Lipid species were annotated according to the proposal for shorthand notation of lipid structures derived from mass spectrometry^77^. For instance, phospholipid species (e.g. PC, PG, PI, etc.) were denoted as (X:Y), where X represents the total number of carbon atoms and Y represents the total number of double bonds in fatty acyl chains, without specifying the individual acyl chains attached to the glycerol or sphingosine backbone. In sphingolipids, “m”, “d” and “t” represents the number of hydroxyl groups in sphingoid base (mono, di, and tri)^77^.

### Data processing and image generation

AP-SMALDI MSI data sets (.raw) were converted to centroid imzML files, and E19 mouse lung lipid ion MS images were generated using the open source software “MSiReader” version 0.09^78^ with a *m/z* bin width of Δ *m/z* = ±5 ppm. No further (pre or post) data processing steps were applied for image generation in order to demonstrate the original data quality. The lipid species were annotated based on accurate ion mass (*m/z*) values, by using databases such as LIPID MAPS (www.lipidmaps.org), METLIN (www.metlin.scripps.edu) and Human Metabolite Database (www.hmdb.ca) within Δ *m/z* = ±2 ppm or 0.0005 Da. In negative-ion mode, deprotonated [M − H]^−^, in positive-ion mode protonated [M + H]^+^, sodiated [M + Na]^+^ and potassiated [M + K]^+^ species were considered for the annotation.

After characterization of the lipidome of E19 mouse lungs, signal intensities of lipid species were compared between E19 WT and *Pex11β* KO mouse lung tissue sections using the same software (MSiReader v0.09). Images were generated with a bin width of Δ *m/z* = ±5 ppm. Pre-processing steps, such as baseline correction, noise removal, smoothing or interpolation, were not applied during image generation. Normalization of signal intensities was performed in the following alternative ways: (1) no normalization, (2) total ion current (TIC) normalization, (3) normalization with ubiquitous matrix signals, (4) normalization with ubiquitous endogenous signals, or (5) specific strategies for comparison of relative ion abundances in WT and KO E19 lung tissue sections. These procedures are explained in detail in the results and discussion section.

### On-tissue MS/MS analysis

Lipid annotation based on accurate ion mass (≤2 ppm) was further confirmed by on-tissue tandem mass spectrometry (MS/MS) analysis. MS/MS fragmentation was performed by using higher-energy collisional dissociation (HCD) with a precursor ion isolation window of 0.4 Da. Fragmentation of individual lipid species was obtained with normalized collision energy values (NCE) of 20–28% (averaged spectra from 20, 25 and 28 NCE) and 15–25% (averaged spectra from 15, 20 and 25 NCE) in positive- and negative-ion mode, respectively. The experimental fragmentation patterns of lipids were confirmed based on neutral losses, diagnostic fragment ions of the head groups^79^, by matching with tandem MS spectra of standard lipid species available in databases (e.g. METLIN, HMDB, etc.), or by comparing with the in-silico fragmentation patterns using MetFrag web server^73^.

## Supplementary information

Supplementary information
