# Supplementary material for: High-resolution atmospheric-pressure MALDI mass spectrometry imaging workflow for lipidomic analysis of late fetal mouse lungs
Source: Sci Rep. 2019 Feb 28;9:3192. doi: 10.1038/s41598-019-39452-3 (PMC6395778; doi:10.1038/s41598-019-39452-3)
Supplement: Supplementary file 1 — Supplementary information [file 41598_2019_39452_MOESM1_ESM.pdf]

SUPPLEMENTARY INFORMATION

High-resolution atmospheric-pressure MALDI mass spectrometry imaging workflow for lipidomic analysis of late fetal mouse lungs

Vannuruswamy Garikapati[1, 2], Srikanth Karnati[2, #], Dhaka Ram Bhandari[1], Eveline Baumgart-Vogt[2], Bernhard Spengler[1*]

Table of contents: **Page No.**

Supplementary Table 1……………………………………………2-7

Supplementary Table 2……………………………………………7-14

Supplementary Table 3……………………………………………14-17

Supplementary Data…………………………………………………18-69

Supplementary Figure 1…………………………………………...70

Supplementary Figure 2……………………………………………71

Supplementary Figure 3……………………………………………72

Supplementary Figure 4……………………………………………73

Supplementary Figure 5……………………………………………74

Supplementary Table 4……………………………………………..75

Supplementary Figure 6…………………………………………….76

List of annotated lipid species in negative- (supplementary table 1) and positive-ion mode (supplementary table 2). The lipid species identified (annotated) in both-ion modes are highlighted in *italic* letters.

Supplementary Table 1: List of lipid species annotated in negative-ion mode, corresponding molecular formula, ion adducts, theoretical *m/z* values and root mean square error (RMSE) values in parts per million (ppm). Lipid annotations are based on accurate *m/z* values (RMSE ≤ 2ppm) and diagnostic fragment ions from on-tissue fragmentation (MS/MS) experiments.

S. No	Lipid annotation	Molecular Formula	Ion adduct	Theoretical (*m/z*)	RMSE (ppm)
1	Palmitoleic acid	C16H30O2	$[M-H]^-$	253.21726	1.25
2	Palmitic acid	C16H32O2	$[M-H]^-$	255.23291	1.15
3	Linoleic acid	C18H32O2	$[M-H]^-$	279.23291	0.85
4	Oleic acid	C18H34O2	$[M-H]^-$	281.24856	0.72
5	Stearic acid	C18H36O2	$[M-H]^-$	283.26421	0.64
6	Arachidonic acid	C20H32O2	$[M-H]^-$	303.23291	0.77
7	Docosahexaenoic acid	C22H32O2	$[M-H]^-$	327.23291	1.30
8	CPA(16:0)	C19H37O6P	$[M-H]^-$	391.22546	0.69
9	LPA(16:0)	C19H39O7P	$[M-H]^-$	409.23602	0.78
10	CPA(18:1)	C21H39O6P	$[M-H]^-$	417.24111	0.65
11	CPA(18:0)	C21H41O6P	$[M-H]^-$	419.25676	0.70
12	LPA(18:1)	C21H41O7P	$[M-H]^-$	435.25167	0.70
13	LPA(18:0)	C21H43O7P	$[M-H]^-$	437.26732	0.79
14	LPE(16:0)	C21H44NO7P	$[M-H]^-$	452.27822	0.76
15	Cholesterol sulfate	C27H46O4S	$[M-H]^-$	465.30437	0.82
16	*LPE(18:1)*	*C23H46NO7P*	$[M-H]^-$	478.29387	0.76

17	LPS(P-16:0)	C22H44NO8P	[M-H]$^-$	480.27314	0.73
18	*LPE(18:0)*	*C23H48NO7P*	[M-H]$^-$	480.30952	0.63
19	LPG(16:0)	C22H45O9P	[M-H]$^-$	483.27280	0.96
20	*LPE(20:4)*	*C25H44NO7P*	[M-H]$^-$	500.27822	0.79
21	LPG(18:1)	C24H47O9P	[M-H]$^-$	509.28849	1.13
22	*LPE(22:6)*	*C27H44NO7P*	[M-H]$^-$	524.27822	1.14
23	LPS(18:0)	C24H48NO9P	[M-H]$^-$	524.29935	0.80
24	*LPE(22:4)*	*C27H48NO7P*	[M-H]$^-$	528.30952	0.95
25	PA(24:1)	C27H51O8P	[M-H]$^-$	533.32484	1.02
26	PA(26:1)	C29H55O8P	[M-H]$^-$	561.35614	0.94
27	LPG(22:2)	C28H53O9P	[M-H]$^-$	563.33540	0.92
28	PS(20:0)	C26H50NO10P	[M-H]$^-$	566.30992	1.05
29	LPS(22:1)	C28H54NO9P	[M-H]$^-$	578.34630	1.09
30	LPG(24:3)	C30H55O9P	[M-H]-	589.35105	1.23
31	LPG(24:2)	C30H57O9P	[M-H]$^-$	591.36670	1.45
32	PS(22:0)	C28H54NO10P	[M-H]$^-$	594.34122	1.10
33	LPI(18:1)	C27H51O12P	[M-H]$^-$	597.30450	1.55
34	LPI(18:2)	C27H53O12P	[M-H]$^-$	599.32015	1.51
35	LPS(24:1)	C30H58NO9P	[M-H]$^-$	606.37760	1.48
36	PG(24:1)	C30H57O10P	[M-H]$^-$	607.36162	1.60
37	PA(30:2)	C33H61O8P	[M-H]$^-$	615.40309	1.36
38	CerP(d34:1)	C34H68NO6P	[M-H]$^-$	616.47111	1.10
39	LPG(26:3)	C32H59O9P	[M-H]$^-$	617.38235	1.27
40	PA(30:1)	C33H63O8P	[M-H]$^-$	617.41874	1.30
41	PA(30:0)	C33H65O8P	[M-H]$^-$	619.43439	1.18
42	PS(24:1)	C30H56NO10P	[M-H]$^-$	620.35687	1.18
43	PS(24:0)	C30H58NO10P	[M-H]$^-$	622.37252	1.06
44	LPS(26:1)	C32H62NO9P	[M-H]$^-$	634.40890	1.40

45	CerP(d36:3)	C36H68NO6P	$[M-H]^-$	640.47111	1.40
46	CerP(d36:2)	C36H70NO6P	$[M-H]^-$	642.48676	1.15
47	*PA(32:2)*	*C35H65O8P*	$[M-H]^-$	643.43439	1.46
48	CerP(d36:1)	C36H72NO6P	$[M-H]^-$	644.50241	1.79
49	*PA(32:1)*	*C35H67O8P*	$[M-H]^-$	645.45004	1.26
50	PA(32:0)	C35H69O8P	$[M-H]^-$	647.46569	1.49
51	PS(26:1)	C32H60NO10P	$[M-H]^-$	648.38817	1.50
52	PS(26:0)	C32H62NO10P	$[M-H]^-$	650.40382	1.49
53	PI(22:1)	C31H59O12P	$[M-H]^-$	653.36710	1.57
54	*PA(34:3)*	*C37H67O8P*	$[M-H]^-$	669.45004	1.58
55	*PA(34:2)*	*C37H69O8P*	$[M-H]^-$	671.46569	1.49
56	PS(28:3)	C34H60NO10P	$[M-H]^-$	672.38817	1.70
57	*PA(34:1)*	*C37H71O8P*	$[M-H]^-$	673.48134	0.87
58	LPI(24:1)	C33H63O12P	$[M-H]^-$	681.39840	1.46
59	PE-Cer(d36:1)	C38H77N2O6P	$[M-H]^-$	687.54461	1.36
60	*PE(32:1)*	*C37H72NO8P*	$[M-H]^-$	688.49224	1.16
61	*PE(32:0)*	*C37H74NO8P*	$[M-H]^-$	690.50789	1.40
62	PG(30:1)	C36H69O10P	$[M-H]^-$	691.45552	1.56
63	PG(30:0)	C36H71O10P	$[M-H]^-$	693.47117	1.27
64	PA(36:4)	C39H69O8P	$[M-H]^-$	695.46569	1.25
65	*PA(36:3)*	*C39H71O8P*	$[M-H]^-$	697.48134	1.33
66	*PA(36:2)*	*C39H73O8P*	$[M-H]^-$	699.49699	1.18
67	*PE(P-34:1) / PE(O-34:2)*	*C39H76NO7P*	$[M-H]^-$	700.52862	1.40
68	PA(36:1)	C39H75O8P	$[M-H]^-$	701.51264	1.61
69	*PE(34:2)*	*C39H74NO8P*	$[M-H]^-$	714.50789	1.40
70	*PE(34:1)*	*C39H76NO8P*	$[M-H]^-$	716.52354	1.29
71	PG(32:2)	C38H71O10P	$[M-H]^-$	717.47117	1.58
72	*PE(34:0)*	*C39H78NO8P*	$[M-H]^-$	718.53919	1.71

73	PG(32:1)	C38H73O10P	$[M-H]^-$	719.48682	1.30
74	*PA(38:5)*	*C41H71O8P*	$[M-H]^-$	721.48134	1.84
75	PG(32:0)	C38H75O10P	$[M-H]^-$	721.50247	1.35
76	*PA(38:4)*	*C41H73O8P*	$[M-H]^-$	723.49699	1.32
77	*PA(38:3)*	*C41H75O8P*	$[M-H]^-$	725.51264	1.65
78	CerP(d42:2)	C42H82NO6P	$[M-H]^-$	726.58066	1.56
79	*PA(38:2)*	*C41H77O8P*	$[M-H]^-$	727.52829	1.63
80	PS(32:1)	C38H72NO10P	$[M-H]^-$	732.48207	1.59
81	PS(32:0)	C38H74NO10P	$[M-H]^-$	734.49772	1.79
82	PE(36:5)	C41H72NO8P	$[M-H]^-$	736.49224	1.56
83	*PE(36:4)*	*C41H74NO8P*	$[M-H]^-$	738.50789	1.49
84	*PE(36:3)*	*C41H76NO8P*	$[M-H]^-$	740.52354	1.72
85	*PE(36:2)*	*C41H78NO8P*	$[M-H]^-$	742.53919	1.47
86	PG(34:3)	C40H73O10P	$[M-H]^-$	743.48682	1.49
87	*PE(36:1)*	*C41H80NO8P*	$[M-H]^-$	744.55484	1.61
88	PG(34:2)	C40H75O10P	$[M-H]^-$	745.50247	1.43
89	*PG(34:1)*	*C40H77O10P*	$[M-H]^-$	747.51812	1.38
90	*PA(40:5)*	*C43H75O8P*	$[M-H]^-$	749.51264	1.36
91	PE(P-38:4) / PE(O-38:5)	C43H78NO7P	$[M-H]^-$	750.54427	1.16
92	CerP(d44:3)	C44H84NO6P	$[M-H]^-$	752.59631	1.25
93	PS(34:2)	C40H74NO10P	$[M-H]^-$	758.49772	1.38
94	PS(34:1)	C40H76NO10P	$[M-H]^-$	760.51337	1.13
95	*PE(38:6)*	*C43H74NO8P*	$[M-H]^-$	762.50789	1.25
96	*PE(38:5)*	*C43H76NO8P*	$[M-H]^-$	764.52354	1.20
97	*PE(38:4)*	*C43H78NO8P*	$[M-H]^-$	766.53919	1.12
98	PG(36:5)	C42H73O10P	$[M-H]^-$	767.48682	1.66
99	PE(38:3)	C43H80NO8P	$[M-H]^-$	768.55484	1.83
100	PG(36:4)	C42H75O10P	$[M-H]^-$	769.50247	1.26

101	PG(36:3)	C42H77O10P	$[M-H]^-$	771.51812	1.23
102	PG(36:2)	C42H79O10P	$[M-H]^-$	773.53377	1.16
103	*PG(36:1)*	*C42H81O10P*	$[M-H]^-$	775.54942	1.43
104	PS(36:2)	C42H78NO10P	$[M-H]^-$	786.52902	1.14
105	PS(36:1)	C42H80NO10P	$[M-H]^-$	788.54467	0.96
106	PE(40:6)	C45H78NO8P	$[M-H]^-$	790.53919	1.33
107	*PE(40:5)*	*C45H80NO8P*	$[M-H]^-$	792.55484	1.70
108	PG(38:6)	C44H75O10P	$[M-H]^-$	793.50247	1.58
109	*PE(40:4)*	*C45H82NO8P*	$[M-H]^-$	794.57049	1.64
110	PG(38:5)	C44H77O10P	$[M-H]^-$	795.51812	2.07
111	PG(38:4)	C44H79O10P	$[M-H]^-$	797.53377	1.58
112	PS(38:4)	C44H78NO10P	$[M-H]^-$	810.52902	1.12
113	PE(42:7)	C47H80NO8P	$[M-H]^-$	816.55484	2.01
114	PG(40:7)	C46H77O10P	$[M-H]^-$	819.51812	2.05
115	PI(34:2)	C43H79O13P	$[M-H]^-$	833.51851	1.45
116	*PS(40:6)*	*C46H78NO10P*	$[M-H]^-$	834.52902	1.67
117	PI(34:1)	C43H81O13P	$[M-H]^-$	835.53416	1.56
118	*PS(40:4)*	*C46H82NO10P*	$[M-H]^-$	838.56032	1.21
119	PG(42:7)	C48H81O10P	$[M-H]^-$	847.54942	1.51
120	PI(36:5)	C45H77O13P	$[M-H]^-$	855.50286	1.68
121	PI(36:4)	C45H79O13P	$[M-H]^-$	857.51851	1.07
122	PI(36:3)	C45H81O13P	$[M-H]^-$	859.53416	1.51
123	PI(36:2)	C45H83O13P	$[M-H]^-$	861.54981	1.27
124	PG(44:8)	C50H83O10P	$[M-H]^-$	873.56507	1.41
125	PI(38:6)	C47H79O13P	$[M-H]^-$	881.51851	1.36
126	*PI(38:5)*	*C47H81O13P*	$[M-H]^-$	883.53416	1.16
127	*PI(38:4)*	*C47H83O13P*	$[M-H]^-$	885.54981	0.80
128	PI(40:6)	C49H83O13P	$[M-H]^-$	909.54981	1.72

129	*PI(40:5)*	*C49H85O13P*	$[M-H]^-$	911.56546	1.71
130	PI(40:4)	C49H87O13P	$[M-H]^-$	913.58111	1.78

Supplementary Table 2: List of lipid species annotated in positive-ion mode, corresponding molecular formula, ion adducts, theoretical *m/z* values and root mean square error (RMSE) values in parts per million (ppm). Lipid annotations are based on accurate *m/z* values (RMSE ≤ 2ppm) and diagnostic fragment ions from on-tissue fragmentation (MS/MS) experiments.

S. No	Lipid annotation	Molecular formula	$[M+H]^+$ (RMSE)	$[M+Na]^+$ (RMSE)	$[M+K]^+$ (RMSE)
1	CDP-Choline / Citicoline	C14H26N4O11P2	489.11457 (1.20)	511.09651 (2.09)	527.07045 (1.92)
2	Cer(d34:1)	C34H67NO3	538.51933 (1.89)	560.50128 (0.81)	576.47521 (0.70)
3	Cer(d36:1)	C36H71NO3			604.50651 (1.05)
4	Cer(d38:1)	C38H75NO3	594.58193 (1.00)		
5	Cer(d40:1)	C40H79NO3	622.61323 (0.91)		660.56911 (1.00)
6	Cer(d40:2)	C40H77NO3	620.59758 (0.86)		658.55346 (0.96)
7	Cer(d42:1)	C42H83NO3	650.64453 (1.09)		688.60042 (1.16)
8	Cer(d42:2)	C42H81NO3	648.62888 (0.83)	670.61083 (0.95)	686.58476 (0.88)
9	LacCer(d40:1)	C52H99NO13			984.67476 (1.72)
10	LPC(14:0)	C22H46NO7P	468.30843 (1.11)	490.29037 (1.25)	506.26431 (1.80)

11	LPC(16:0)	C24H50NO7P	496.33973 (0.72)	518.32167 (1.57)	534.29561 (1.56)
12	LPC(16:1)	C24H48NO7P	494.32408 (0.90)	516.30602 (1.61)	532.27996 (1.70)
13	LPC(18:0)	C26H54NO7P	524.37103 (1.94)	546.35297 (1.89)	562.32691 (0.58)
14	LPC(18:1)	C26H52NO7P	522.35538 (1.85)		560.31126 (0.47)
15	LPC(18:2)	C26H50NO7P	520.33973 (1.86)	542.32167 (2.08)	558.29561 (0.71)
16	LPC(20:4)	C28H50NO7P	544.33973 (2.02)	566.32167 (0.81)	582.29561 (0.63)
17	LPC(22:6)	C30H50NO7P	568.33973 (1.31)	590.32167 (0.90)	606.29561 (1.02)
18	*LPE(18:0)*	*C23H48NO7P*			520.27996 (1.76)
19	*LPE(18:1)*	*C23H46NO7P*			518.26431 (1.83)
20	*LPE(20:4)*	*C25H44NO7P*		524.27472 (1.83)	540.24866 (1.54)
21	*LPE(22:4)*	*C27H48NO7P*			568.27996 (1.04)
22	LPE(22:5)	C27H46NO7P			566.26431 (1.02)
23	*LPE(22:6)*	*C27H44NO7P*	526.29278 (1.62)	548.27472 (1.91)	564.24866 (1.19)
24	LPG(18:0)	C24H49O9P		535.30060 (1.44)	
25	*PA(32:1)*	*C35H67O8P*		669.44654 (0.86)	685.42048 (0.74)
26	*PA(32:2)*	*C35H65O8P*			683.40482 (1.21)
27	*PA(34:1)*	*C37H71O8P*		697.47784 (0.78)	713.45178 (0.53)

28	*PA(34:2)*	*C37H69O8P*		695.46219 (0.85)	711.43612 (0.59)
29	*PA(34:3)*	*C37H67O8P*		693.44654 (1.37)	709.42048 (1.68)
30	*PA(36:2)*	*C39H73O8P*			739.46743 (0.57)
31	*PA(36:3)*	*C39H71O8P*		721.47784 (0.89)	737.45178 (0.82)
32	*PA(38:2)*	*C41H77O8P*			767.49873 (1.09)
33	*PA(38:3)*	*C41H75O8P*		749.50914 (1.18)	765.48307 (1.05)
34	*PA(38:4)*	*C41H73O8P*			763.46743 (1.40)
35	*PA(38:5)*	*C41H71O8P*			761.45178 (1.06)
36	*PA(40:5)*	*C43H75O8P*			789.48307 (1.12)
37	PC(28:0)	C36H72NO8P	678.50679 (0.93)		716.46267 (0.90)
38	PC(30:0)	C38H76NO8P	706.53089 (0.42)	728.52004 (0.36)	744.49397 (0.30)
39	PC(30:1)	C38H74NO8P	704.52244 (0.75)	726.50349 (0.80)	742.47832 (0.75)
40	PC(32:0)	C40H80NO8P	734.56939 (0.54)	756.55134 (0.47)	772.52527 (0.34)
41	PC(32:1)	C40H78NO8P	732.55374 (0.32)	754.53569 (0.30)	770.50962 (0.31)
42	PC(32:2)	C40H76NO8P	730.53809 (1.72)	752.52004 (0.79)	768.49397 (0.74)
43	PC(34:1)	C42H82NO8P	760.58504 (0.39)	782.56699 (0.53)	798.54092 (0.33)
44	PC(34:2)	C42H80NO8P		780.55134 (0.79)	796.52527 (0.34)

45	PC(34:3)	C42H78NO8P		778.53569 (0.89)	794.50962 (0.87)
46	PC(34:4)	C42H76NO8P			792.49397 (1.20)
47	PC(36:1)	C44H86NO8P	788.61634 (1.11)		826.57222 (1.11)
48	PC(36:2)	C44H84NO8P	786.60069 (0.85)		824.55657 (0.48)
49	PC(36:3)	C44H82NO8P			822.54092 (1.82)
50	PC(36:4)	C44H80NO8P		804.55134 (0.52)	820.52527 (0.38)
51	PC(36:5)	C44H78NO8P		802.53569 (1.17)	818.50962 (1.19)
52	PC(38:3)	C46H86NO8P			850.57222 (1.72)
53	PC(38:4)	C46H84NO8P	810.60069 (1.56)	832.58264 (0.82)	848.55657 (0.70)
54	PC(38:5)	C46H82NO8P	808.58504 (1.89)	830.56699 (1.28)	
55	PC(38:6)	C46H80NO8P	806.56939 (1.59)	828.55134 (1.04)	844.52527 (0.90)
56	PC(38:7)	C46H78NO8P			842.50962 (1.57)
57	PC(40:4)	C48H88NO8P	838.63199 (1.81)	860.61394 (1.25)	876.58787 (1.17)
58	PC(40:5)	C48H86NO8P	836.61634 (1.75)	858.59829 (1.13)	874.57222 (1.19)
59	PC(40:6)	C48H84NO8P			872.55657 (1.32)
60	PC(40:7)	C48H82NO8P			870.54092 (1.51)
61	PC(O-34:1) / PC(P-34:0)	C42H84NO7P	746.60578 (0.81)	768.58772 (1.23)	

62	PC(O-36:4) / PC(P-36:3)	C44H82NO7P			790.57207 (1.48)
63	PC(P-32:0) / PC(O-32:1)	C40H80NO7P	718.57448 (0.86)	740.55642 (0.93)	
64	PC(P-38:4) / PC(O-38:5)	C46H84NO7P	794.60578 (1.24)	816.58772 (1.22)	832.56166 (1.65)
65	PE(30:1)	C35H68NO8P			700.43137 (1.29)
66	PE(30:2)	C35H66NO8P			698.41572 (1.09)
67	*PE(32:0)*	*C37H74NO8P*	692.52244 (0.93)		730.47832 (1.07)
68	*PE(32:1)*	*C37H72NO8P*	690.50679 (0.86)	712.48874 (1.77)	728.46267 (1.09)
69	*PE(34:0)*	*C39H78NO8P*	720.55374 (1.01)	742.53569 (1.67)	758.50962 (1.35)
70	*PE(34:1)*	*C39H76NO8P*	718.53809 (0.72)		756.49397 (1.15)
71	*PE(34:2)*	*C39H74NO8P*	716.52244 (0.85)	738.50439 (1.61)	754.47832 (1.10)
72	PE(36:0)	C41H82NO8P	748.58504 (1.08)		
73	*PE(36:1)*	*C41H80NO8P*	746.56939 (0.81)		784.52527 (0.99)
74	*PE(36:2)*	*C41H78NO8P*			782.50962 (0.83)
75	*PE(36:3)*	*C41H76NO8P*			780.49397 (1.36)
76	*PE(36:4)*	*C41H74NO8P*		762.50439 (1.76)	778.47832 (1.60)
77	PE(38:1)	C43H84NO8P	774.60069 (0.80)		
78	PE(38:2)	C43H82NO8P	772.58504 (1.12)		

79	*PE(38:4)*	*C43H78NO8P*	768.55374 (1.73)	790.53569 (1.02)	806.50962 (0.87)
80	*PE(38:5)*	*C43H76NO8P*	766.53809 (1.75)	788.52004 (0.98)	804.49397 (1.24)
81	*PE(38:6)*	*C43H74NO8P*	764.52244 (1.57)	786.50439 (1.04)	802.47832 (1.33)
82	*PE(40:4)*	*C45H82NO8P*	796.58504 (1.67)		834.54092 (1.11)
83	*PE(40:5)*	*C45H80NO8P*	794.56939 (1.70)		832.52527 (1.20)
84	PE(O-36:5) / PE(P-36:4)	C41H74NO7P	724.52753 (0.95)		762.48341 (0.94)
85	PE(O-38:5) / PE(P-38:4)	C43H78NO7P	752.55883 (0.93)		790.51471 (1.26)
86	PE(O-38:6) / PE(P-38:5)	C43H76NO7P	750.54318 (1.04)		
87	*PE(P-34:1) / PE(O-34:2)*	*C39H76NO7P*	702.54318 (1.04)		
88	PE(P-38:6)	C43H74NO7P	748.52753 (1.13)		
89	*PG(34:1)*	*C40H77O10P*			787.48855 (1.30)
90	*PG(36:1)*	*C42H81O10P*		799.54592 (2.04)	
91	PG(38:1)	C44H85O10P		827.57722 (1.80)	
92	PG(40:4)	C46H83O10P			849.56157 (1.83)
93	*PI(38:4)*	*C47H83O13P*		909.54631 (1.42)	925.52025 (1.13)
94	*PI(38:5)*	*C47H81O13P*		907.53066 (1.40)	923.50460 (1.20)
95	*PI(40:5)*	*C49H85O13P*		935.56196 (1.82)	951.53590 (1.89)

96	PI-Cer(t38:0)	C44H88NO12P			892.56753 (1.63)
97	PIP(36:2)	C45H84O16P2		965.51264 (1.45)	
98	PIP(38:3)	C47H86O16P2		991.52829 (1.79)	
99	PS(38:3)	C44H80NO10P			852.51510 (1.51)
100	PS(40:3)	C46H84NO10P			880.54640 (1.59)
101	*PS(40:4)*	*C46H82NO10P*	840.57499 (1.54)		878.53075 (1.27)
102	PS(40:5)	C46H80NO10P	838.55922 (1.64)		876.51510 (1.64)
103	*PS(40:6)*	*C46H78NO10P*		858.52552 (1.69)	874.49945 (1.78)
104	PS(44:10)	C50H78NO10P		906.52552 (1.52)	
105	PS(44:9)	C50H80NO10P		908.54117 (1.50)	
106	SHexCer(t33:1)	C39H75NO12S			820.46415 (2.08)
107	SM(d34:1)	C39H79N2O6P	703.57481 (0.46)	725.55676 (0.37)	741.53069 (0.36)
108	SM(d34:2)	C39H77N2O6P	701.55916 (0.88)	723.54111 (0.78)	739.51504 (0.95)
109	SM(d40:1)	C45H91N2O6P	787.66871 (1.08)		825.62459 (0.95)
110	SM(d40:2)	C45H89N2O6P	785.65306 (1.52)	807.63501 (0.96)	823.60894 (1.00)
111	SM(d42:1)	C47H95N2O6P	815.70001 (1.28)		853.65589 (1.44)
112	SM(d42:2)	C47H93N2O6P	813.68436 (0.80)		851.64024 (0.66)

113	SM(d42:3)	C47H91N2O6P	811.66871 (0.94)	833.65066 (1.02)	849.62459 (0.77)
114	TG(48:0)	C51H98O6			845.69946 (1.28)
115	TG(48:1)	C51H96O6			843.68381 (1.08)
116	TG(50:1)	C53H100O6		855.74117 (0.96)	871.71511 (0.85)
117	TG(50:2)	C53H98O6		853.72552 (1.09)	869.69946 (0.96)
118	TG(52:1)	C55H104O6			899.74641 (1.24)
119	TG(52:2)	C55H102O6		881.75682 (1.10)	897.73076 (1.00)
120	TG(52:3)	C55H100O6		879.74117 (1.03)	895.71511 (1.05)
121	TG(54:2)	C57H106O6			925.76206 (1.40)
122	TG(54:4)	C57H102O6			921.73076 (1.25)
123	TG(56:5)	C59H104O6			947.74641 (1.50)
124	TG(56:6)	C59H102O6			945.73076 (1.43)

Supplementary Table 3: On-tissue fragmentation (MS/MS) of annotated lipid species, precursor *m/z* values, ion adducts, corresponding diagnostic fragment ions (*m/z*) in negative- and positive-ion mode.

S. No	Precursor *m/z* (observed)	Diagnostic fragment ions (*m/z*)	Lipid annotation	Ion adduct
1	721.5039	152.9946, 171.0054, 255.2330, 391.2260	PG(32:0)	$[M-H]^-$

2	719.4883	152.9946, 253.2174, 255.2330, 391.2261	PG(32:1)	$[M-H]^-$
3	745.5041	152.9946, 253.2174, 255.2330, 279.2332, 281.2489	PG(34:2)	$[M-H]^-$
4	693.4725	152.9946, 227.2014, 255.2331	PG(30:0)	$[M-H]^-$
5	747.5197	152.9946, 255.2331, 281.2488, 391.2262	PG(34:1)	$[M-H]^-$
6	769.5035	152.9946, 279.2331, 255.2330, 303.2333	PG(36:4)	$[M-H]^-$
7	771.5189	152.9946, 279.2332, 281.2489, 255.2332, 305.2491	PG(36:3)	$[M-H]^-$
8	773.5348	152.9946, 281.2489	PG(36:2)	$[M-H]^-$
9	795.5194	152.9946, 255.2330, 281.2489, 303.2333, 329.2491	PG(38:5)	$[M-H]^-$
10	797.5347	152.9946, 255.2330, 331.2647, 445.2343, 764.8609, 303.2335	PG(38:4)	$[M-H]^-$
11	793.5035	152.9946, 255.2331, 327.2332, 764.9478	PG(38:6)	$[M-H]^-$
12	509.2885	152.9945, 281.2487	LPG(18:1)	$[M-H]^-$
13	647.4673	152.9946, 255.2331, 391.2262, 409.2367	PA(32:0)	$[M-H]^-$
14	673.4828	152.9946, 255.2331, 281.2489, 391.2262	PA(34:1)	$[M-H]^-$
15	671.4674	152.9946, 253.2174, 255.2330, 279.2333, 281.2489	PA(34:2)	$[M-H]^-$
16	645.4513	152.9946, 253.2174, 255.2331, 227.2013, 281.2491, 391.2262	PA(32:1)	$[M-H]^-$
17	699.4974	281.2484	PA(36:2)	$[M-H]^-$
18	716.5235	281.2482, 255.2325, 188.3342	PE(34:1)	$[M-H]^-$
19	788.5441	761.6053, 701.5124	PS(36:1)	$[M-H]^-$
20	524.2994	152.9946, 437.2678	LPS(18:0)	$[M-H]^-$
21	489.1140	378.0706, 360.0601, 264.0394,	CDP-	$[M+H]^+$

		184.0733	Choline	
22	511.0965	452.0219, 400.0524, 328.0299, 226.9480, 206.0552	CDP-Choline	$[M+Na]^+$
23	527.0715	467.9964, 356.9530, 344.0038, 283.9845, 242.9219, 222.0291	CDP-Choline	$[M+K]^+$
24	496.3401	104.1076, 184.0736, 478.3294	LPC(16:0)	$[M+H]^+$
25	518.3225	104.1076, 459.2480, 146.9821, 313.2737	LPC(16:0)	$[M+Na]^+$
26	534.2965	104.1076, 475.2224	LPC(16:0)	$[M+K]^+$
27	494.3245	104.1076, 184.0737, 208.0126, 222.0295	LPC(16:1)	$[M+H]^+$
28	516.3067	457.2323, 146.9819, 311.2581	LPC(16:1)	$[M+Na]^+$
29	706.5380	184.0736	PC(30:0)	$[M+H]^+$
30	728.5199	146.9820, 523.4729, 545.4548, 669.4464	PC(30:0)	$[M+Na]^+$
31	744.4941	162.9560, 685.4204	PC(30:0)	$[M+K]^+$
32	730.5386	184.0737	PC(32:2)	$[M+H]^+$
33	752.5199	693.4465, 569.4539, 146.9821	PC(32:2)	$[M+Na]^+$
34	732.5536	184.0737	PC(32:1)	$[M+H]^+$
35	754.5359	695.4623, 571.4697, 549.4883, 146.9820	PC(32:1)	$[M+Na]^+$
36	770.5097	162.9560, 711.4360	PC(32:1)	$[M+K]^+$
37	734.5695	184.0736	PC(32:0)	$[M+H]^+$
38	756.5516	146.9820, 551.5042, 573.4854, 697.4781	PC(32:0)	$[M+Na]^+$
39	772.5255	162.9559, 713.4520	PC(32:0)	$[M+K]^+$
40	468.3090	184.0738	LPC(14:0)	$[M+H]^+$
41	520.3409	184.0738, 104.14076	LPC(18:2)	$[M+H]^+$
42	542.3230	146.9822, 483.2486	LPC(18:2)	$[M+Na]^+$
43	558.2963	499.2226	LPC(18:2)	$[M+K]^+$

44	522.3565	184.0738, 504.3452, 104.1077	LPC(18:1)	$[M+H]^+$
45	560.3119	501.2384, 162.9561	LPC(18:1)	$[M+K]^+$
46	544.3406**	485.2645, 146.9822	LPC(18:1)	$[M+Na]^+$
47	544.3406**	184.0738, 526.3308,104.1077	LPC(20:4)	$[M+H]^+$
48	746.6037	184.0732	PC(O-34:1) / PC(P-34:0)	$[M+H]^+$
49	703.5734	184.0737	SM(34:1)	$[M+H]^+$
50	725.5555	666.4821, 542.4898, 146.9824	SM(34:1)	$[M+Na]^+$
51	741.5291	682.4558, 558.4638, 162.9563	SM(34:1)	$[M+K]^+$

References for fragment ions (*m/z*) confirmation

1. Wolf S, Schmidt S, Müller-Hannemann M, Neumann S. In silico fragmentation for computer assisted identification of metabolite mass spectra. BMC bioinformatics. 2010 Mar 22;11(1):1)
2. Murphy, R. C. Tandem Mass Spectrometry of Lipids: Molecular analysis of complex lipids. (Royal Society of Chemistry, 2014).

**MS/MS in single spectrum (due to narrow *m/z*, on-tissue MS/MS)

Supplementary Data (MS/MS spectra)

Validation of lipid annotations by on-tissue fragmentation (MS/MS) in negative- and positive-ion mode

[PG(32:0)-H]$^-$

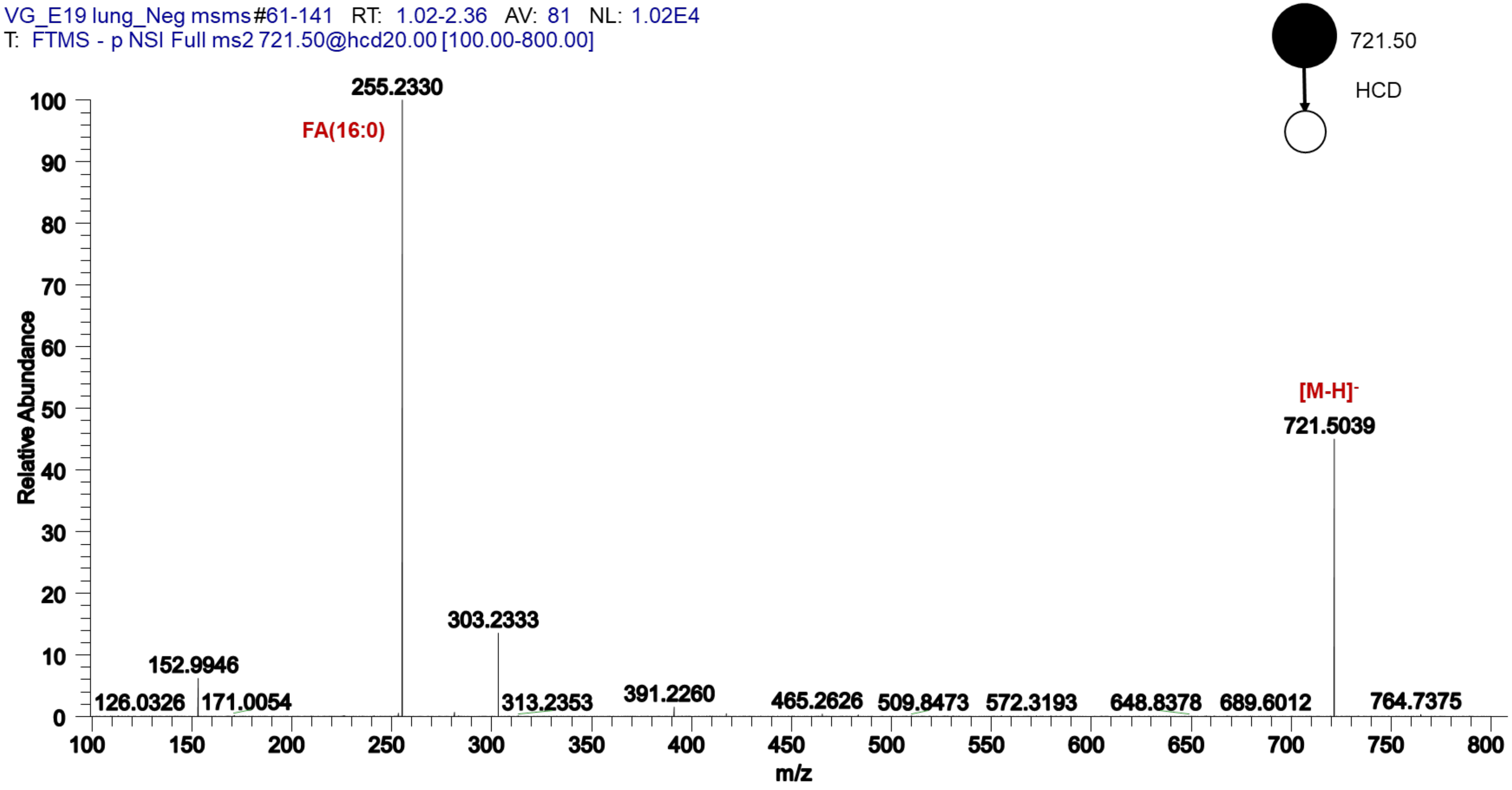
VG_E19 lung_Neg msms#61-141 RT: 1.02-2.36 AV: 81 NL: 1.02E4
T: FTMS - p NSI Full ms2 721.50@hcd20.00 [100.00-800.00]
721.50
HCD
255.2330
FA(16:0)
[M-H]-
721.5039
303.2333
152.9946
126.0326
171.0054
313.2353
391.2260
465.2626
509.8473
572.3193
648.8378
689.6012
764.7375
Relative Abundance
m/z

[PG(32:1)-H]⁻

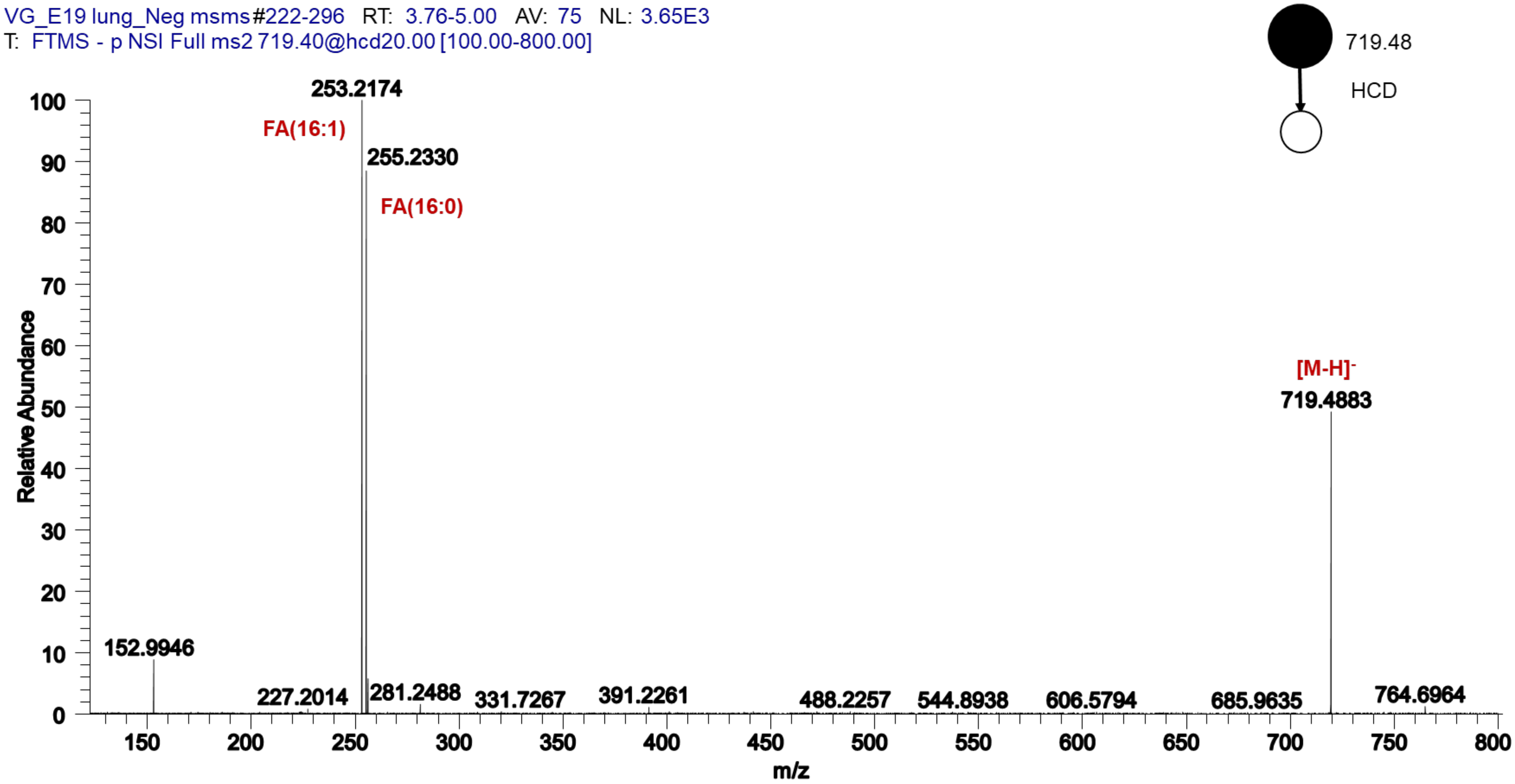

VG_E19 lung_Neg msms#222-296 RT: 3.76-5.00 AV: 75 NL: 3.65E3
T: FTMS - p NSI Full ms2 719.40@hcd20.00 [100.00-800.00]
719.48
HCD
253.2174
FA(16:1)
255.2330
FA(16:0)
[M-H]-
719.4883
152.9946
227.2014
281.2488
331.7267
391.2261
488.2257
544.8938
606.5794
685.9635
764.6964
Relative Abundance
m/z

[PG(34:2)-H]$^-$

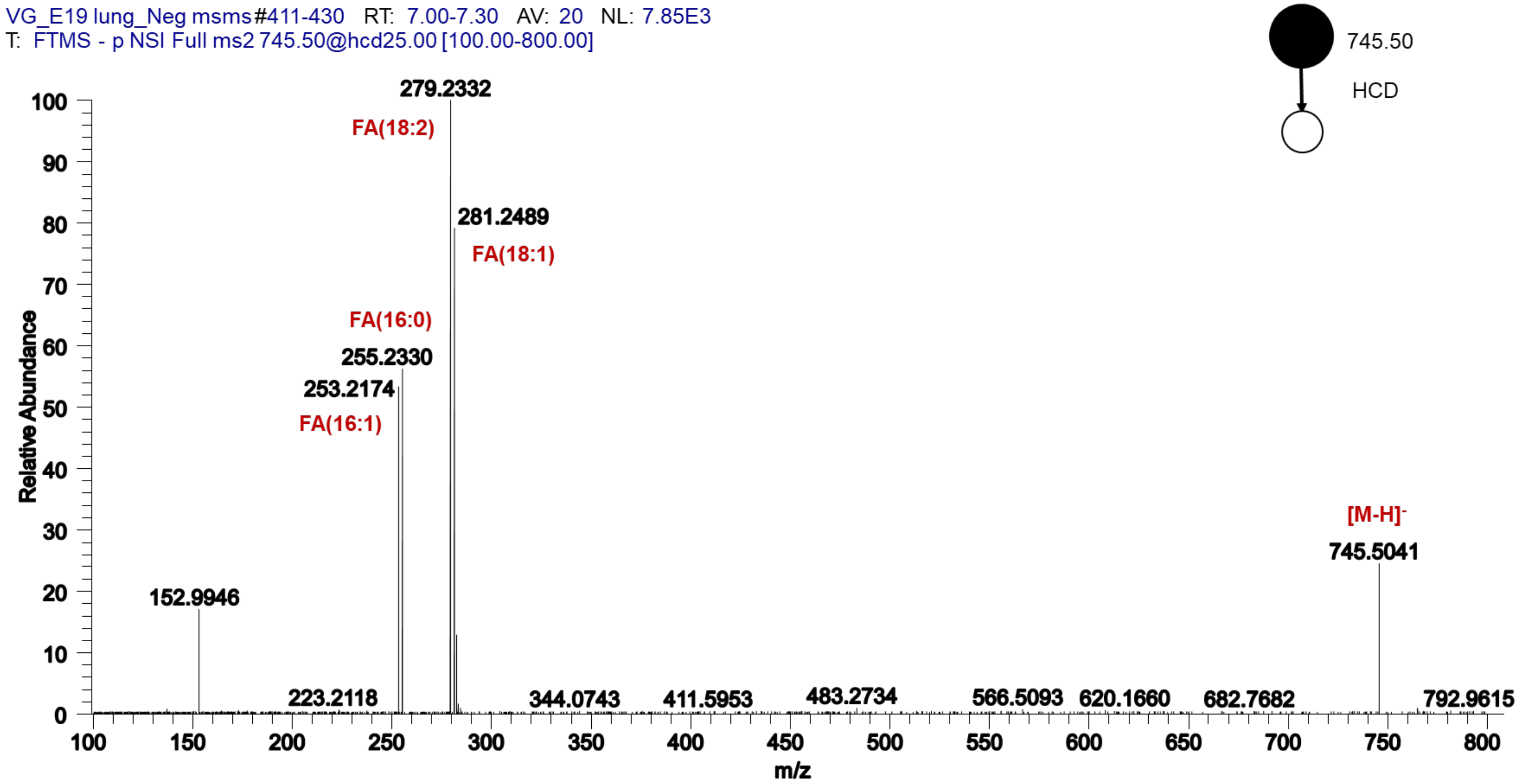
VG_E19 lung_Neg msms#411-430 RT: 7.00-7.30 AV: 20 NL: 7.85E3
T: FTMS - p NSI Full ms2 745.50@hcd25.00 [100.00-800.00]
745.50
HCD
279.2332
FA(18:2)
281.2489
FA(18:1)
FA(16:0)
255.2330
253.2174
FA(16:1)
152.9946
223.2118
344.0743
411.5953
483.2734
566.5093
620.1660
682.7682
[M-H]-
745.5041
792.9615
Relative Abundance
m/z
100
90
80
70
60
50
40
30
20
10
0
100
150
200
250
300
350
400
450
500
550
600
650
700
750
800

[PG(30:0)-H]$^-$

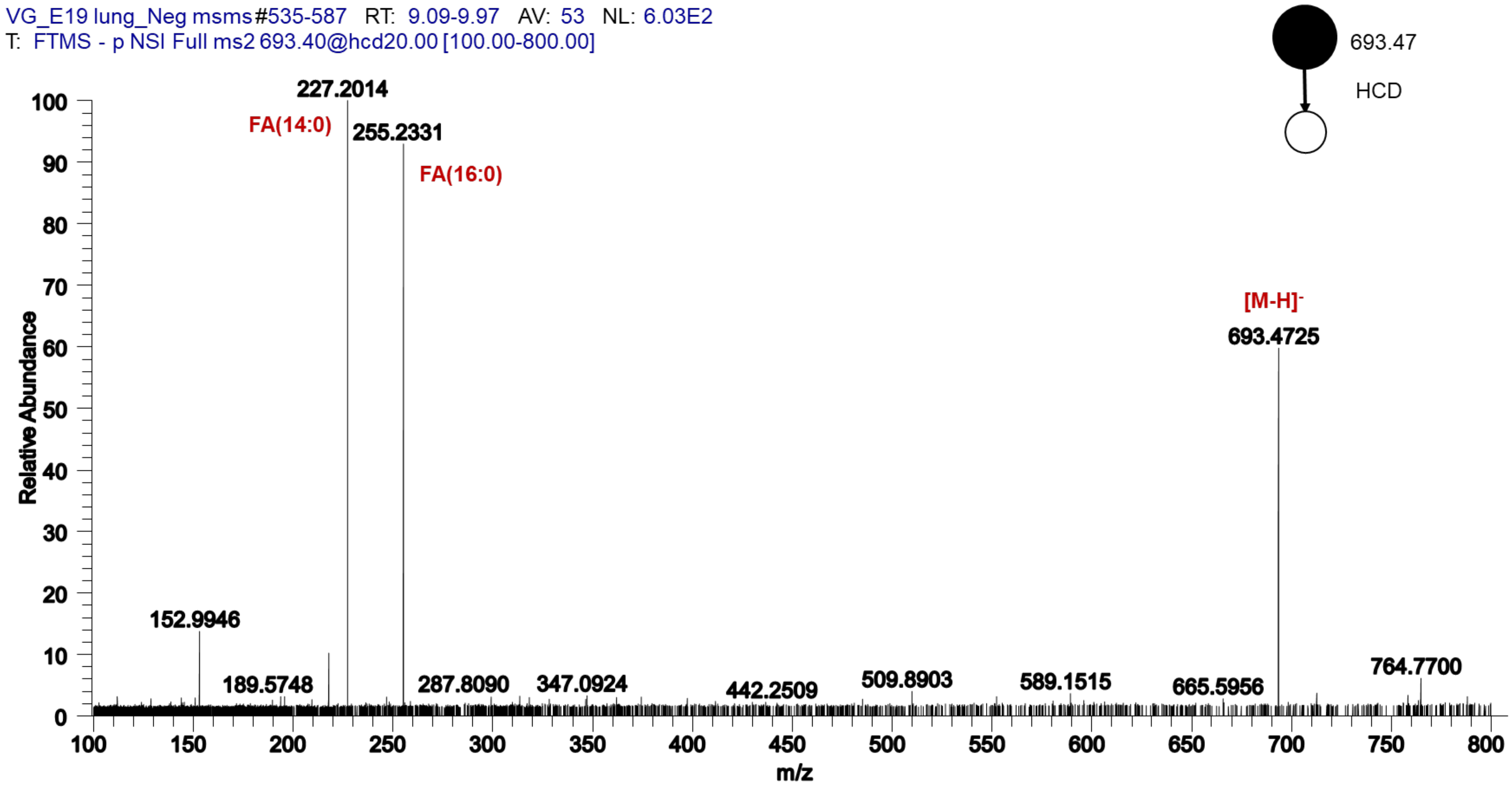

VG_E19 lung_Neg msms#535-587 RT: 9.09-9.97 AV: 53 NL: 6.03E2
T: FTMS - p NSI Full ms2 693.40@hcd20.00 [100.00-800.00]
693.47
HCD
227.2014
FA(14:0)
255.2331
FA(16:0)
[M-H]-
693.4725
152.9946
189.5748
287.8090
347.0924
442.2509
509.8903
589.1515
665.5956
764.7700
Relative Abundance
m/z

[PG(34:1)-H]$^-$

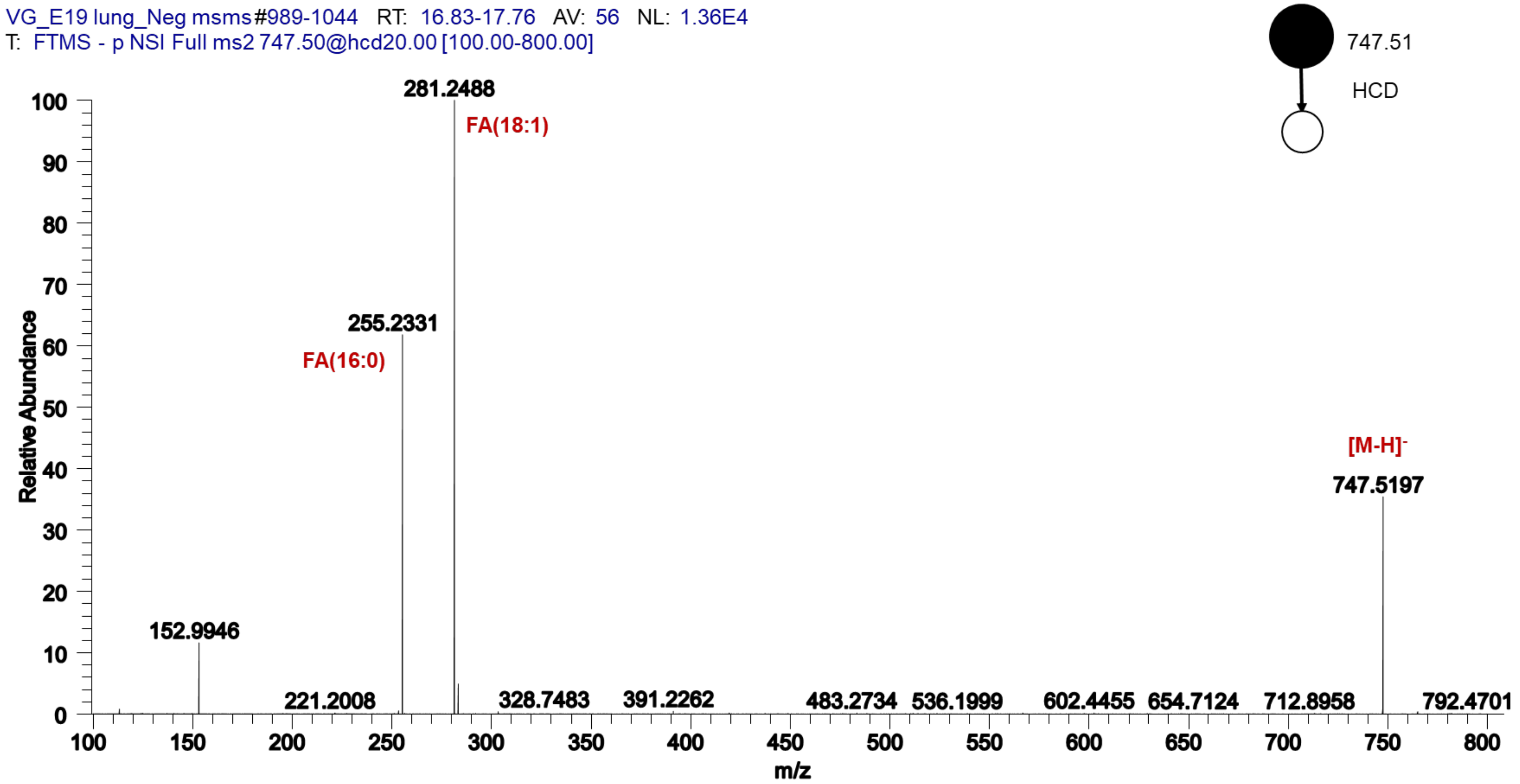

VG_E19 lung_Neg msms#989-1044 RT: 16.83-17.76 AV: 56 NL: 1.36E4
T: FTMS - p NSI Full ms2 747.50@hcd20.00 [100.00-800.00]
747.51
HCD
281.2488
FA(18:1)
255.2331
FA(16:0)
[M-H]-
747.5197
152.9946
221.2008
328.7483
391.2262
483.2734
536.1999
602.4455
654.7124
712.8958
792.4701
Relative Abundance
m/z

[PG(36:4)-H]$^-$

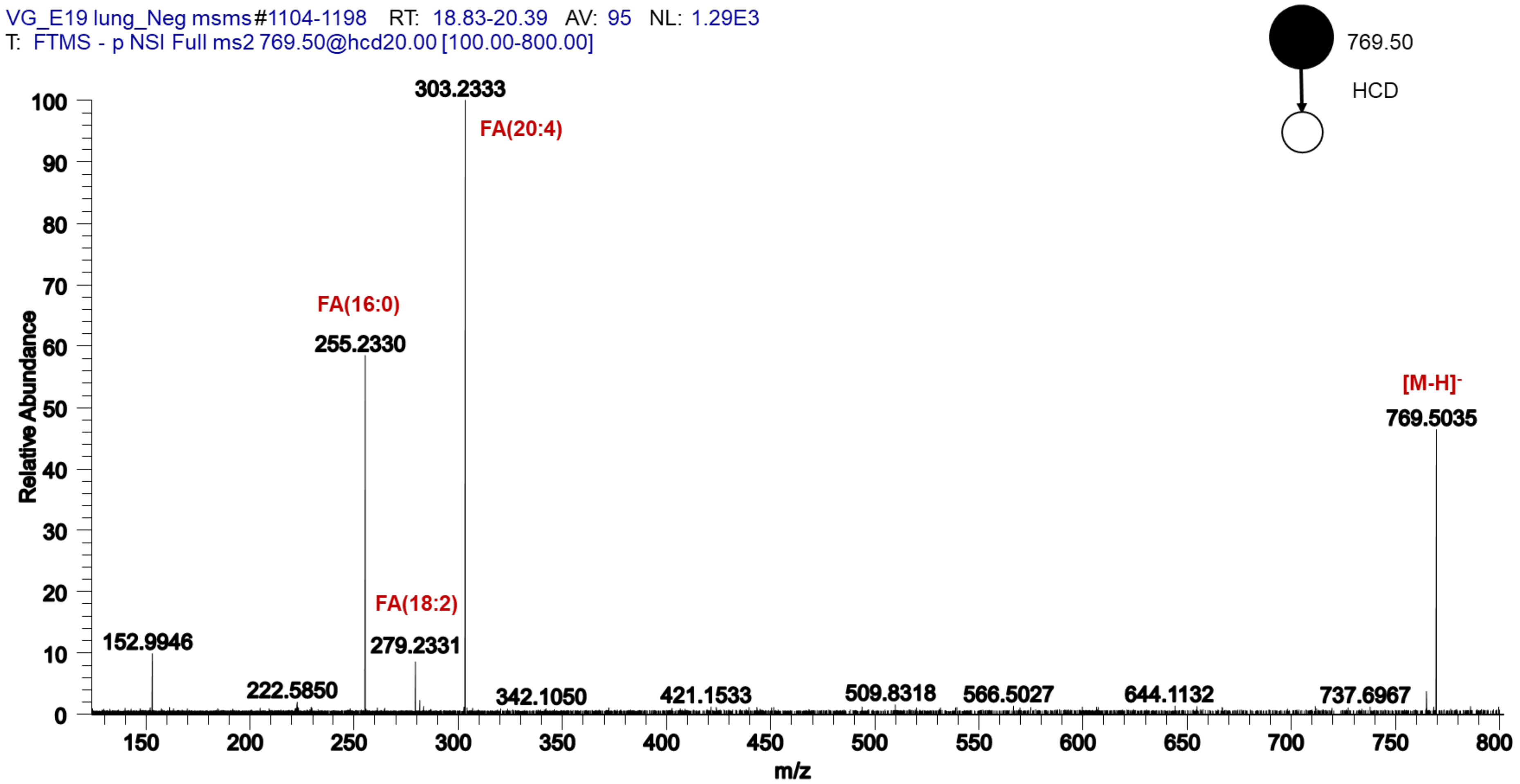
VG_E19 lung_Neg msms#1104-1198 RT: 18.83-20.39 AV: 95 NL: 1.29E3
T: FTMS - p NSI Full ms2 769.50@hcd20.00 [100.00-800.00]
769.50
HCD
303.2333
FA(20:4)
FA(16:0)
255.2330
[M-H]-
769.5035
FA(18:2)
279.2331
152.9946
222.5850
342.1050
421.1533
509.8318
566.5027
644.1132
737.6967
Relative Abundance
m/z

[PG(36:3)-H]⁻

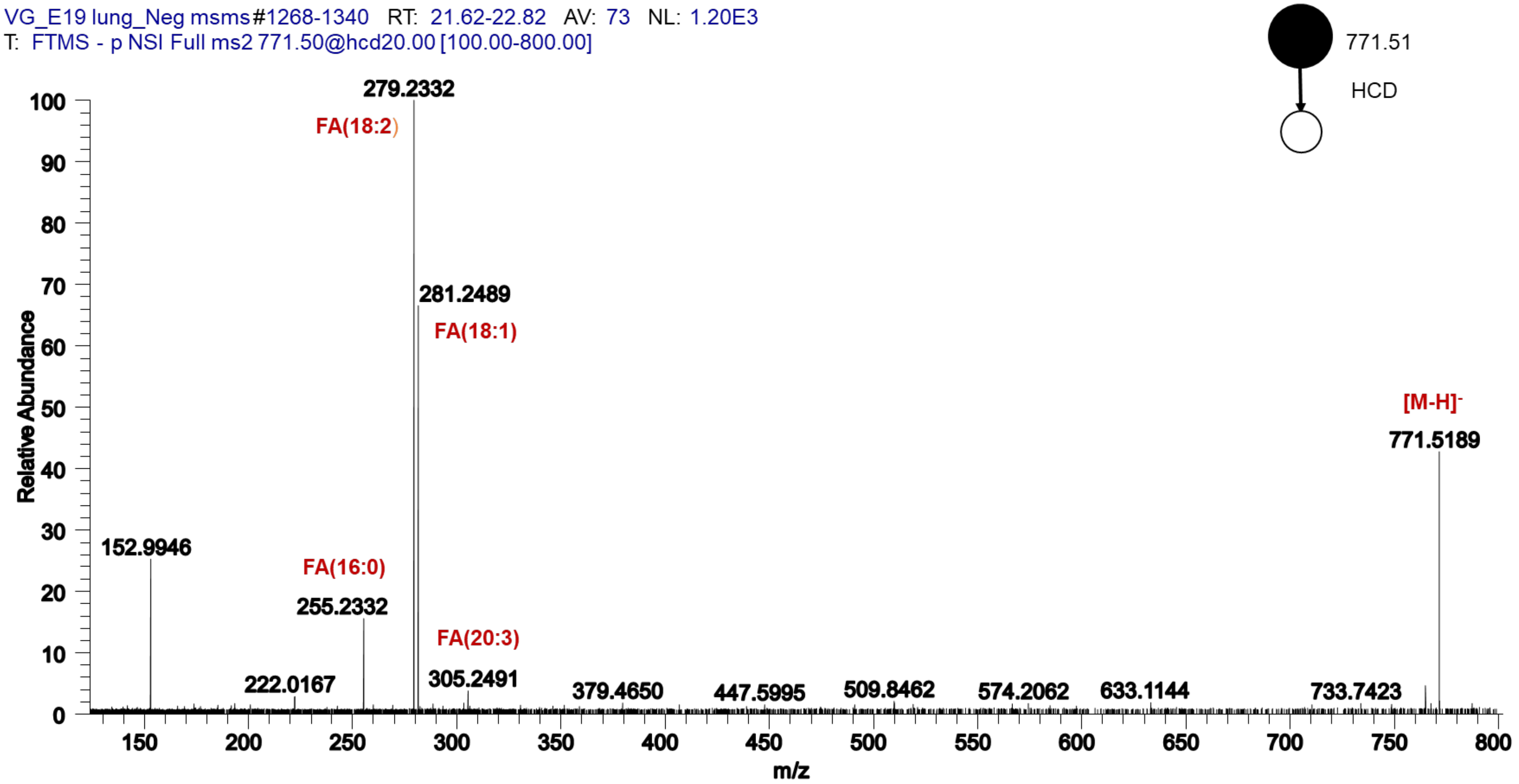

VG_E19 lung_Neg msms#1268-1340 RT: 21.62-22.82 AV: 73 NL: 1.20E3
T: FTMS - p NSI Full ms2 771.50@hcd20.00 [100.00-800.00]
771.51
HCD
Relative Abundance
m/z
279.2332
FA(18:2)
281.2489
FA(18:1)
[M-H]-
771.5189
152.9946
FA(16:0)
255.2332
FA(20:3)
222.0167
305.2491
379.4650
447.5995
509.8462
574.2062
633.1144
733.7423

[PG(36:2)-H]$^-$

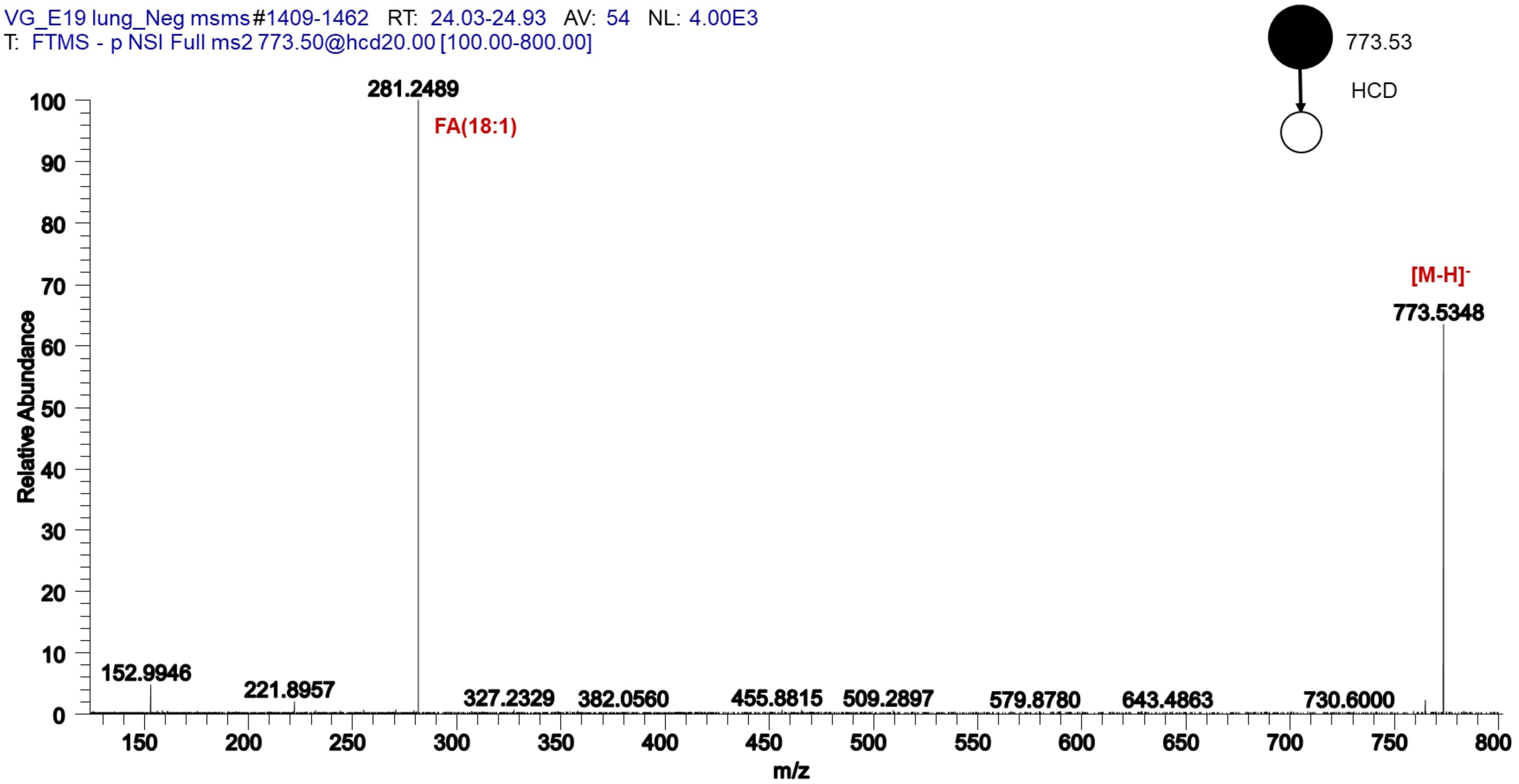
VG_E19 lung_Neg msms#1409-1462 RT: 24.03-24.93 AV: 54 NL: 4.00E3
T: FTMS - p NSI Full ms2 773.50@hcd20.00 [100.00-800.00]
773.53
HCD
281.2489
FA(18:1)
[M-H]-
773.5348
152.9946
221.8957
327.2329
382.0560
455.8815
509.2897
579.8780
643.4863
730.6000
Relative Abundance
m/z

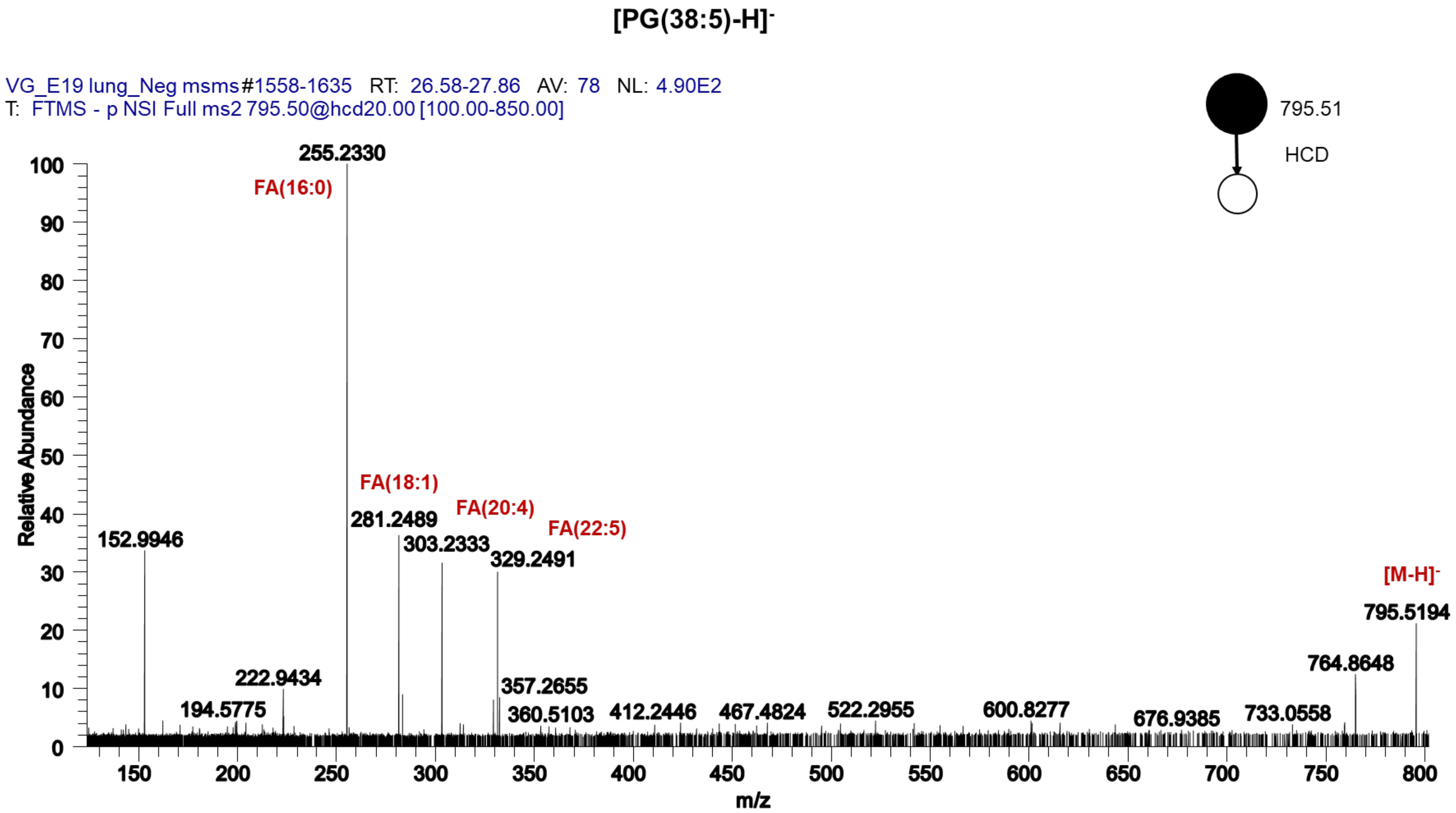

[PG(38:5)-H]-
VG_E19 lung_Neg msms#1558-1635 RT: 26.58-27.86 AV: 78 NL: 4.90E2
T: FTMS - p NSI Full ms2 795.50@hcd20.00 [100.00-850.00]
795.51
HCD
255.2330
FA(16:0)
FA(18:1)
281.2489
FA(20:4)
303.2333
FA(22:5)
329.2491
152.9946
222.9434
194.5775
357.2655
360.5103
412.2446
467.4824
522.2955
600.8277
676.9385
733.0558
764.8648
[M-H]-
795.5194
Relative Abundance
m/z

[PG(38:4)-H]⁻

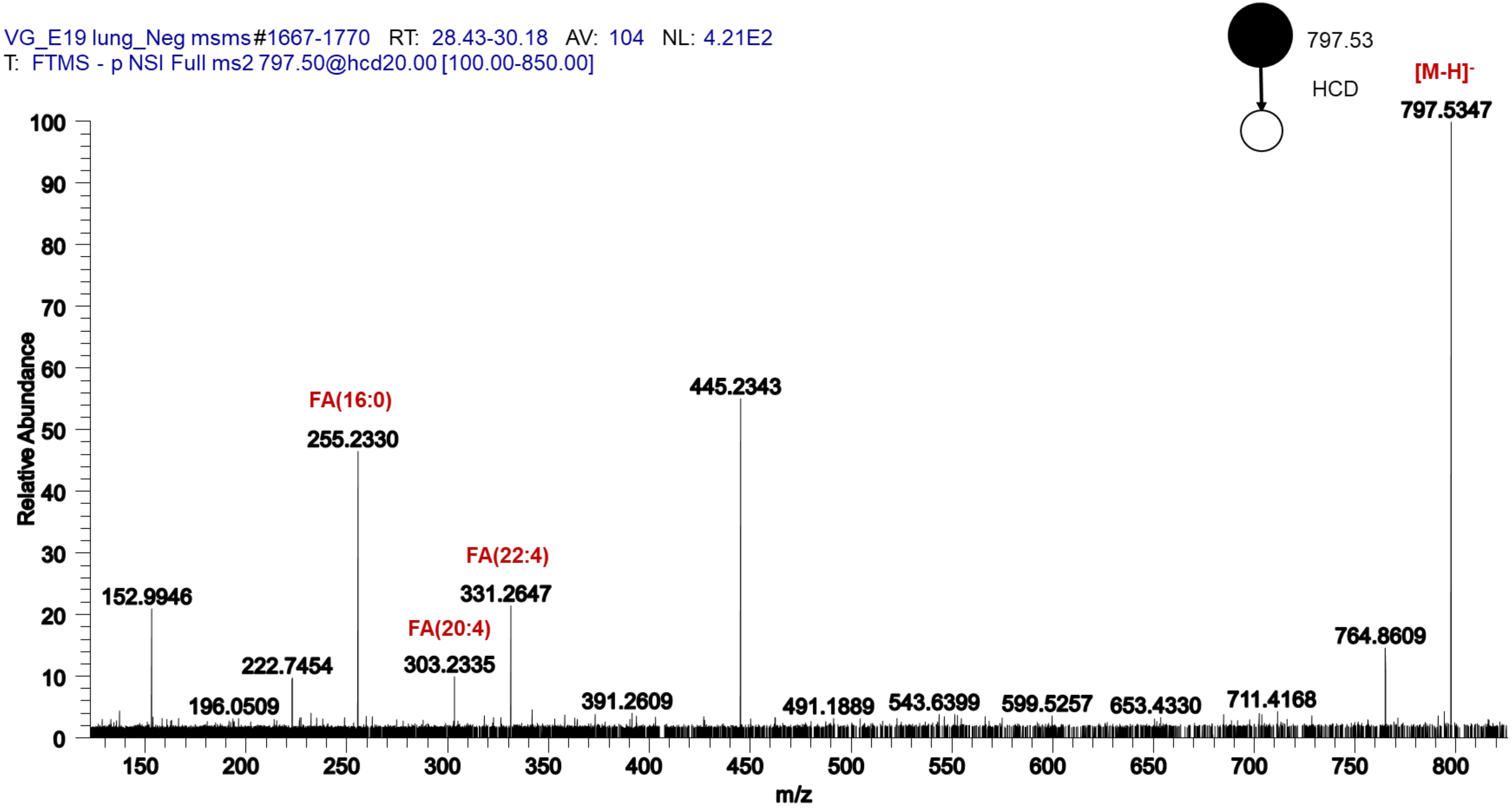
VG_E19 lung_Neg msms#1667-1770 RT: 28.43-30.18 AV: 104 NL: 4.21E2
T: FTMS - p NSI Full ms2 797.50@hcd20.00 [100.00-850.00]
797.53
HCD
[M-H]-
797.5347
445.2343
FA(16:0)
255.2330
FA(22:4)
331.2647
152.9946
FA(20:4)
303.2335
222.7454
196.0509
391.2609
491.1889
543.6399
599.5257
653.4330
711.4168
764.8609
Relative Abundance
m/z

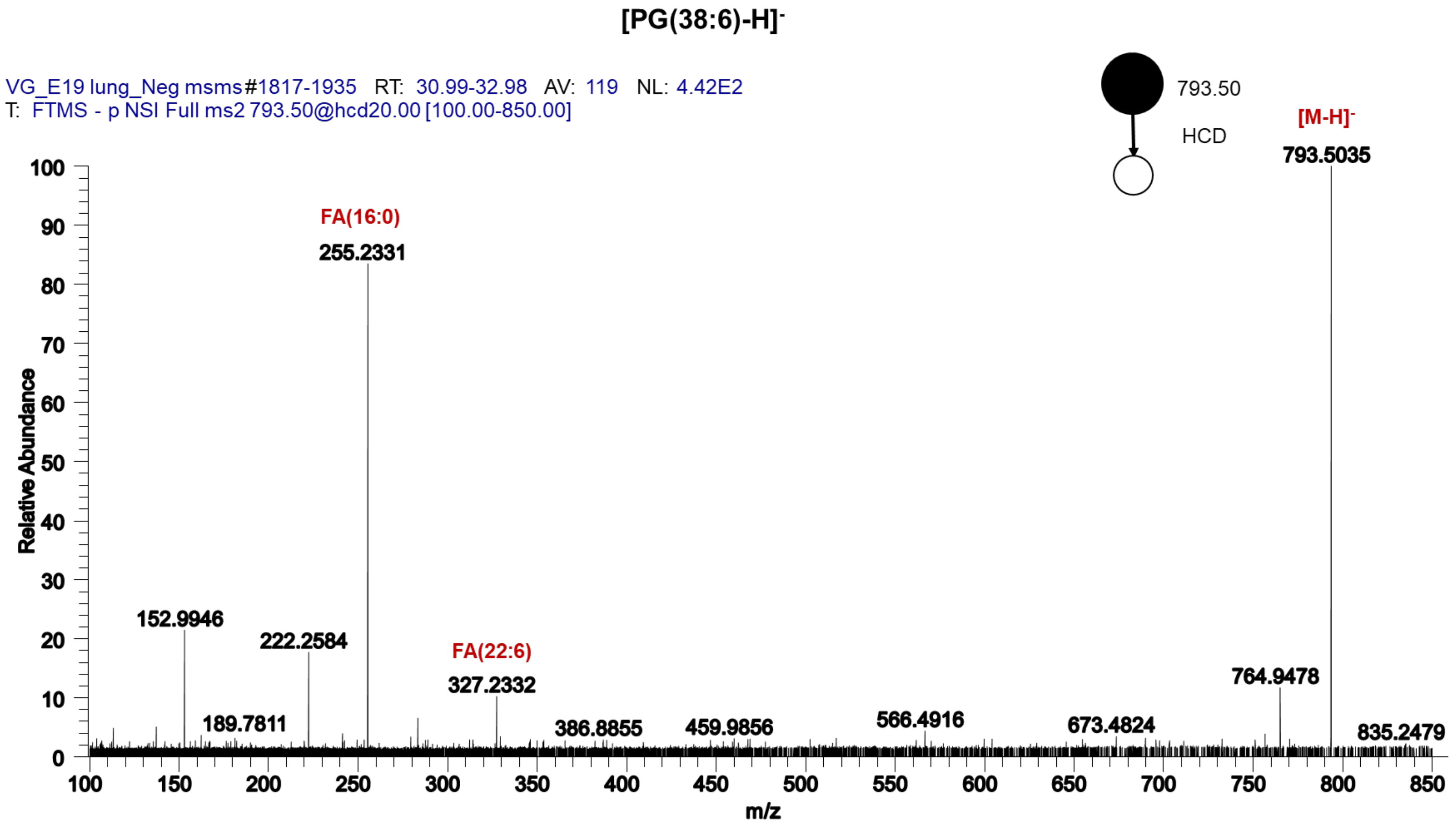

[PG(38:6)-H]-
VG_E19 lung_Neg msms#1817-1935 RT: 30.99-32.98 AV: 119 NL: 4.42E2
T: FTMS - p NSI Full ms2 793.50@hcd20.00 [100.00-850.00]
793.50
HCD
[M-H]-
793.5035
FA(16:0)
255.2331
152.9946
222.2584
189.7811
FA(22:6)
327.2332
386.8855
459.9856
566.4916
673.4824
764.9478
835.2479
Relative Abundance
m/z

[LPG(18:1)-H]$^-$

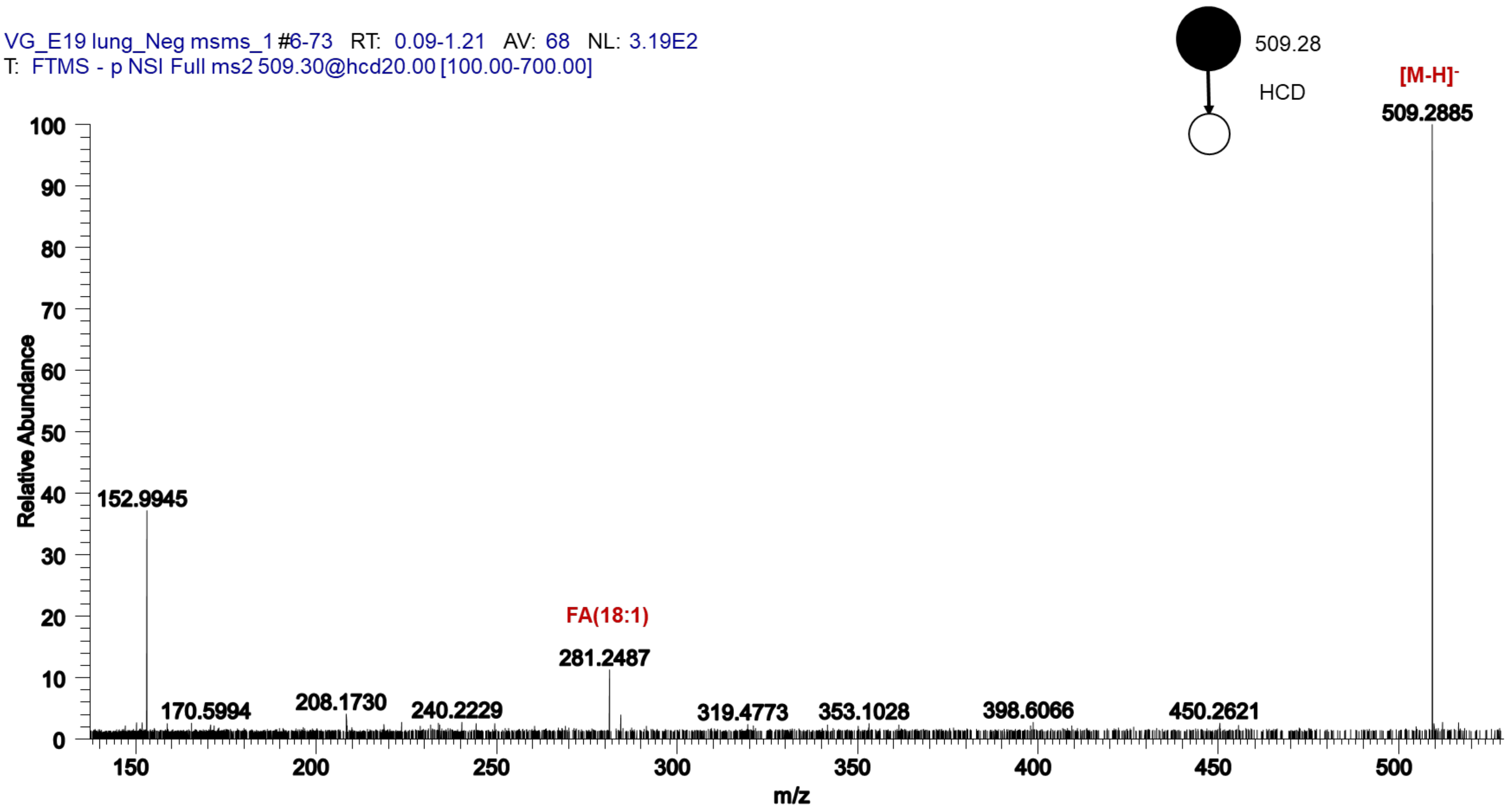

VG_E19 lung_Neg msms_1 #6-73 RT: 0.09-1.21 AV: 68 NL: 3.19E2
T: FTMS - p NSI Full ms2 509.30@hcd20.00 [100.00-700.00]
509.28
HCD
[M-H]-
509.2885
152.9945
FA(18:1)
281.2487
170.5994
208.1730
240.2229
319.4773
353.1028
398.6066
450.2621
Relative Abundance
m/z

[PA(32:0)-H]$^-$

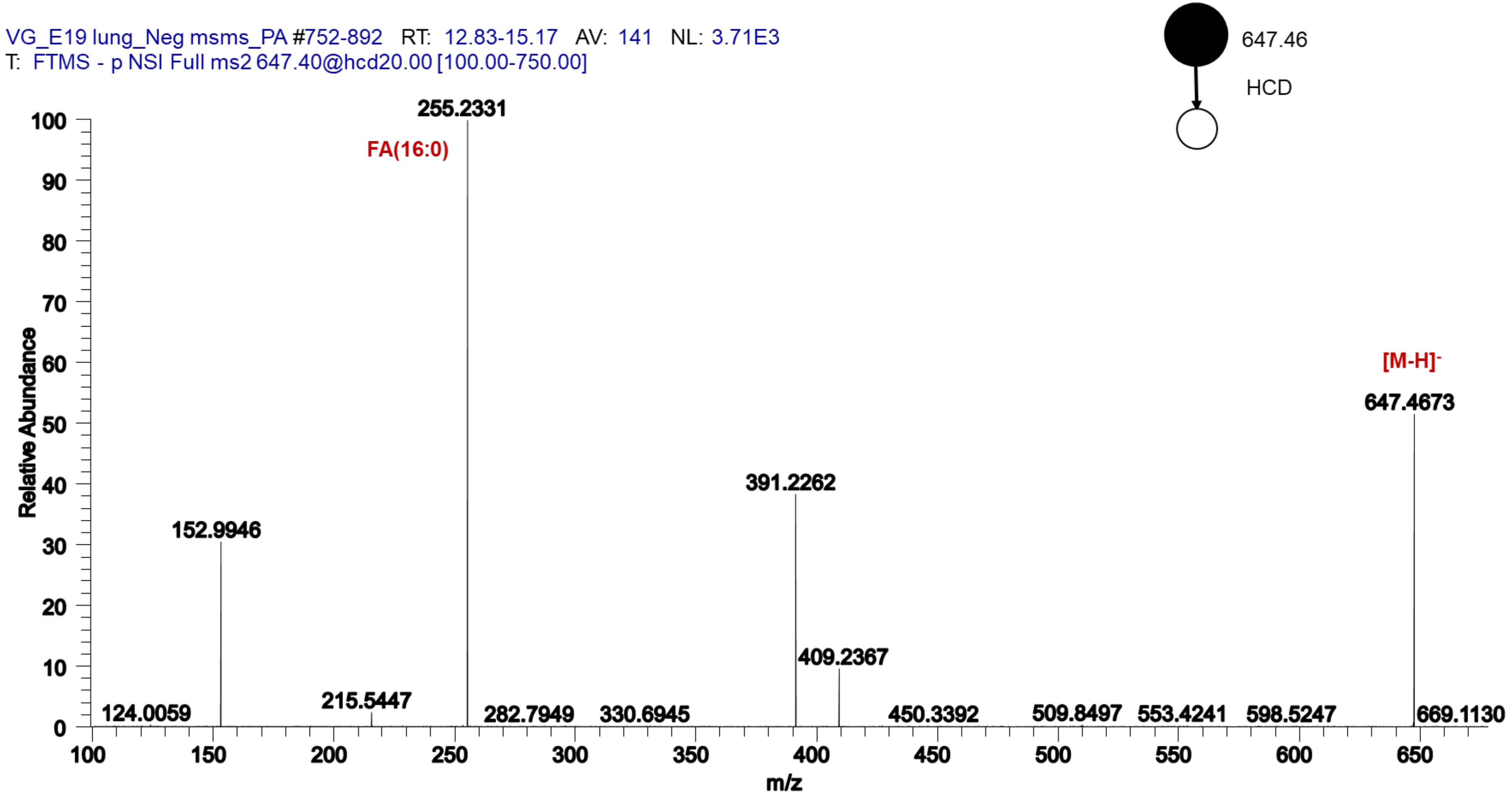
VG_E19 lung_Neg msms_PA #752-892 RT: 12.83-15.17 AV: 141 NL: 3.71E3
T: FTMS - p NSI Full ms2 647.40@hcd20.00 [100.00-750.00]
647.46
HCD
255.2331
FA(16:0)
[M-H]-
647.4673
391.2262
152.9946
409.2367
124.0059
215.5447
282.7949
330.6945
450.3392
509.8497
553.4241
598.5247
669.1130
Relative Abundance
m/z

[PA(34:1)-H]$^-$

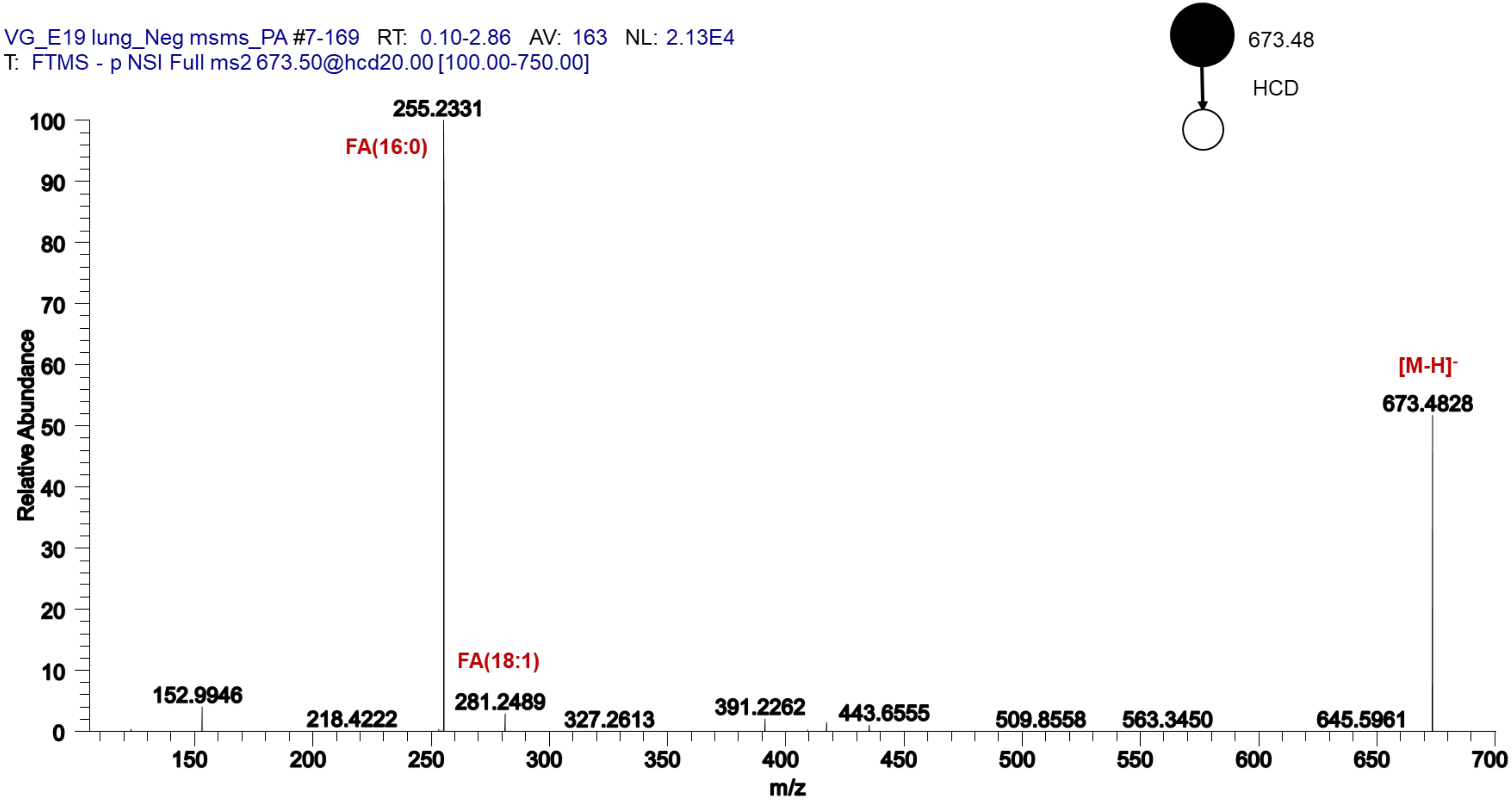

VG_E19 lung_Neg msms_PA #7-169 RT: 0.10-2.86 AV: 163 NL: 2.13E4
T: FTMS - p NSI Full ms2 673.50@hcd20.00 [100.00-750.00]
673.48
HCD
255.2331
FA(16:0)
[M-H]-
673.4828
FA(18:1)
152.9946
218.4222
281.2489
327.2613
391.2262
443.6555
509.8558
563.3450
645.5961
Relative Abundance
m/z

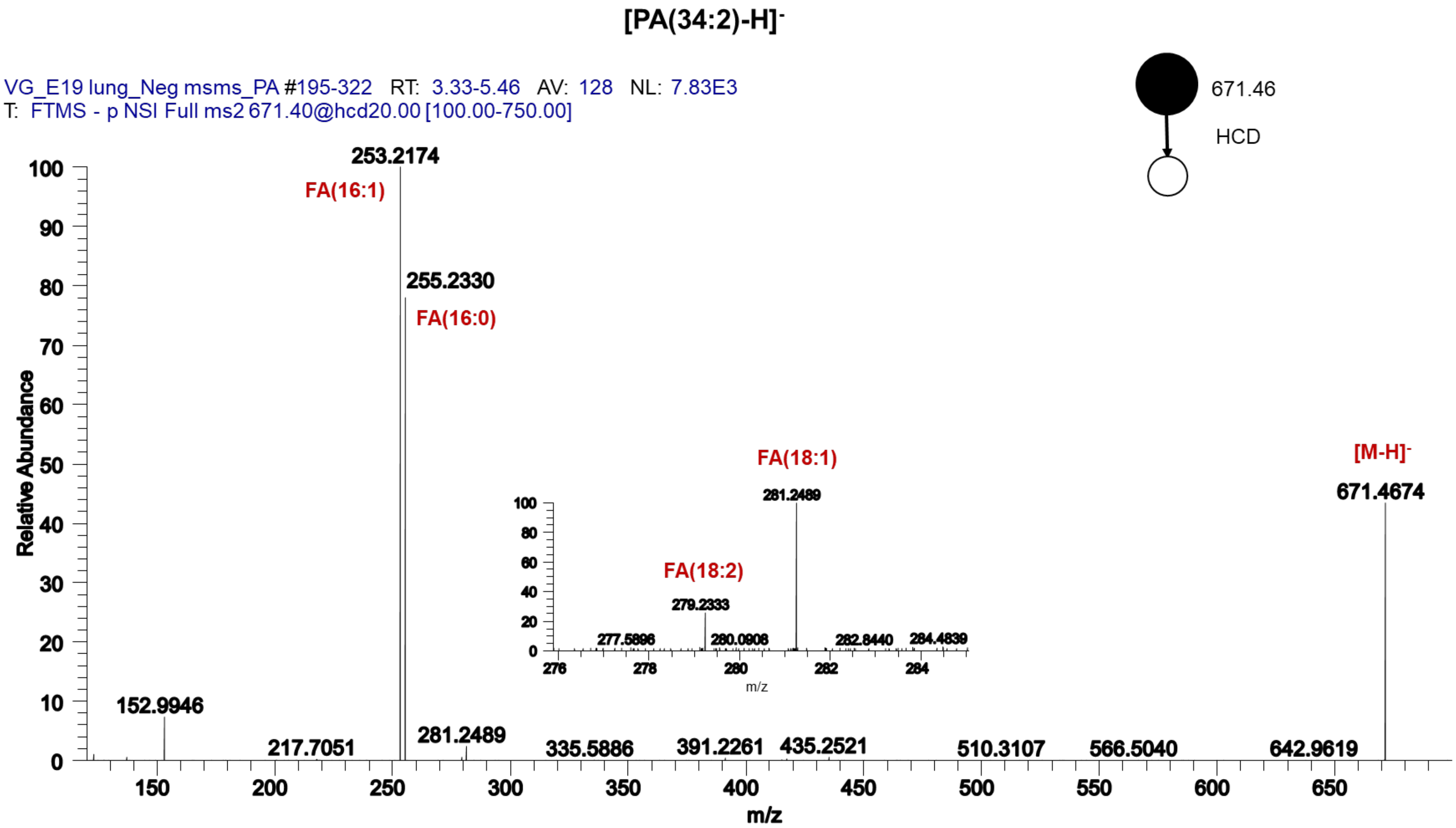
[PA(34:2)-H]-
VG_E19 lung_Neg msms_PA #195-322 RT: 3.33-5.46 AV: 128 NL: 7.83E3
T: FTMS - p NSI Full ms2 671.40@hcd20.00 [100.00-750.00]
671.46
HCD
Relative Abundance
253.2174
FA(16:1)
255.2330
FA(16:0)
152.9946
217.7051
281.2489
335.5886
391.2261
435.2521
510.3107
566.5040
642.9619
[M-H]-
671.4674
FA(18:1)
281.2489
FA(18:2)
279.2333
277.5896
280.0908
282.8440
284.4839
m/z
m/z

[PA(32:1)-H]$^-$

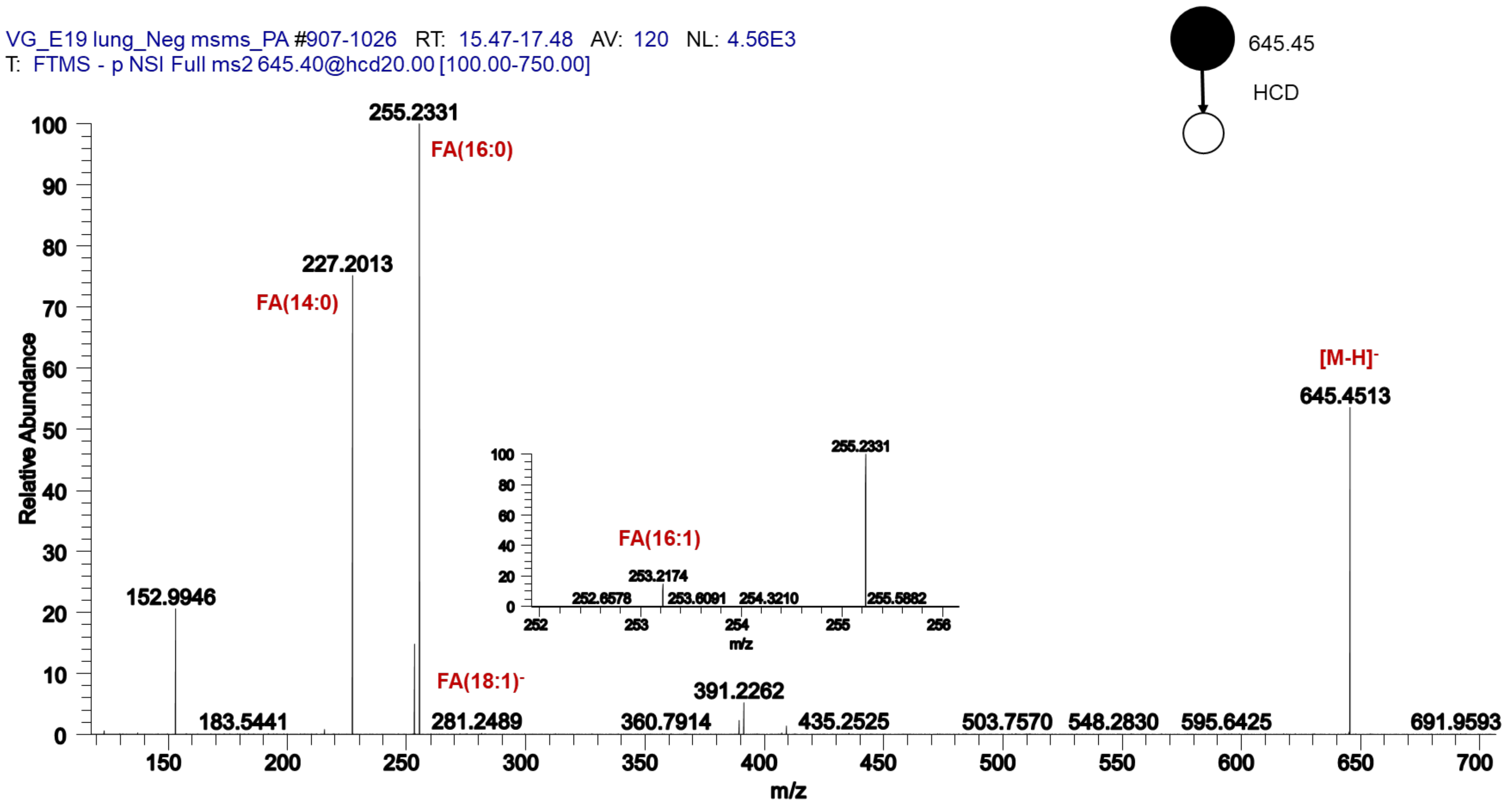

VG_E19 lung_Neg msms_PA #907-1026 RT: 15.47-17.48 AV: 120 NL: 4.56E3
T: FTMS - p NSI Full ms2 645.40@hcd20.00 [100.00-750.00]
645.45
HCD
255.2331
FA(16:0)
227.2013
FA(14:0)
[M-H]-
645.4513
152.9946
183.5441
FA(18:1)-
281.2489
360.7914
391.2262
435.2525
503.7570
548.2830
595.6425
691.9593
FA(16:1)
253.2174
252.6578
253.6091
254.3210
255.5882
Relative Abundance
m/z

[PA(36:2)-H]$^-$

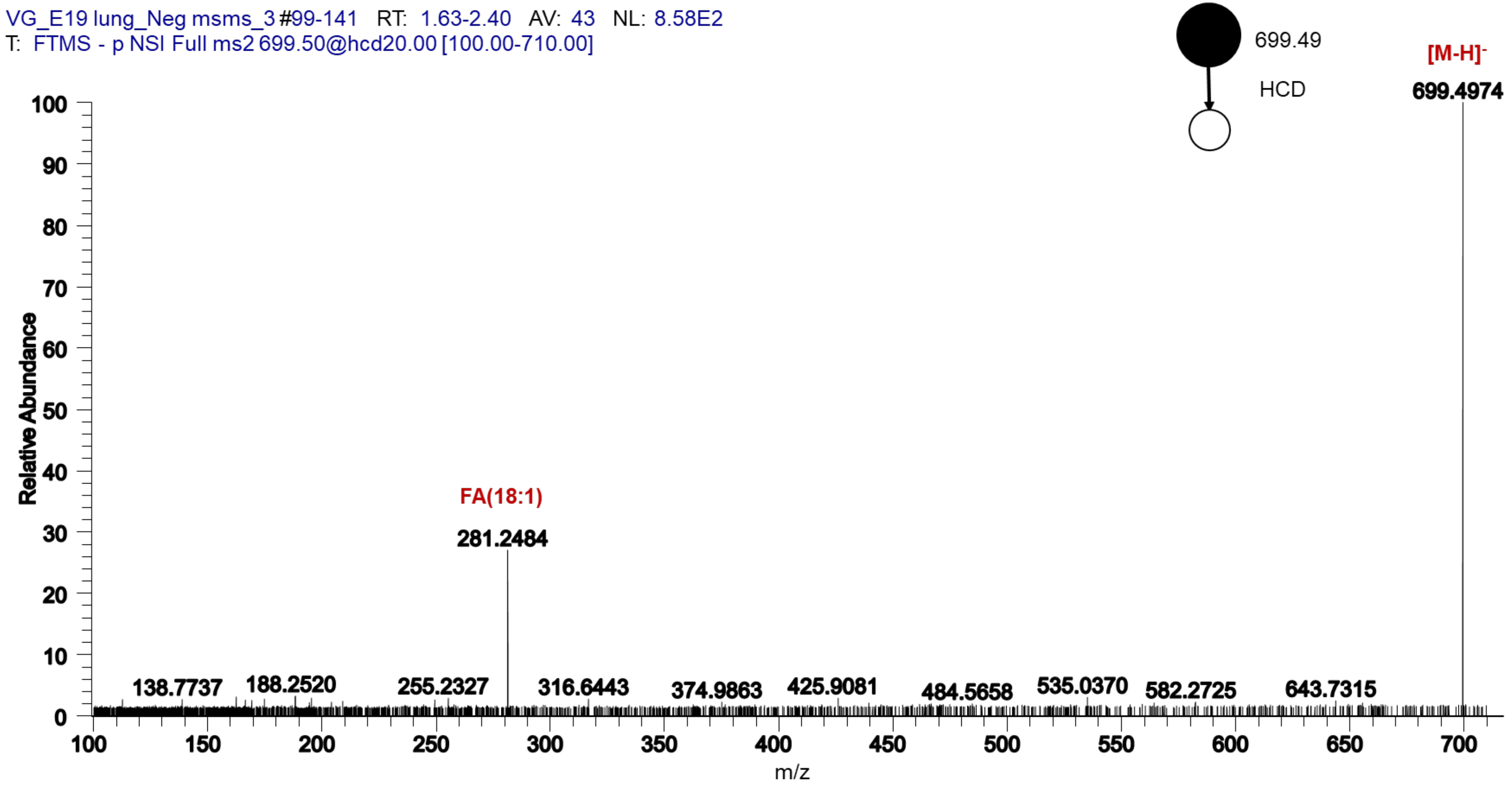
VG_E19 lung_Neg msms_3 #99-141 RT: 1.63-2.40 AV: 43 NL: 8.58E2
T: FTMS - p NSI Full ms2 699.50@hcd20.00 [100.00-710.00]
699.49
HCD
[M-H]-
699.4974
FA(18:1)
281.2484
138.7737
188.2520
255.2327
316.6443
374.9863
425.9081
484.5658
535.0370
582.2725
643.7315
Relative Abundance
m/z

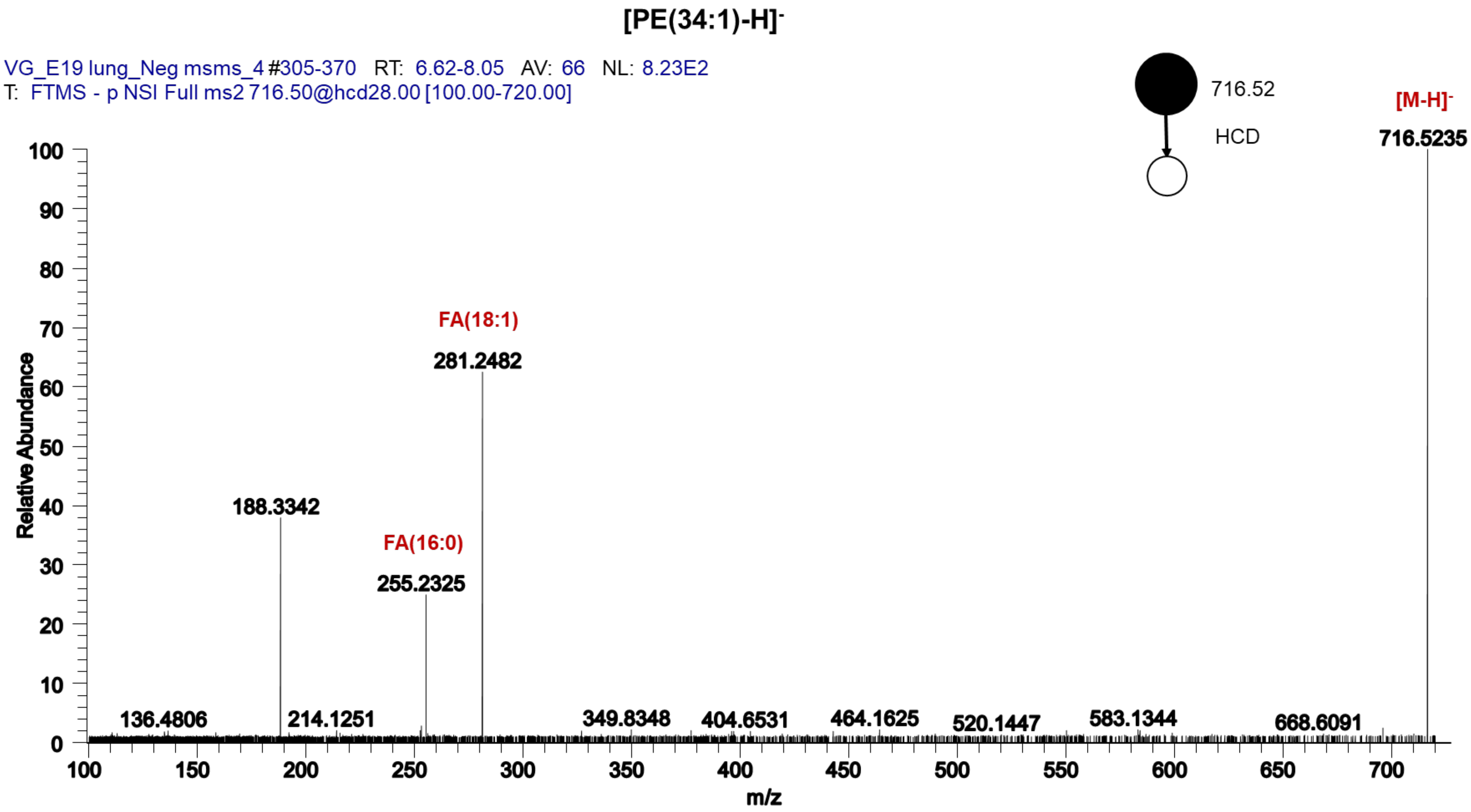
[PE(34:1)-H]-
VG_E19 lung_Neg msms_4 #305-370 RT: 6.62-8.05 AV: 66 NL: 8.23E2
T: FTMS - p NSI Full ms2 716.50@hcd28.00 [100.00-720.00]
716.52
HCD
[M-H]-
716.5235
FA(18:1)
281.2482
188.3342
FA(16:0)
255.2325
136.4806
214.1251
349.8348
404.6531
464.1625
520.1447
583.1344
668.6091
Relative Abundance
m/z

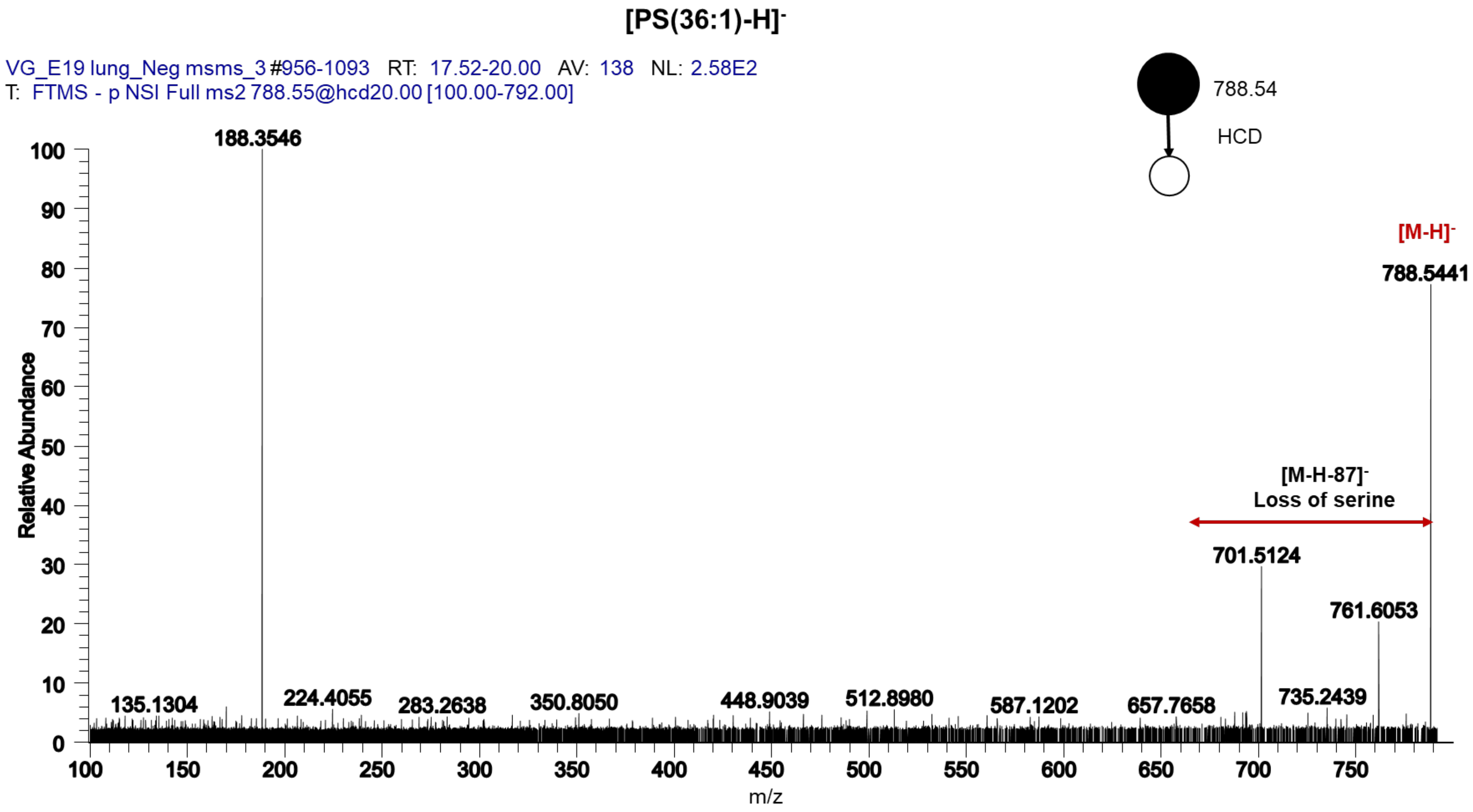
[PS(36:1)-H]-
VG_E19 lung_Neg msms_3 #956-1093 RT: 17.52-20.00 AV: 138 NL: 2.58E2
T: FTMS - p NSI Full ms2 788.55@hcd20.00 [100.00-792.00]
788.54
HCD
[M-H]-
788.5441
[M-H-87]-
Loss of serine
188.3546
701.5124
761.6053
135.1304
224.4055
283.2638
350.8050
448.9039
512.8980
587.1202
657.7658
735.2439
Relative Abundance
m/z

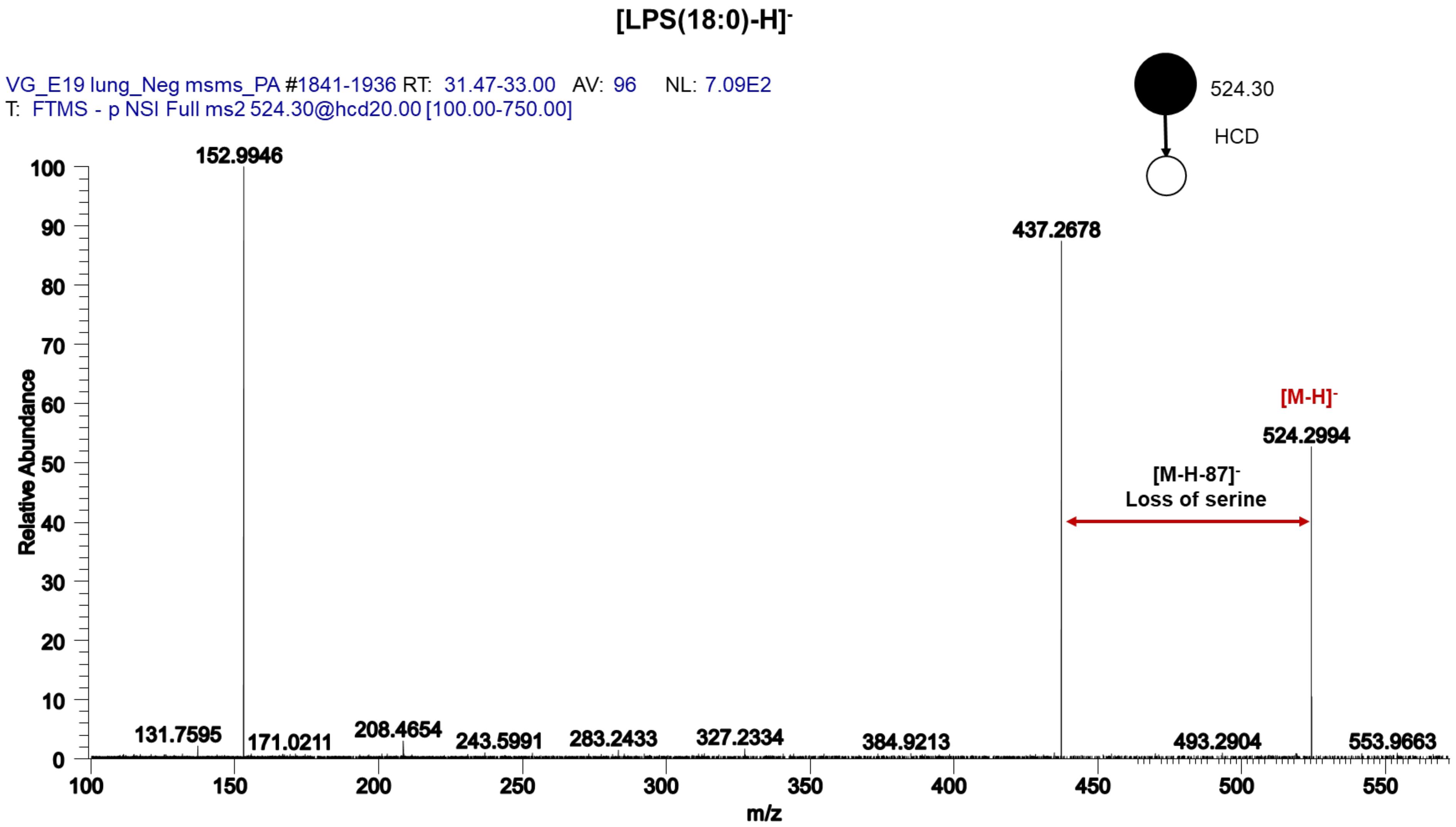
[LPS(18:0)-H]-
VG_E19 lung_Neg msms_PA #1841-1936 RT: 31.47-33.00 AV: 96 NL: 7.09E2
T: FTMS - p NSI Full ms2 524.30@hcd20.00 [100.00-750.00]
524.30
HCD
152.9946
437.2678
[M-H]-
524.2994
[M-H-87]-
Loss of serine
131.7595
171.0211
208.4654
243.5991
283.2433
327.2334
384.9213
493.2904
553.9663
Relative Abundance
m/z

[CDP-Choline+H]$^+$

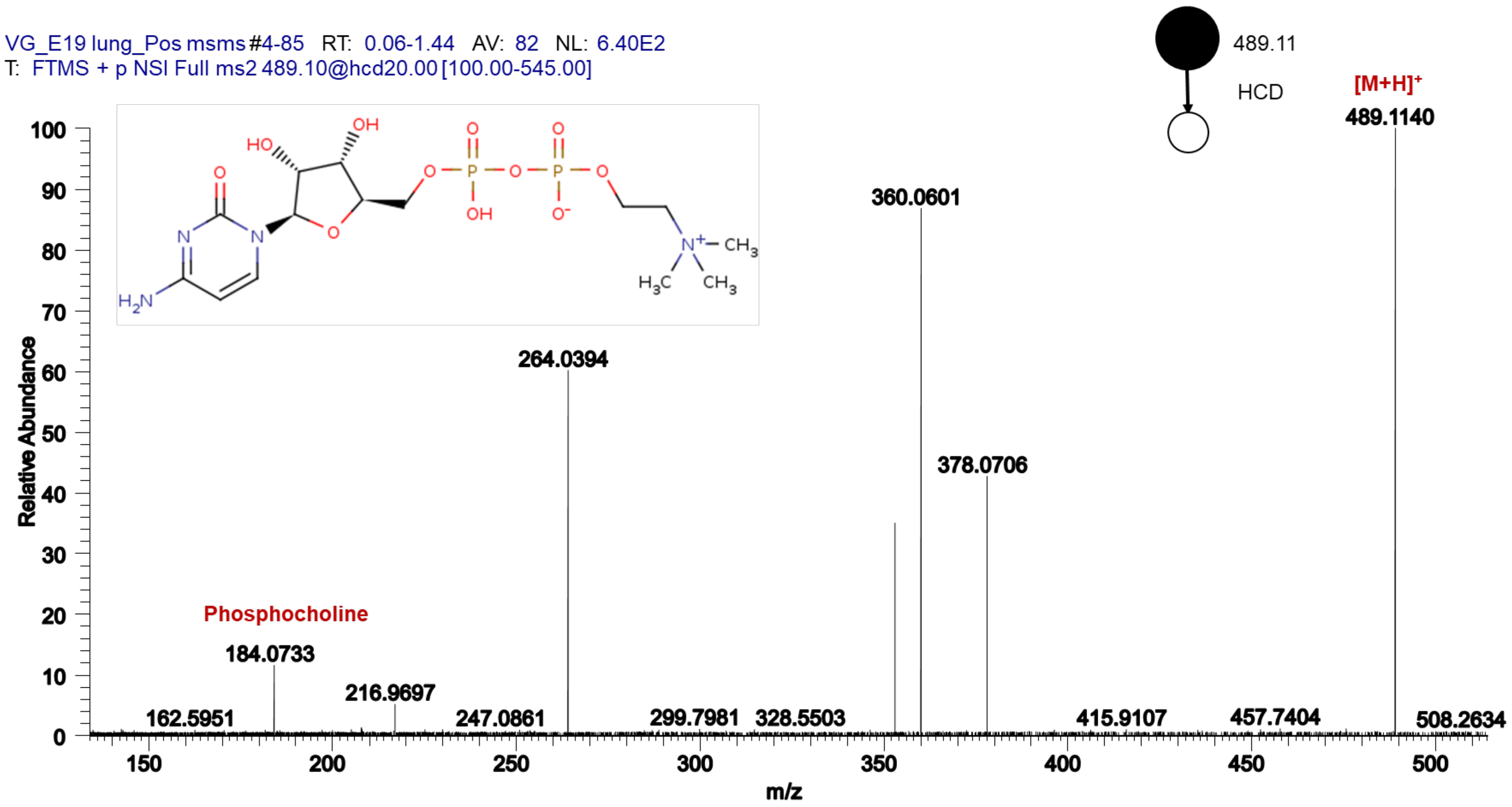

[CDP-Choline+Na]+

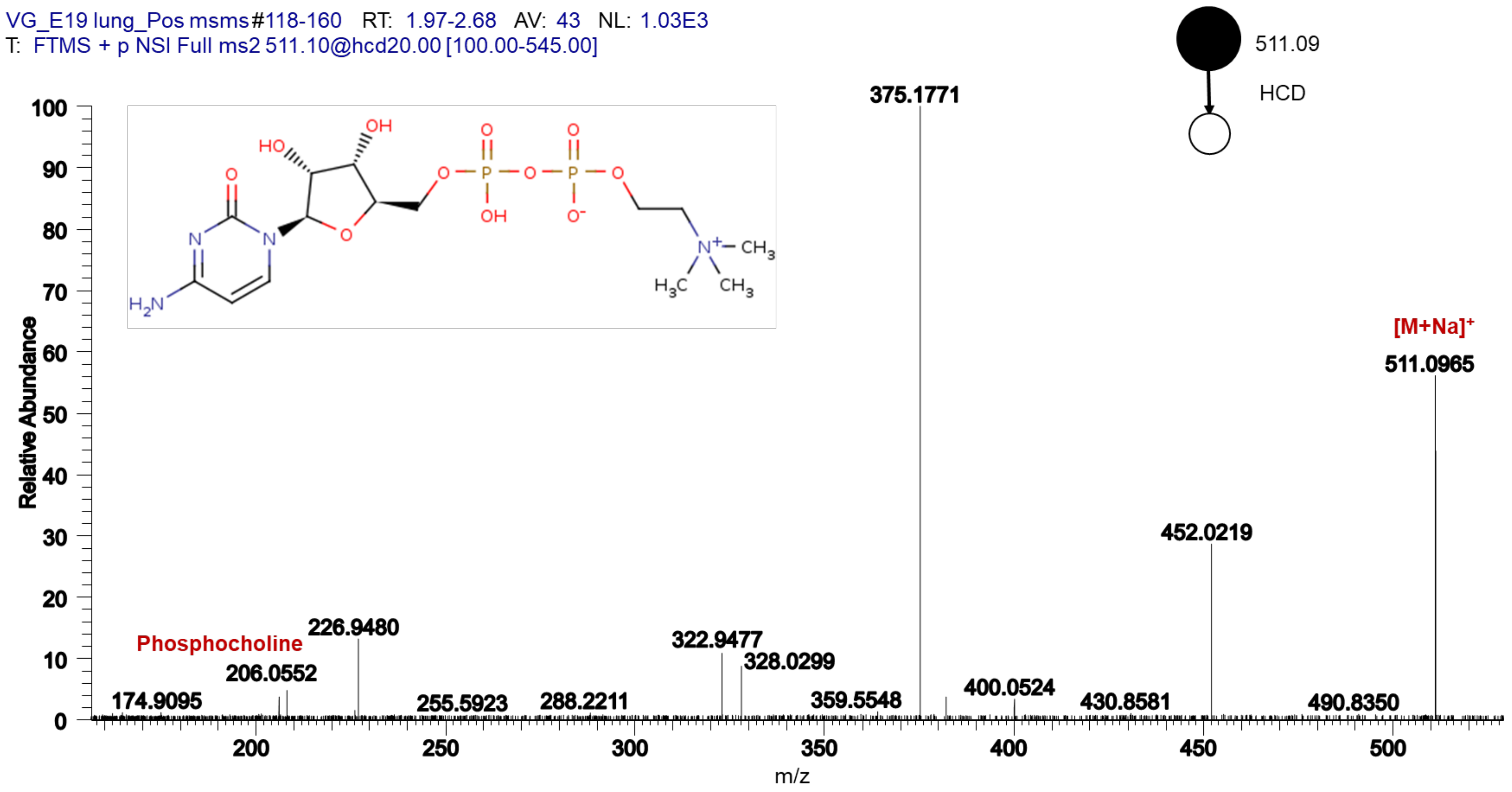

[CDP-Choline+K]$^+$

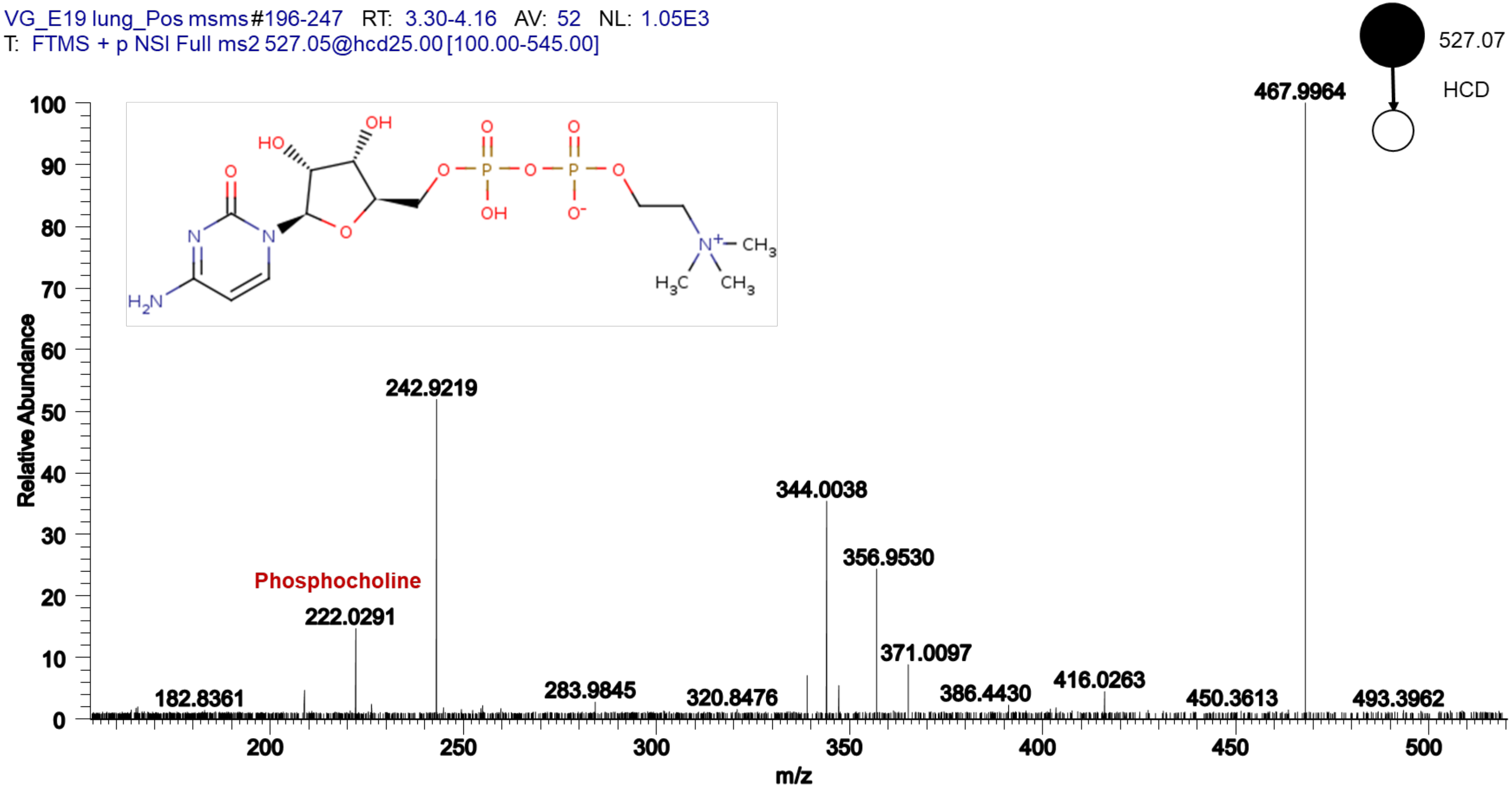

[LPC(16:0)+H]+

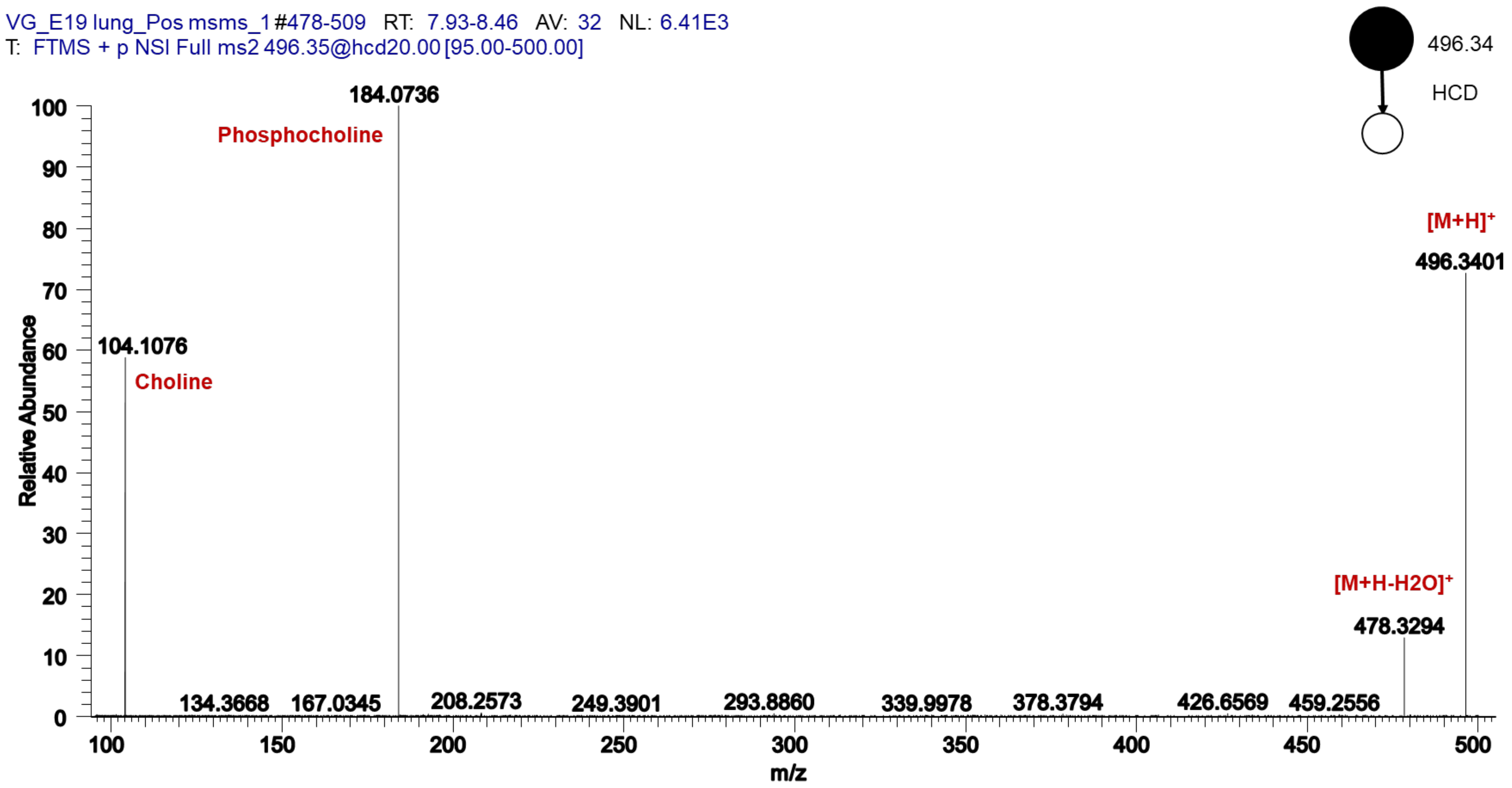
VG_E19 lung_Pos msms_1 #478-509 RT: 7.93-8.46 AV: 32 NL: 6.41E3
T: FTMS + p NSI Full ms2 496.35@hcd20.00 [95.00-500.00]
496.34
HCD
184.0736
Phosphocholine
104.1076
Choline
[M+H]+
496.3401
[M+H-H2O]+
478.3294
134.3668
167.0345
208.2573
249.3901
293.8860
339.9978
378.3794
426.6569
459.2556
Relative Abundance
m/z

$[LPC(16:0)+Na]^+$

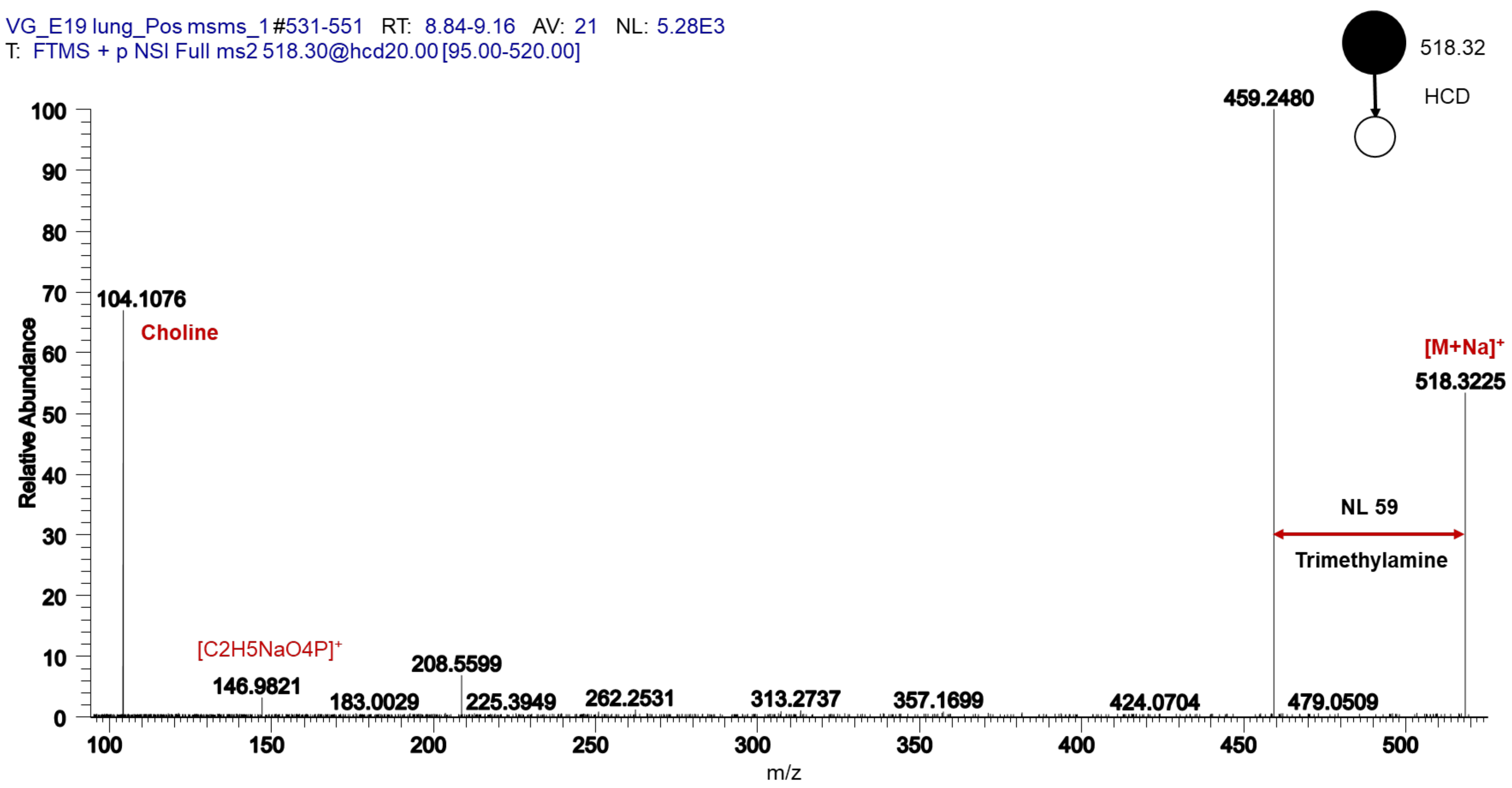

VG_E19 lung_Pos msms_1 #531-551 RT: 8.84-9.16 AV: 21 NL: 5.28E3
T: FTMS + p NSI Full ms2 518.30@hcd20.00 [95.00-520.00]
518.32
HCD
459.2480
104.1076
Choline
[M+Na]+
518.3225
NL 59
Trimethylamine
[C2H5NaO4P]+
146.9821
208.5599
183.0029
225.3949
262.2531
313.2737
357.1699
424.0704
479.0509
Relative Abundance
m/z

$[LPC(16:0)+K]^+$

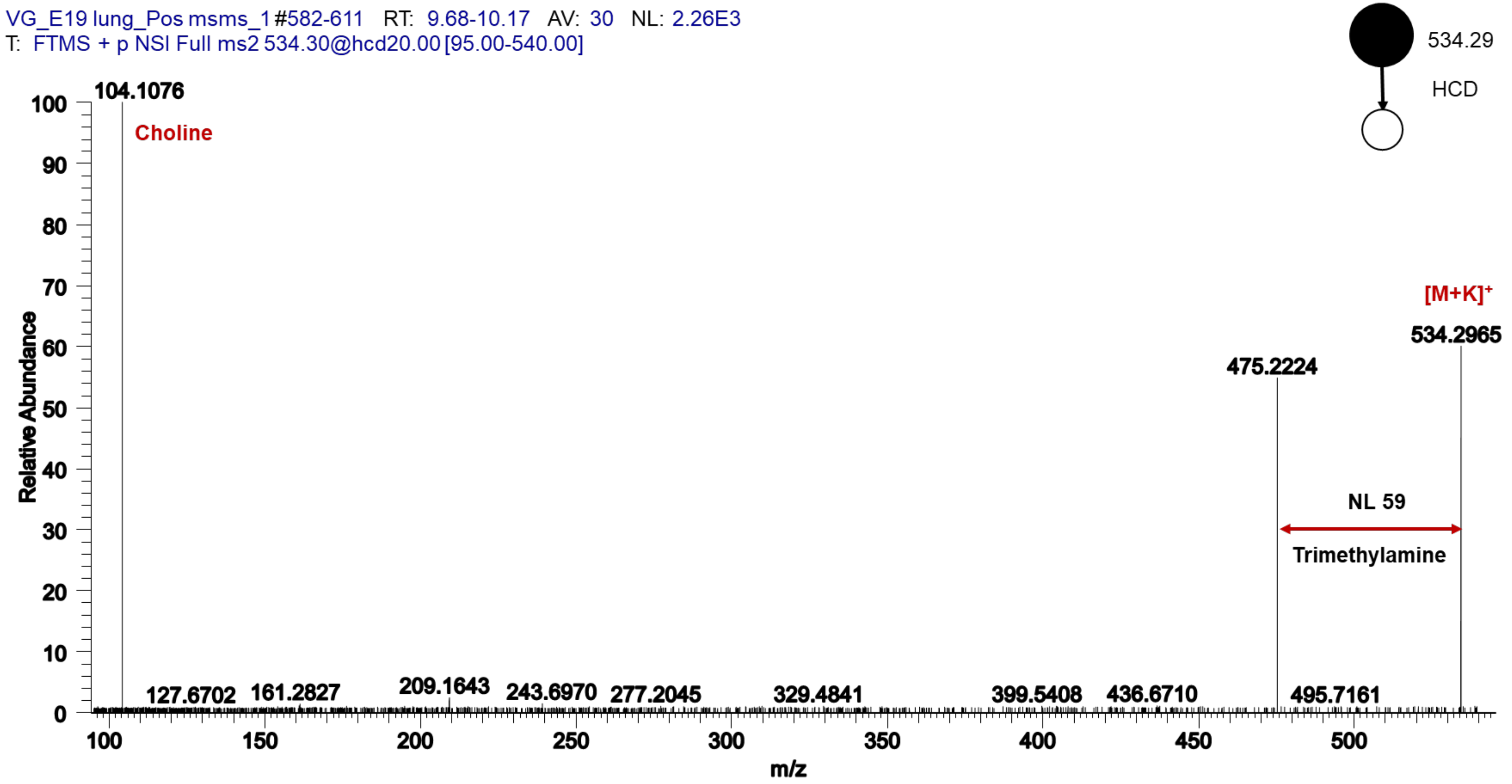

VG_E19 lung_Pos msms_1 #582-611 RT: 9.68-10.17 AV: 30 NL: 2.26E3
T: FTMS + p NSI Full ms2 534.30@hcd20.00 [95.00-540.00]
534.29
HCD
104.1076
Choline
[M+K]+
534.2965
475.2224
NL 59
Trimethylamine
127.6702
161.2827
209.1643
243.6970
277.2045
329.4841
399.5408
436.6710
495.7161
Relative Abundance
m/z

[LPC(16:1)+H]+

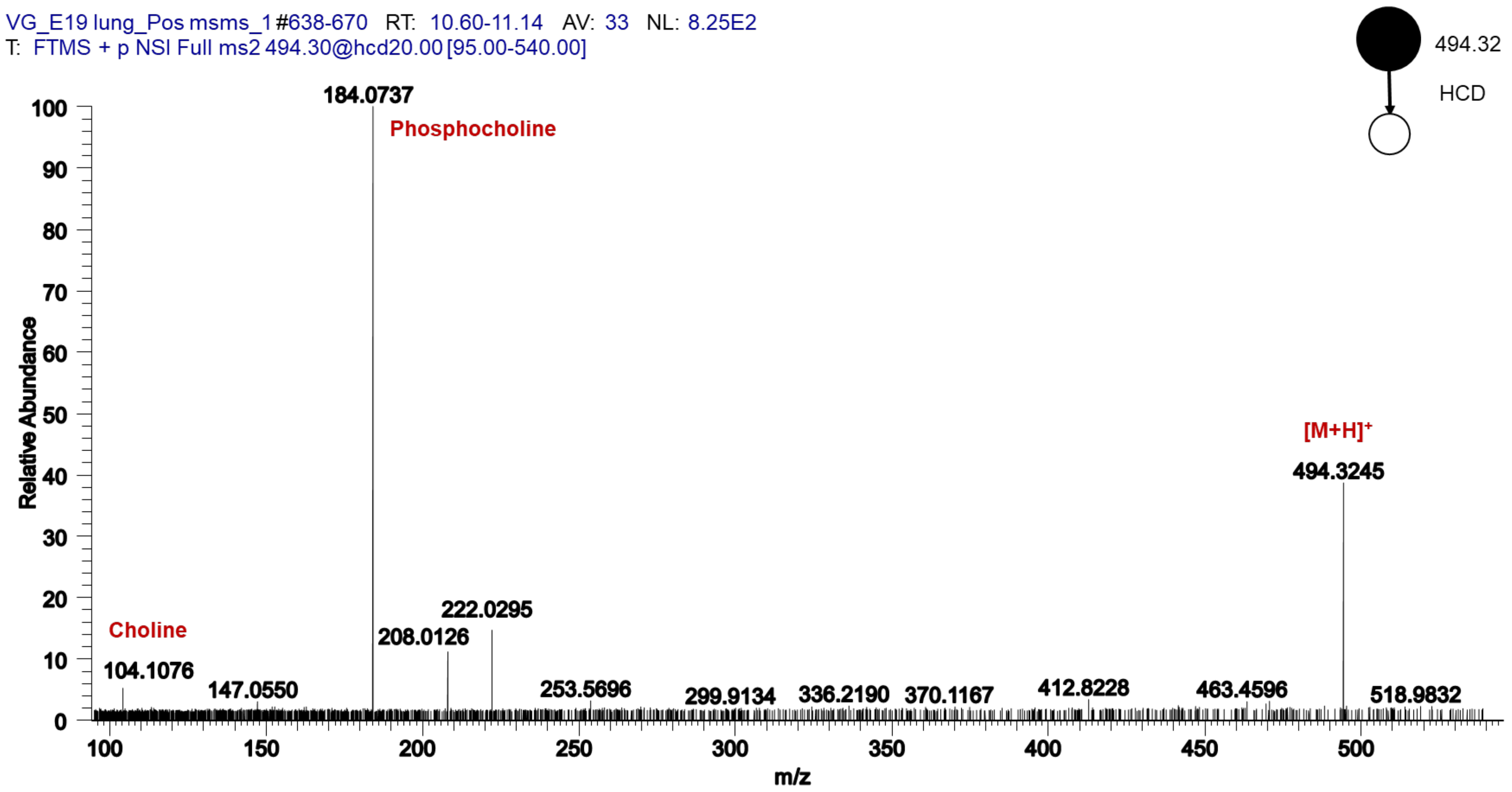
VG_E19 lung_Pos msms_1 #638-670 RT: 10.60-11.14 AV: 33 NL: 8.25E2
T: FTMS + p NSI Full ms2 494.30@hcd20.00 [95.00-540.00]
494.32
HCD
184.0737
Phosphocholine
[M+H]+
494.3245
222.0295
208.0126
Choline
104.1076
147.0550
253.5696
299.9134
336.2190
370.1167
412.8228
463.4596
518.9832
Relative Abundance
m/z

$[LPC(16:1)+Na]^+$

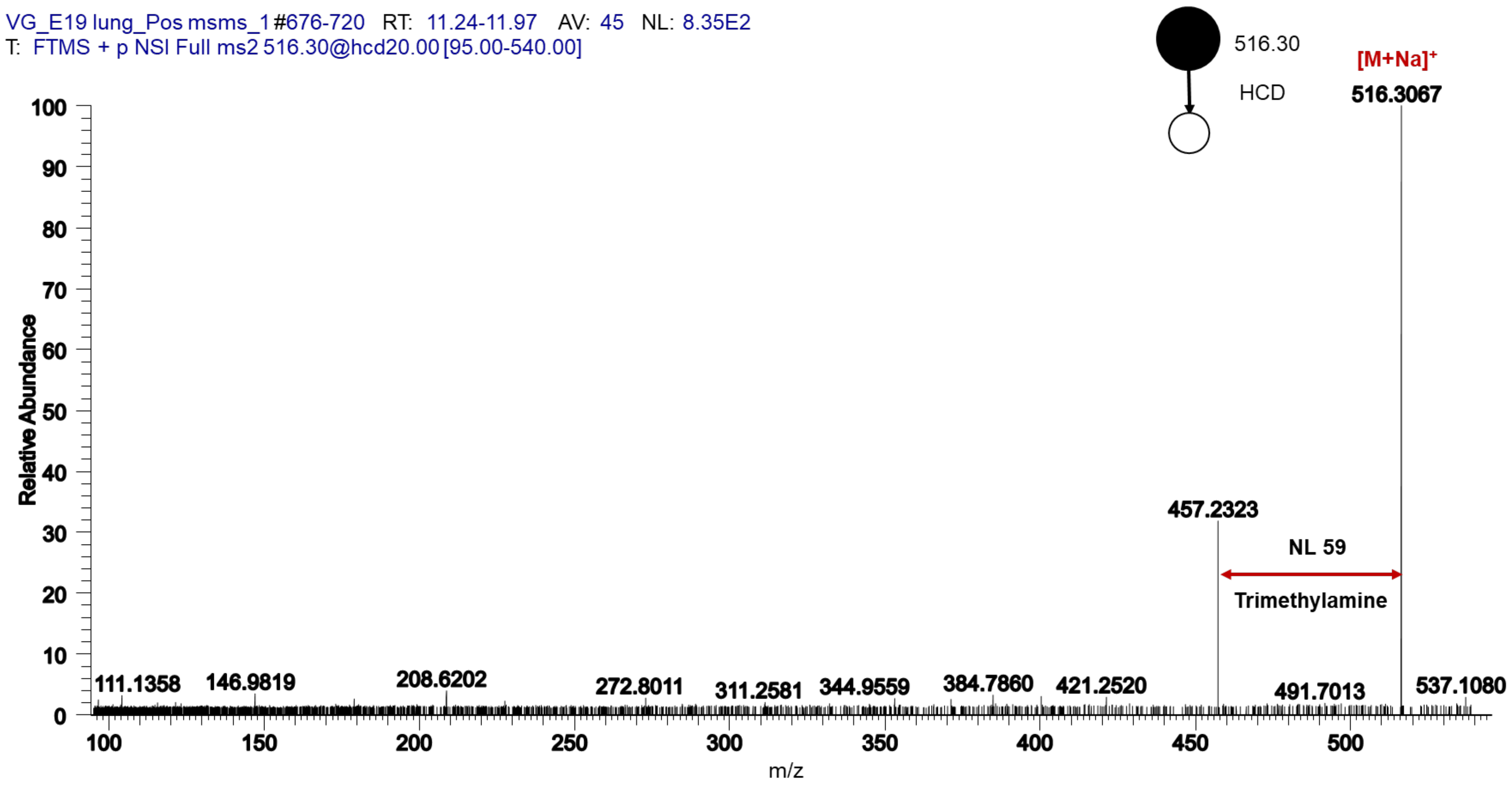

VG_E19 lung_Pos msms_1 #676-720 RT: 11.24-11.97 AV: 45 NL: 8.35E2
T: FTMS + p NSI Full ms2 516.30@hcd20.00 [95.00-540.00]
516.30
HCD
[M+Na]+
516.3067
457.2323
NL 59
Trimethylamine
111.1358
146.9819
208.6202
272.8011
311.2581
344.9559
384.7860
421.2520
491.7013
537.1080
Relative Abundance
m/z

$[PC(30:0)+H]^+$

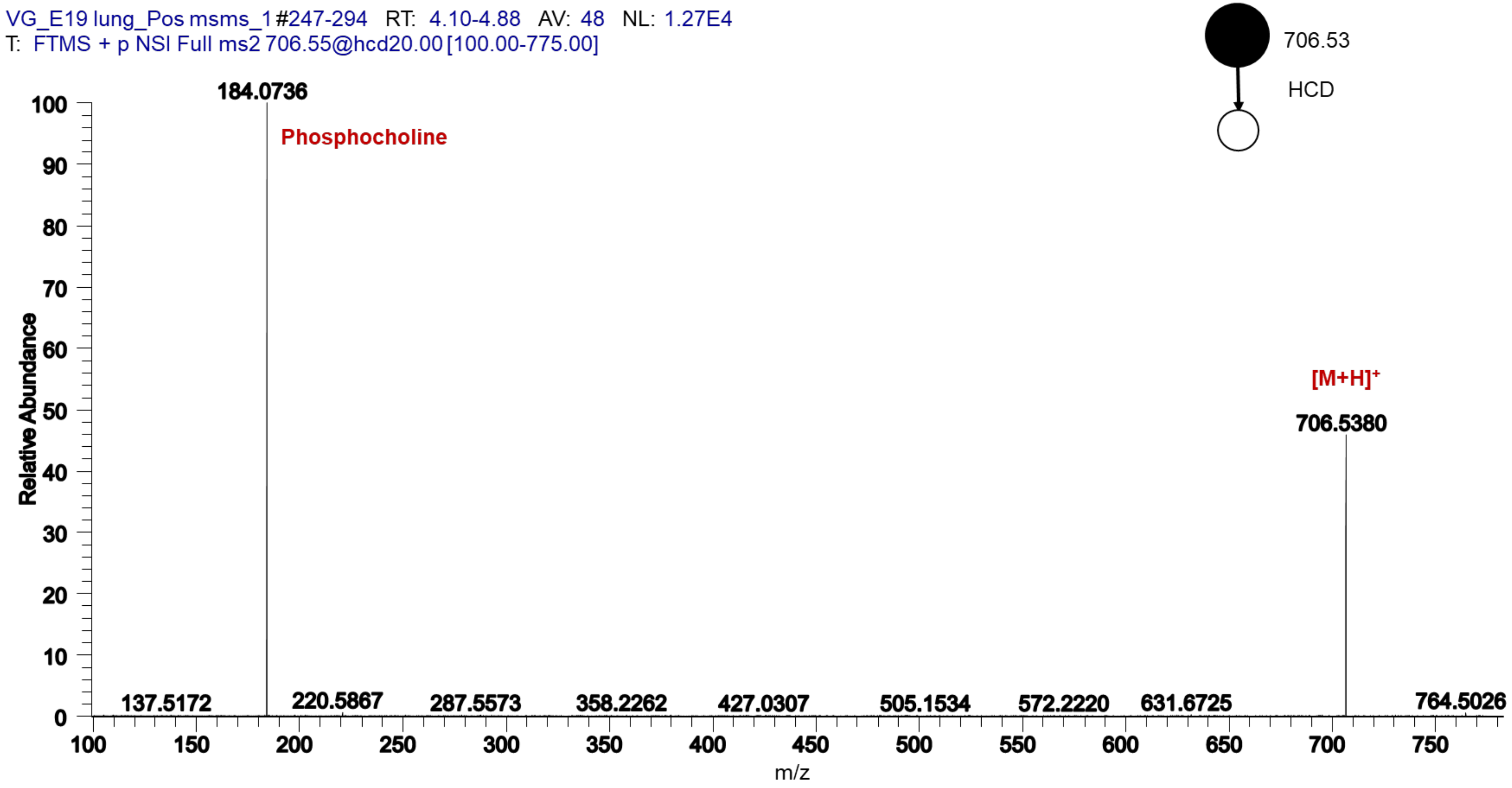

VG_E19 lung_Pos msms_1 #247-294 RT: 4.10-4.88 AV: 48 NL: 1.27E4
T: FTMS + p NSI Full ms2 706.55@hcd20.00 [100.00-775.00]
706.53
HCD
184.0736
Phosphocholine
[M+H]+
706.5380
137.5172
220.5867
287.5573
358.2262
427.0307
505.1534
572.2220
631.6725
764.5026
Relative Abundance
m/z

[PC(30:0)+Na]$^+$

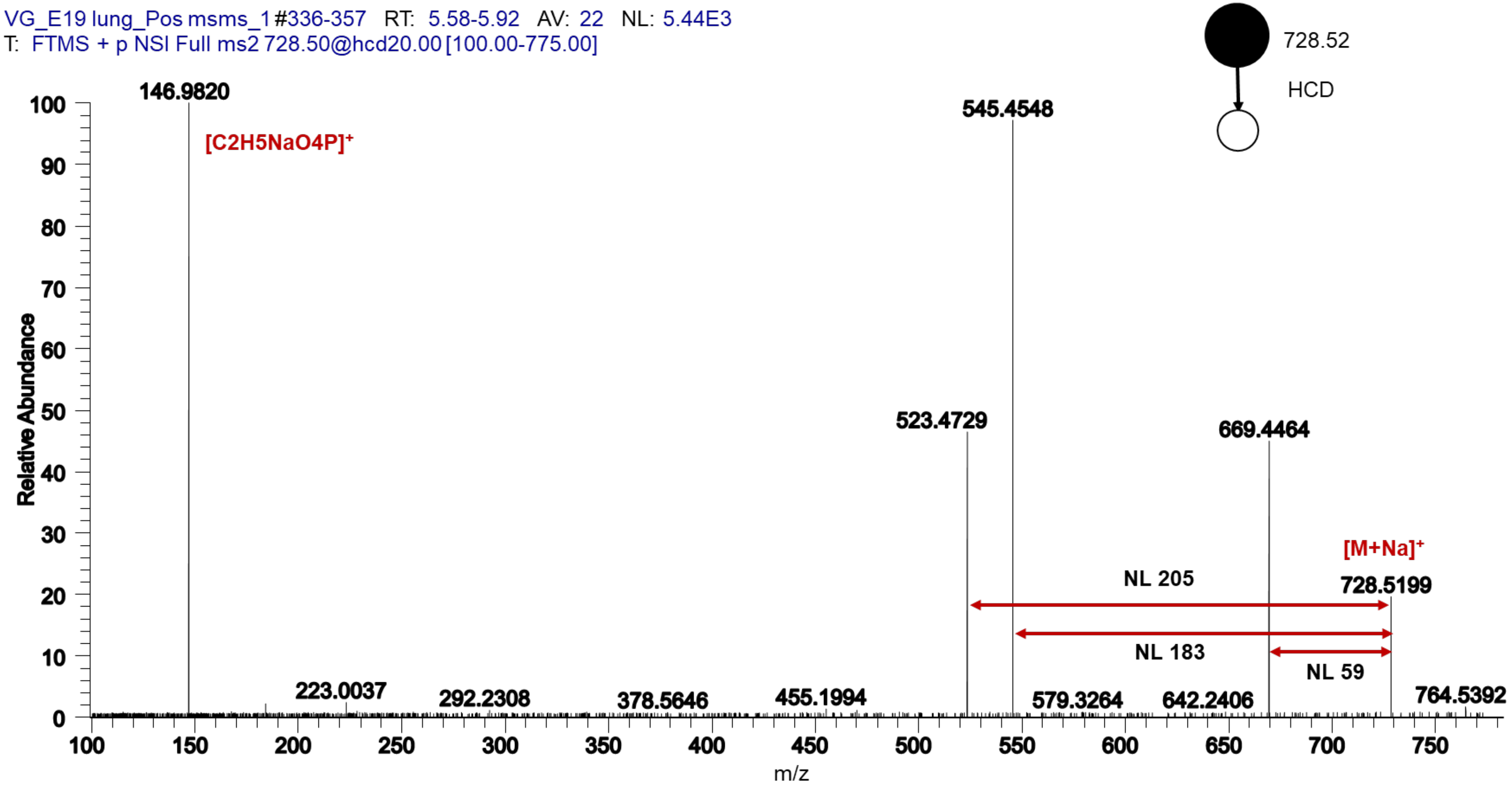
VG_E19 lung_Pos msms_1 #336-357 RT: 5.58-5.92 AV: 22 NL: 5.44E3
T: FTMS + p NSI Full ms2 728.50@hcd20.00 [100.00-775.00]
728.52
HCD
146.9820
[C2H5NaO4P]+
545.4548
523.4729
669.4464
[M+Na]+
728.5199
NL 205
NL 183
NL 59
223.0037
292.2308
378.5646
455.1994
579.3264
642.2406
764.5392
Relative Abundance
m/z

[PC(30:0)+K]$^+$

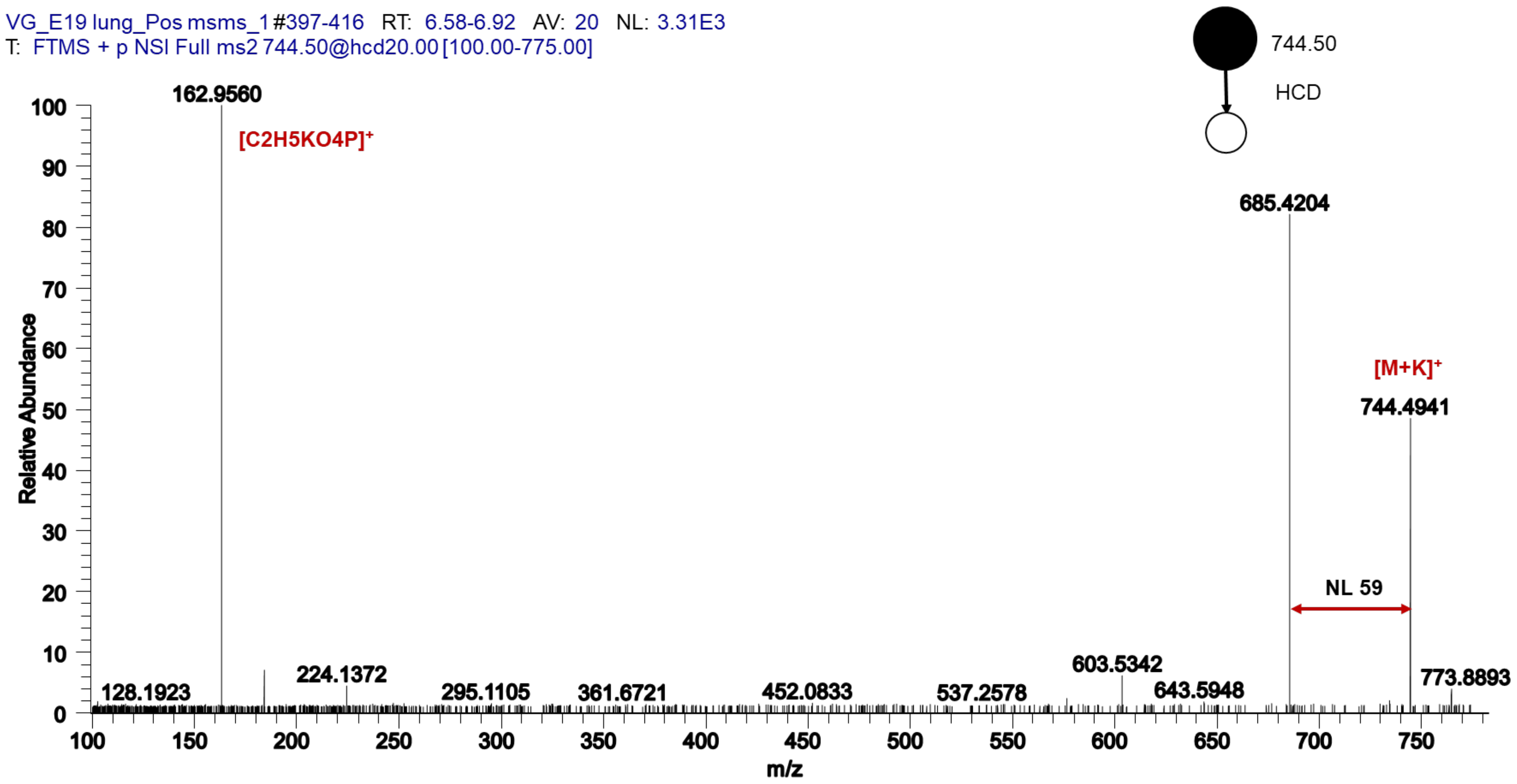

VG_E19 lung_Pos msms_1 #397-416 RT: 6.58-6.92 AV: 20 NL: 3.31E3
T: FTMS + p NSI Full ms2 744.50@hcd20.00 [100.00-775.00]
744.50
HCD
162.9560
[C2H5KO4P]+
685.4204
[M+K]+
744.4941
NL 59
128.1923
224.1372
295.1105
361.6721
452.0833
537.2578
603.5342
643.5948
773.8893
Relative Abundance
m/z

[PC(32:2)+H]⁺

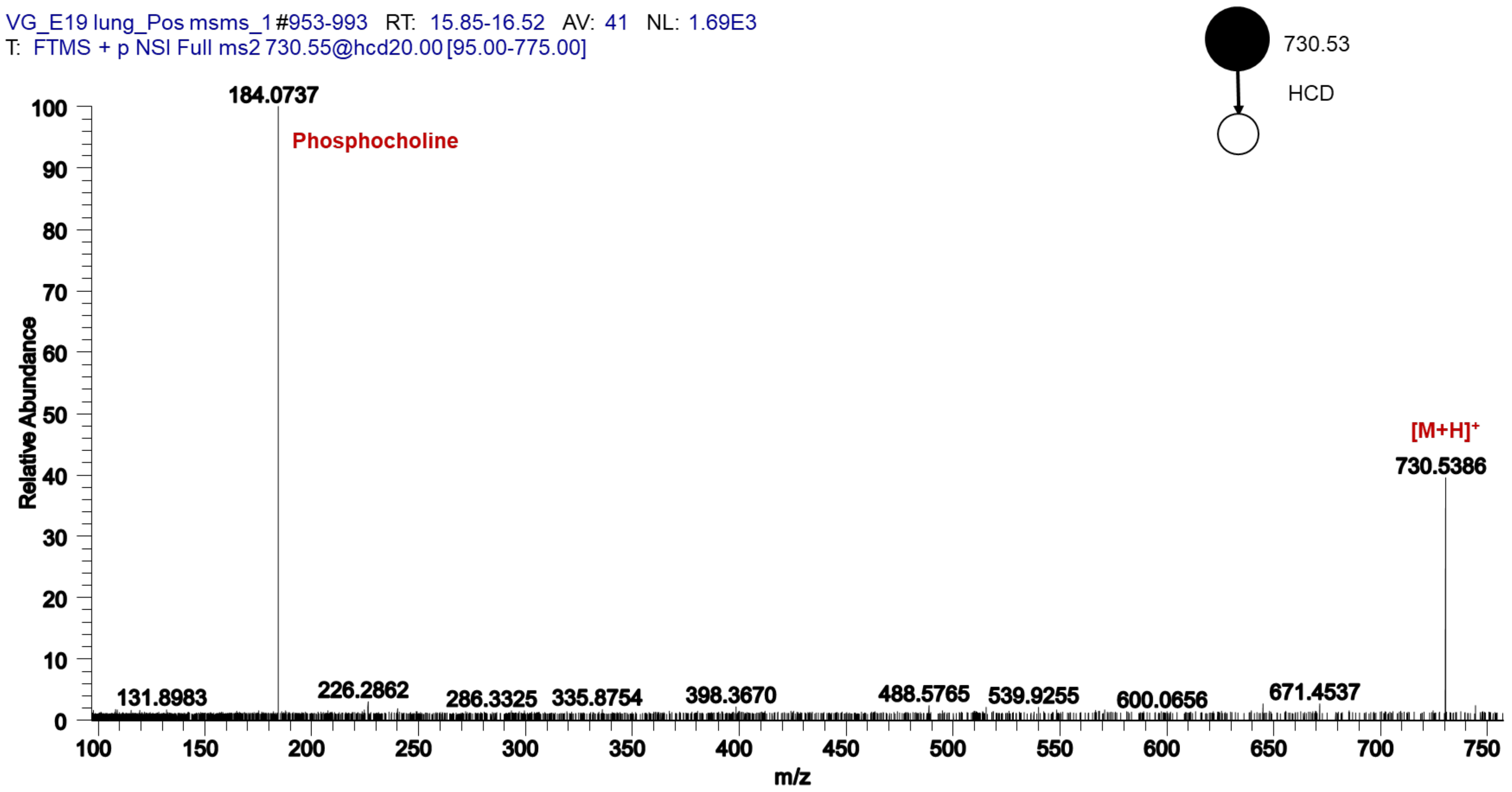

VG_E19 lung_Pos msms_1 #953-993 RT: 15.85-16.52 AV: 41 NL: 1.69E3
T: FTMS + p NSI Full ms2 730.55@hcd20.00 [95.00-775.00]
730.53
HCD
184.0737
Phosphocholine
[M+H]+
730.5386
131.8983
226.2862
286.3325
335.8754
398.3670
488.5765
539.9255
600.0656
671.4537
Relative Abundance
m/z

[PC(32:2)+Na]$^+$

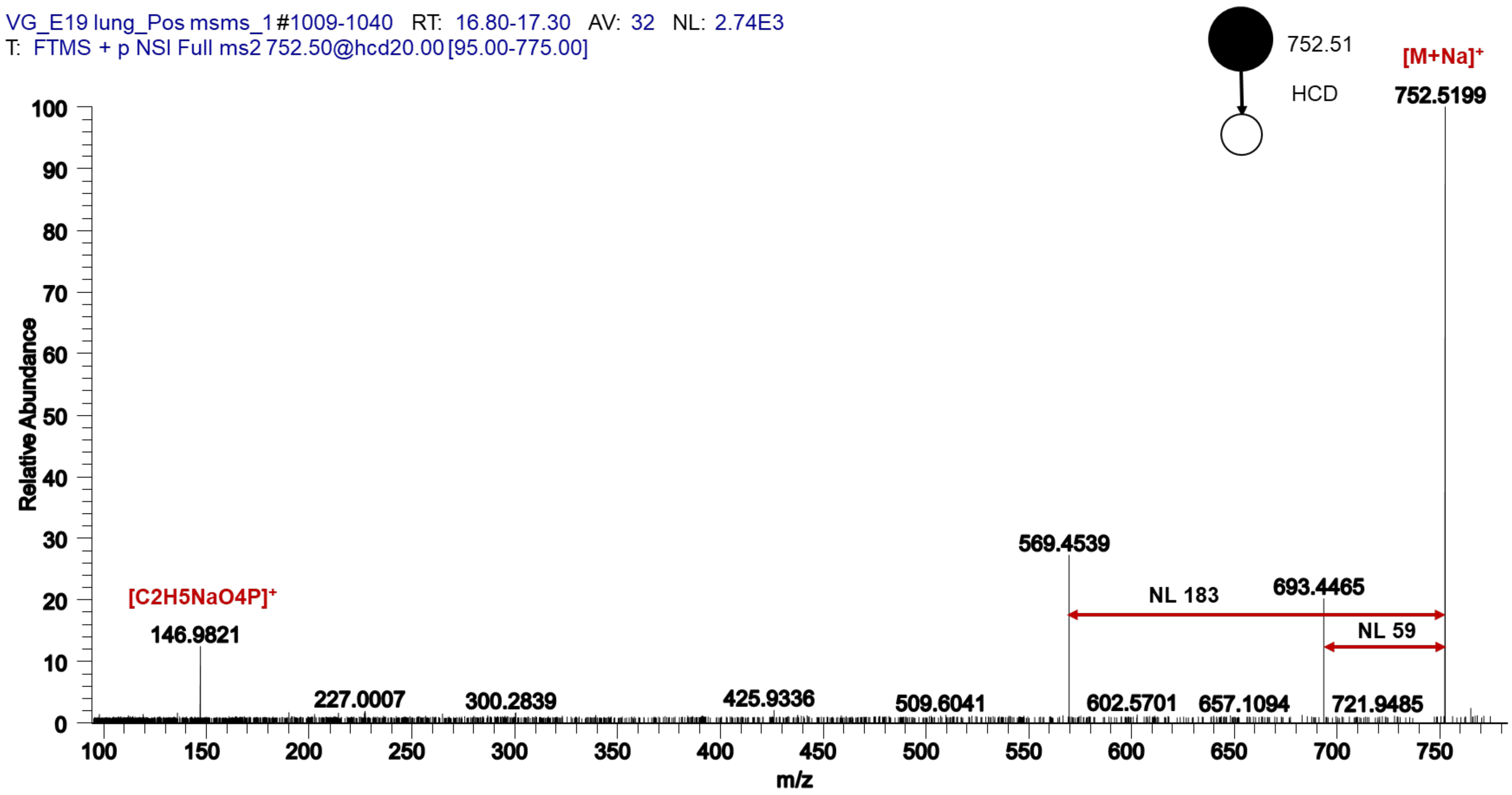

VG_E19 lung_Pos msms_1 #1009-1040 RT: 16.80-17.30 AV: 32 NL: 2.74E3
T: FTMS + p NSI Full ms2 752.50@hcd20.00 [95.00-775.00]
752.51
HCD
[M+Na]+
752.5199
569.4539
NL 183
693.4465
NL 59
[C2H5NaO4P]+
146.9821
227.0007
300.2839
425.9336
509.6041
602.5701
657.1094
721.9485
Relative Abundance
m/z

[PC(32:1)+H]+

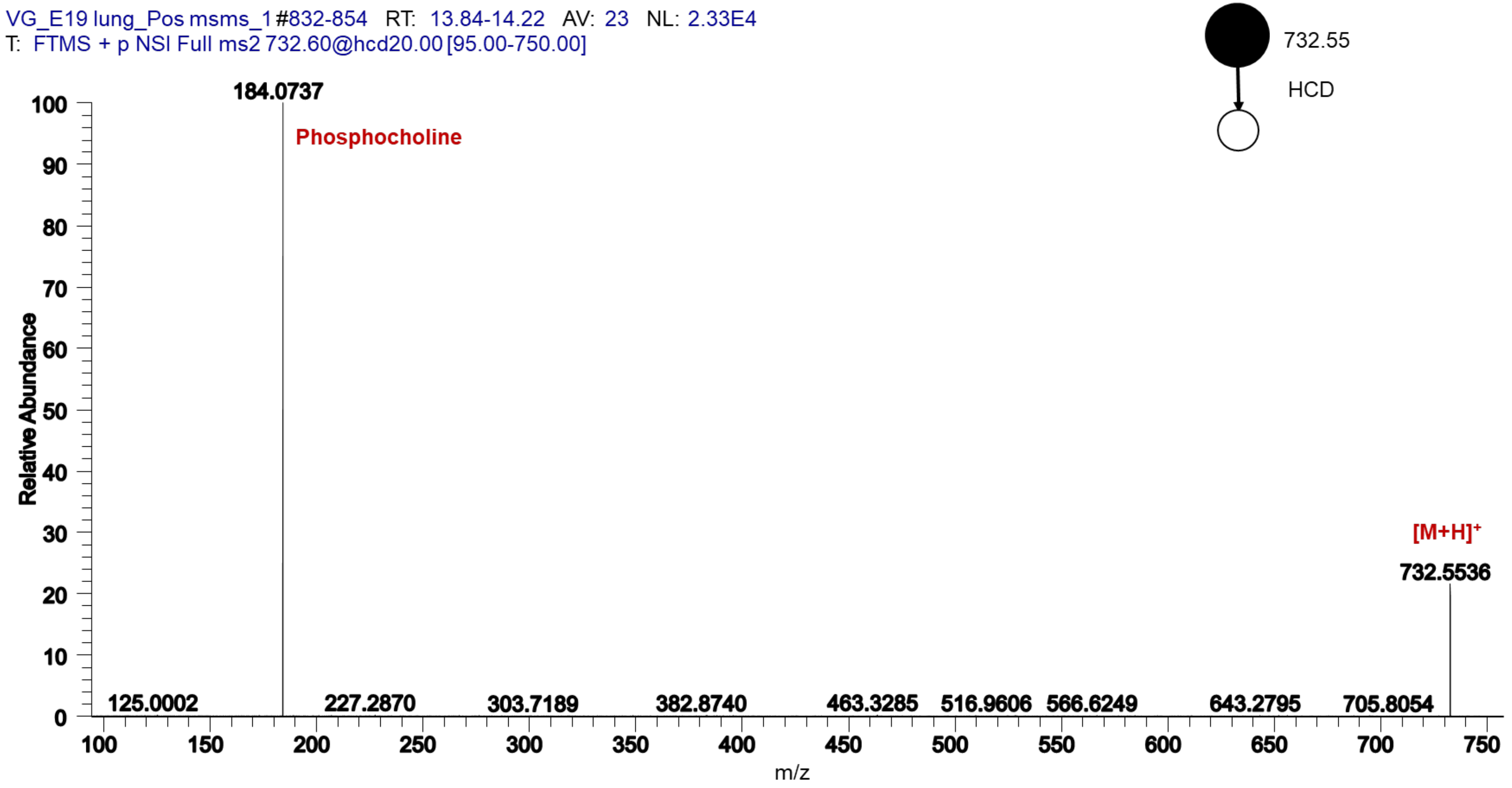
VG_E19 lung_Pos msms_1 #832-854 RT: 13.84-14.22 AV: 23 NL: 2.33E4
T: FTMS + p NSI Full ms2 732.60@hcd20.00 [95.00-750.00]
732.55
HCD
184.0737
Phosphocholine
[M+H]+
732.5536
125.0002
227.2870
303.7189
382.8740
463.3285
516.9606
566.6249
643.2795
705.8054
Relative Abundance
m/z

[PC(32:1)+Na]+

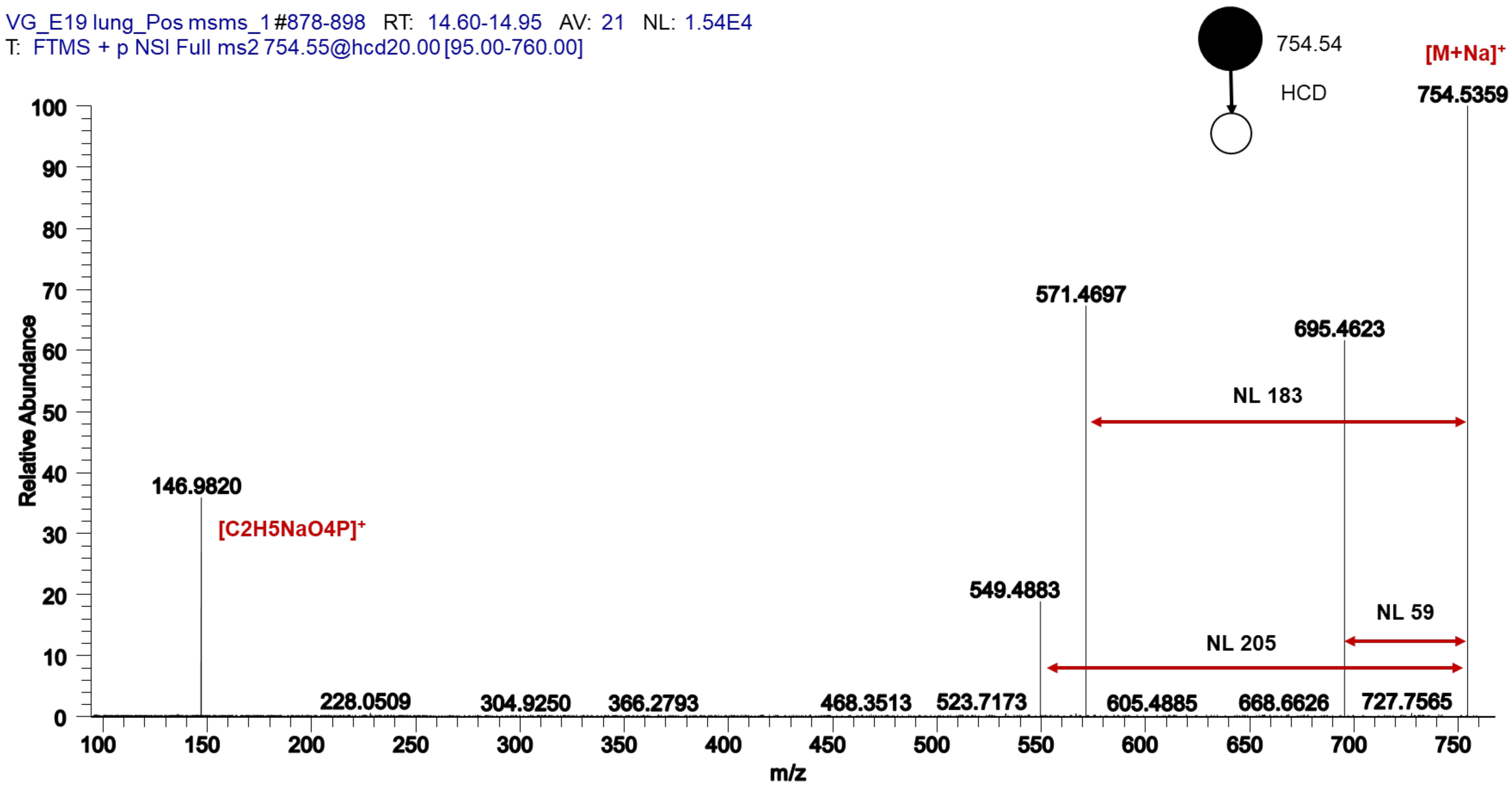

VG_E19 lung_Pos msms_1 #878-898 RT: 14.60-14.95 AV: 21 NL: 1.54E4
T: FTMS + p NSI Full ms2 754.55@hcd20.00 [95.00-760.00]
754.54
HCD
[M+Na]+
754.5359
571.4697
695.4623
NL 183
146.9820
[C2H5NaO4P]+
549.4883
NL 59
NL 205
228.0509
304.9250
366.2793
468.3513
523.7173
605.4885
668.6626
727.7565
Relative Abundance
m/z

[PC(32:1)+K]$^+$

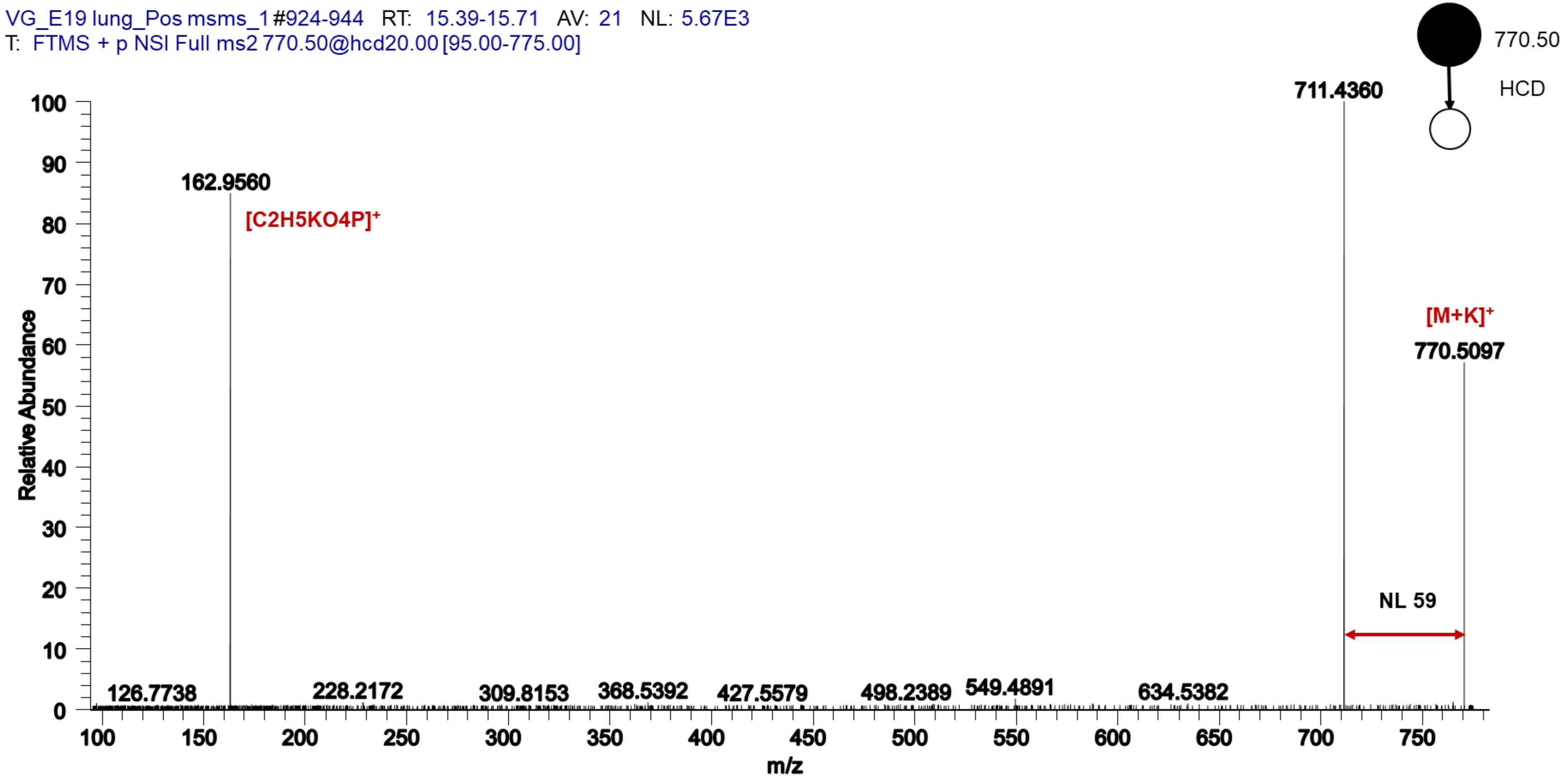
VG_E19 lung_Pos msms_1 #924-944 RT: 15.39-15.71 AV: 21 NL: 5.67E3
T: FTMS + p NSI Full ms2 770.50@hcd20.00 [95.00-775.00]
770.50
HCD
711.4360
162.9560
[C2H5KO4P]+
[M+K]+
770.5097
NL 59
126.7738
228.2172
309.8153
368.5392
427.5579
498.2389
549.4891
634.5382
Relative Abundance
m/z

$[PC(32:0)+H]^+$

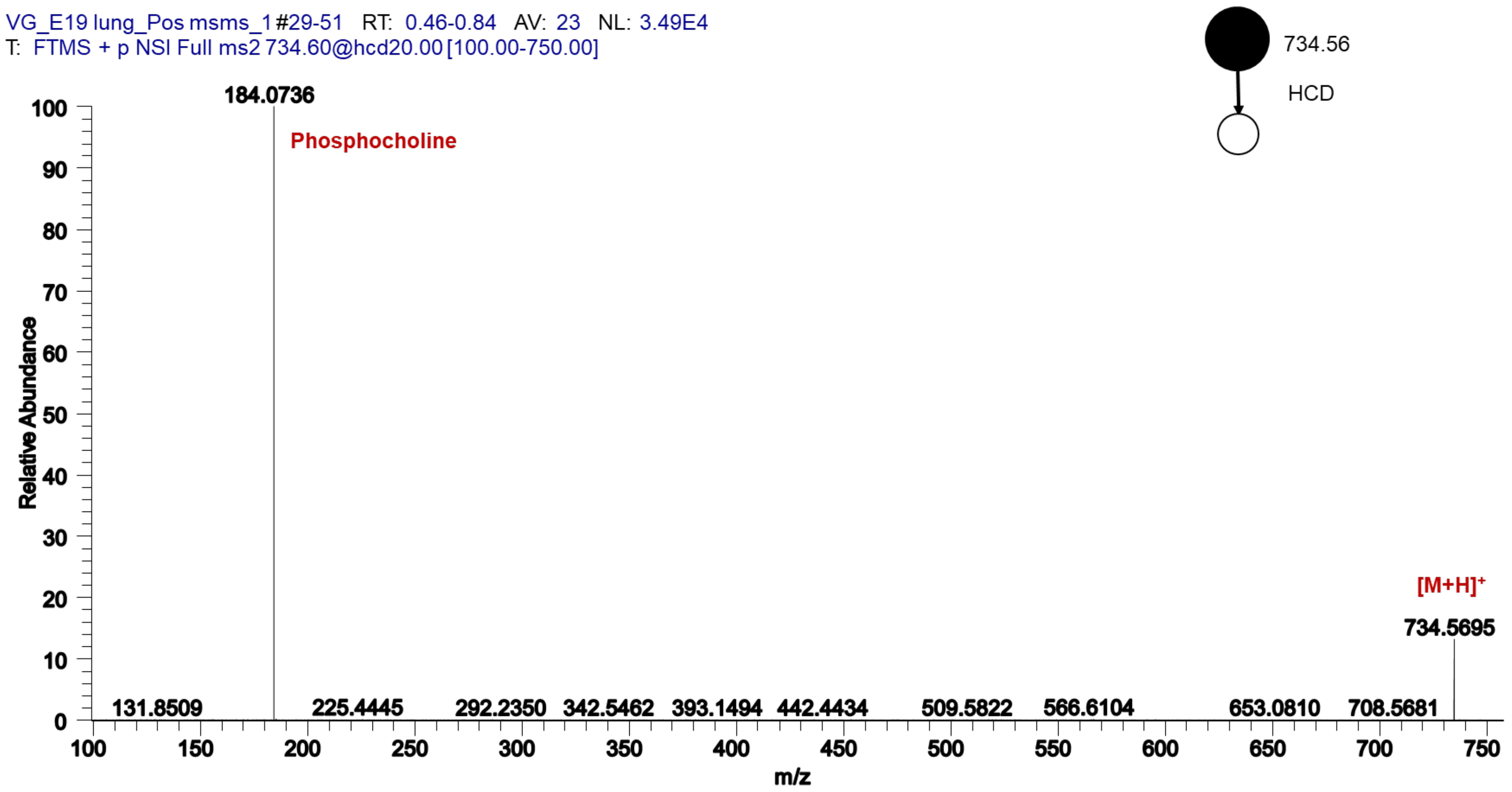

VG_E19 lung_Pos msms_1 #29-51 RT: 0.46-0.84 AV: 23 NL: 3.49E4
T: FTMS + p NSI Full ms2 734.60@hcd20.00 [100.00-750.00]
734.56
HCD
184.0736
Phosphocholine
[M+H]+
734.5695
131.8509
225.4445
292.2350
342.5462
393.1494
442.4434
509.5822
566.6104
653.0810
708.5681
Relative Abundance
m/z
0
10
20
30
40
50
60
70
80
90
100
150
200
250
300
350
400
450
500
550
600
650
700
750

[PC(32:0)+Na]⁺

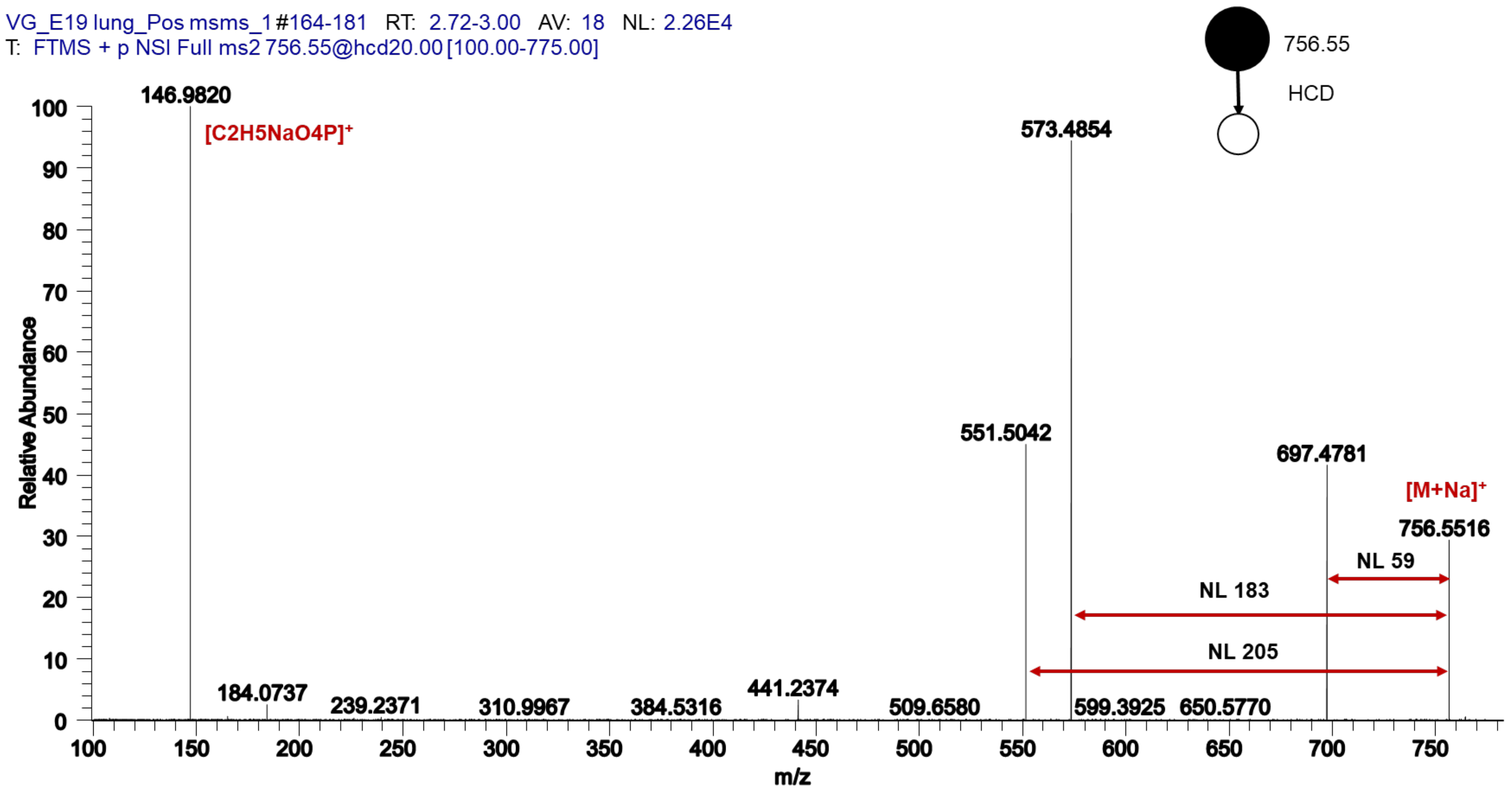

VG_E19 lung_Pos msms_1 #164-181 RT: 2.72-3.00 AV: 18 NL: 2.26E4
T: FTMS + p NSI Full ms2 756.55@hcd20.00 [100.00-775.00]
756.55
HCD
146.9820
[C2H5NaO4P]+
573.4854
551.5042
697.4781
[M+Na]+
756.5516
NL 59
NL 183
NL 205
184.0737
239.2371
310.9967
384.5316
441.2374
509.6580
599.3925
650.5770
Relative Abundance
m/z

$[PC(32:0)+K]^+$

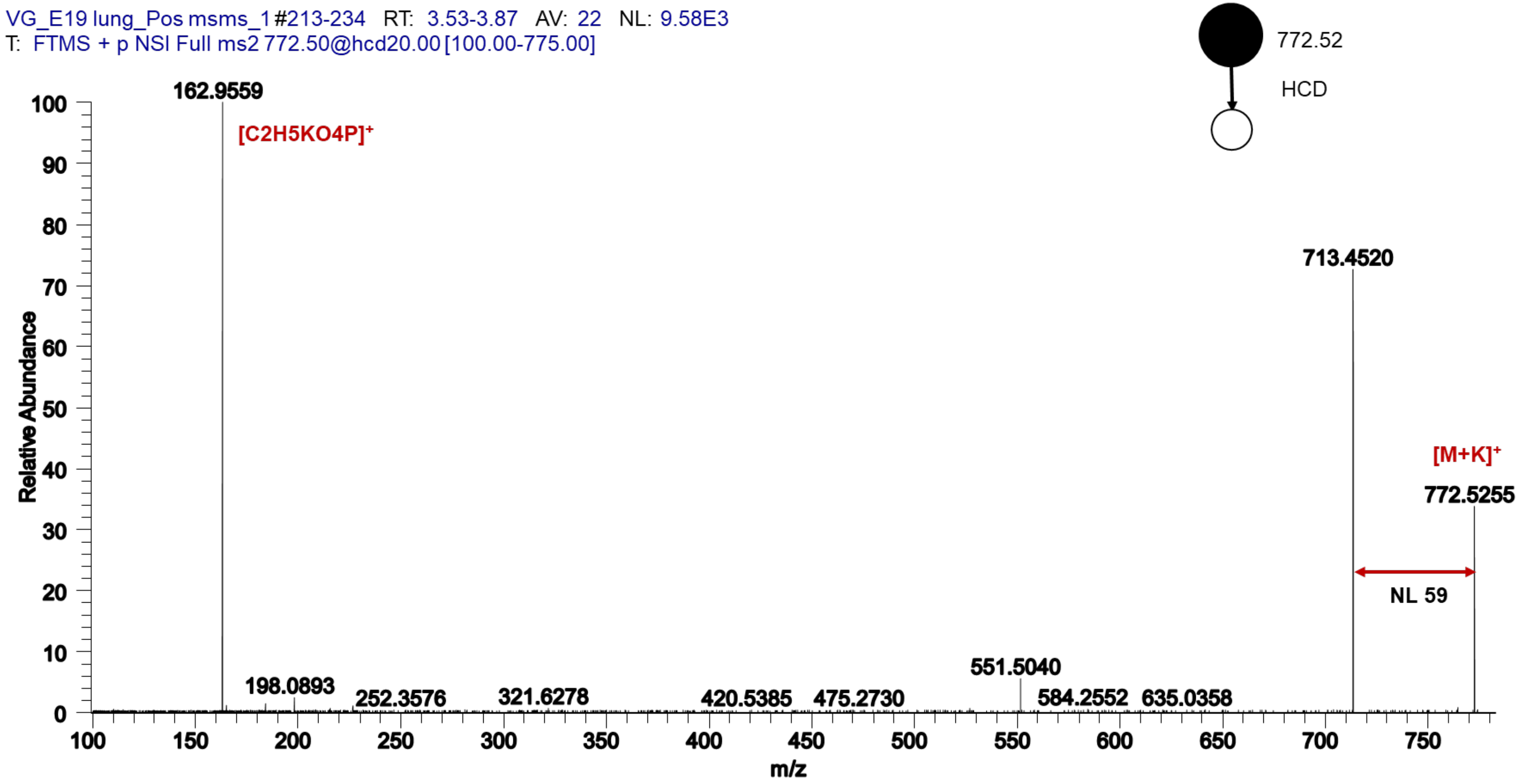

VG_E19 lung_Pos msms_1 #213-234 RT: 3.53-3.87 AV: 22 NL: 9.58E3
T: FTMS + p NSI Full ms2 772.50@hcd20.00 [100.00-775.00]
772.52
HCD
162.9559
[C2H5KO4P]+
713.4520
[M+K]+
772.5255
NL 59
198.0893
252.3576
321.6278
420.5385
475.2730
551.5040
584.2552
635.0358
Relative Abundance
m/z

$[LPC(14:0)+H]^+$

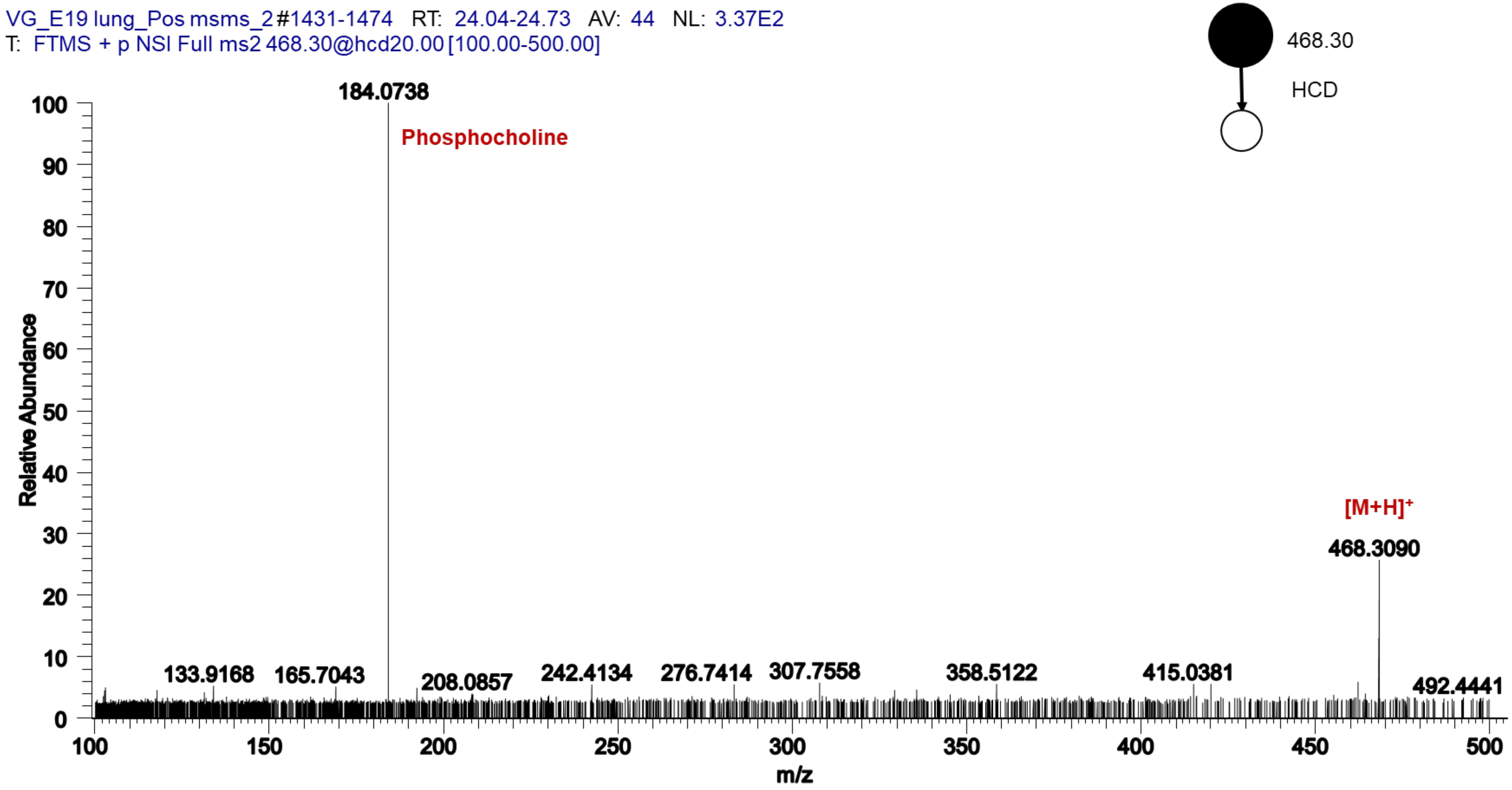

VG_E19 lung_Pos msms_2 #1431-1474 RT: 24.04-24.73 AV: 44 NL: 3.37E2
T: FTMS + p NSI Full ms2 468.30@hcd20.00 [100.00-500.00]
468.30
HCD
184.0738
Phosphocholine
[M+H]+
468.3090
133.9168
165.7043
208.0857
242.4134
276.7414
307.7558
358.5122
415.0381
492.4441
Relative Abundance
m/z

[LPC(18:2)+H]$^+$

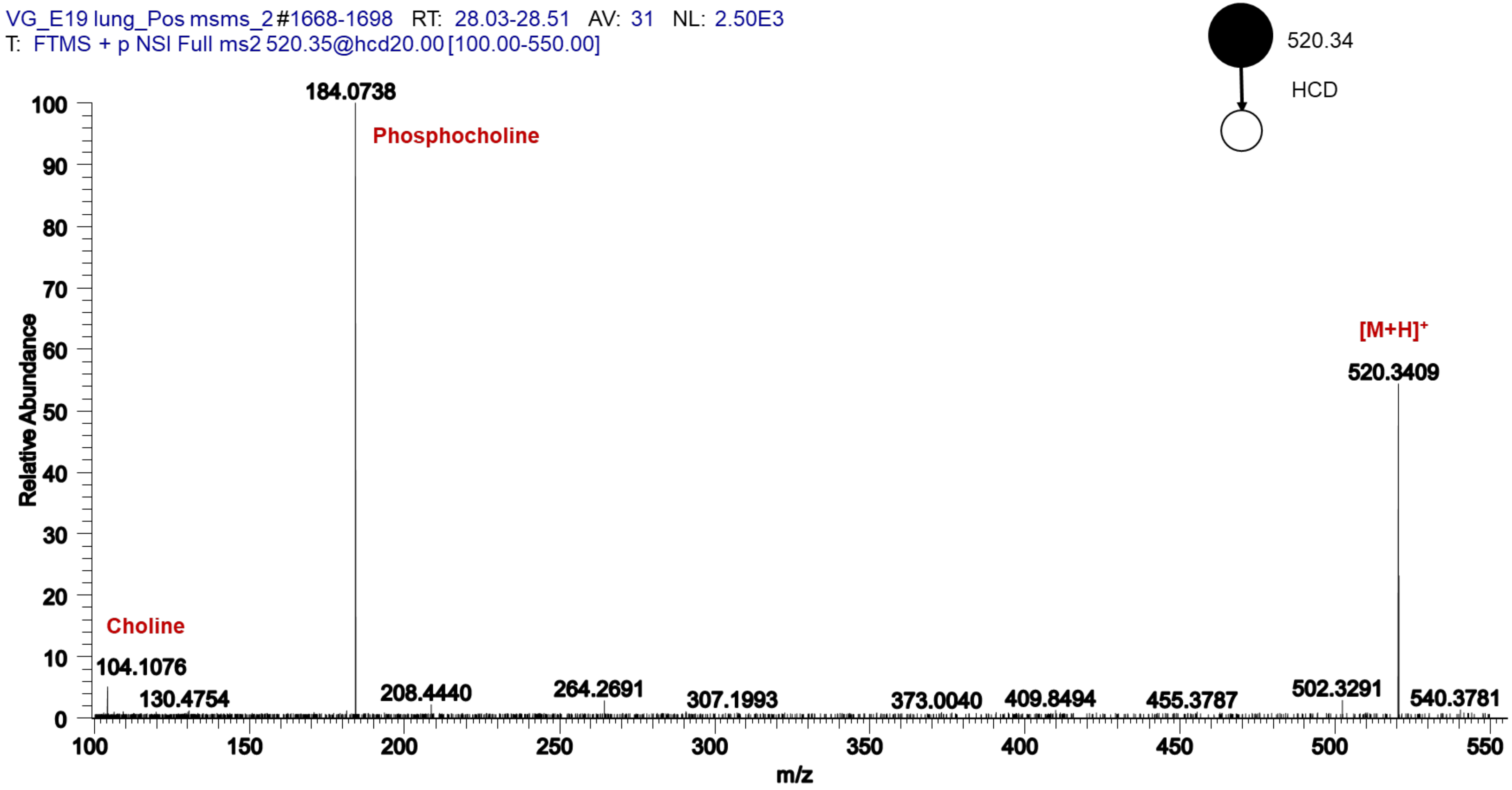
VG_E19 lung_Pos msms_2 #1668-1698 RT: 28.03-28.51 AV: 31 NL: 2.50E3
T: FTMS + p NSI Full ms2 520.35@hcd20.00 [100.00-550.00]
520.34
HCD
184.0738
Phosphocholine
[M+H]+
520.3409
Choline
104.1076
130.4754
208.4440
264.2691
307.1993
373.0040
409.8494
455.3787
502.3291
540.3781
Relative Abundance
m/z
0
10
20
30
40
50
60
70
80
90
100
150
200
250
300
350
400
450
500
550

[LPC(18:2)+Na]$^+$

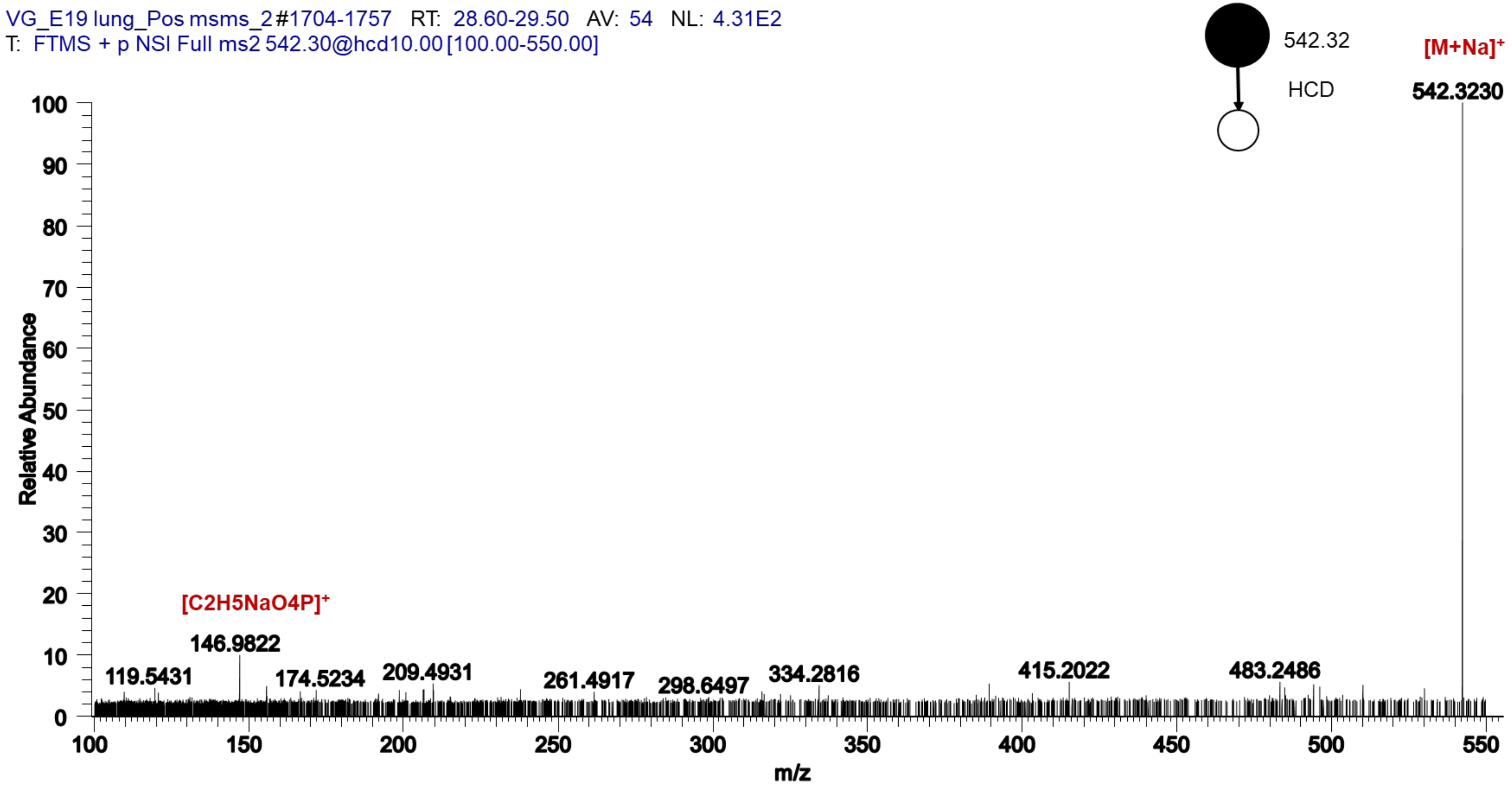
VG_E19 lung_Pos msms_2 #1704-1757 RT: 28.60-29.50 AV: 54 NL: 4.31E2
T: FTMS + p NSI Full ms2 542.30@hcd10.00 [100.00-550.00]
542.32
HCD
[M+Na]+
542.3230
[C2H5NaO4P]+
146.9822
119.5431
174.5234
209.4931
261.4917
298.6497
334.2816
415.2022
483.2486
Relative Abundance
m/z

$[LPC(18:2)+K]^+$

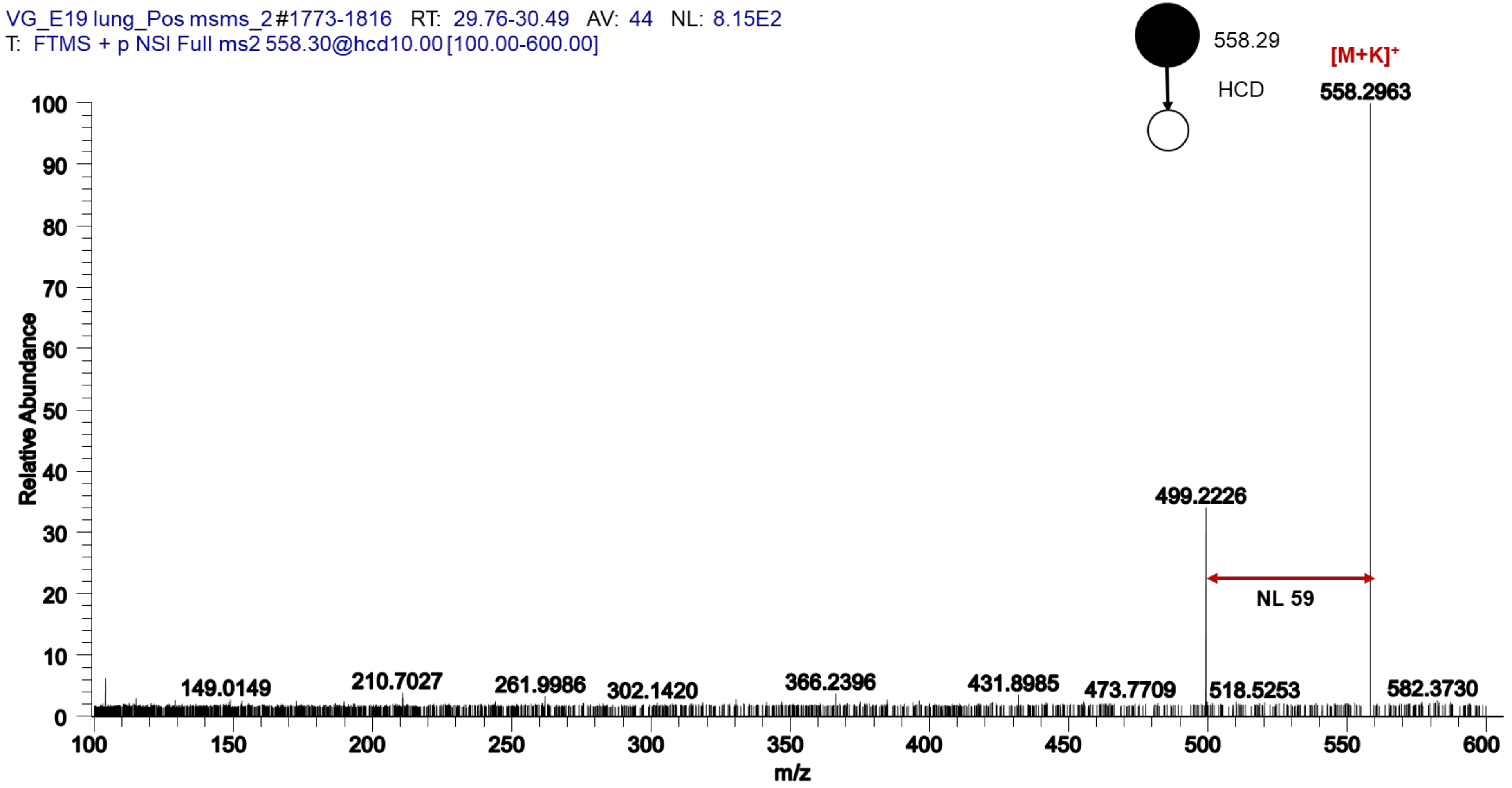

VG_E19 lung_Pos msms_2 #1773-1816 RT: 29.76-30.49 AV: 44 NL: 8.15E2
T: FTMS + p NSI Full ms2 558.30@hcd10.00 [100.00-600.00]
558.29
HCD
[M+K]+
558.2963
499.2226
NL 59
149.0149
210.7027
261.9986
302.1420
366.2396
431.8985
473.7709
518.5253
582.3730
Relative Abundance
m/z

$[LPC(18:1)+H]^+$

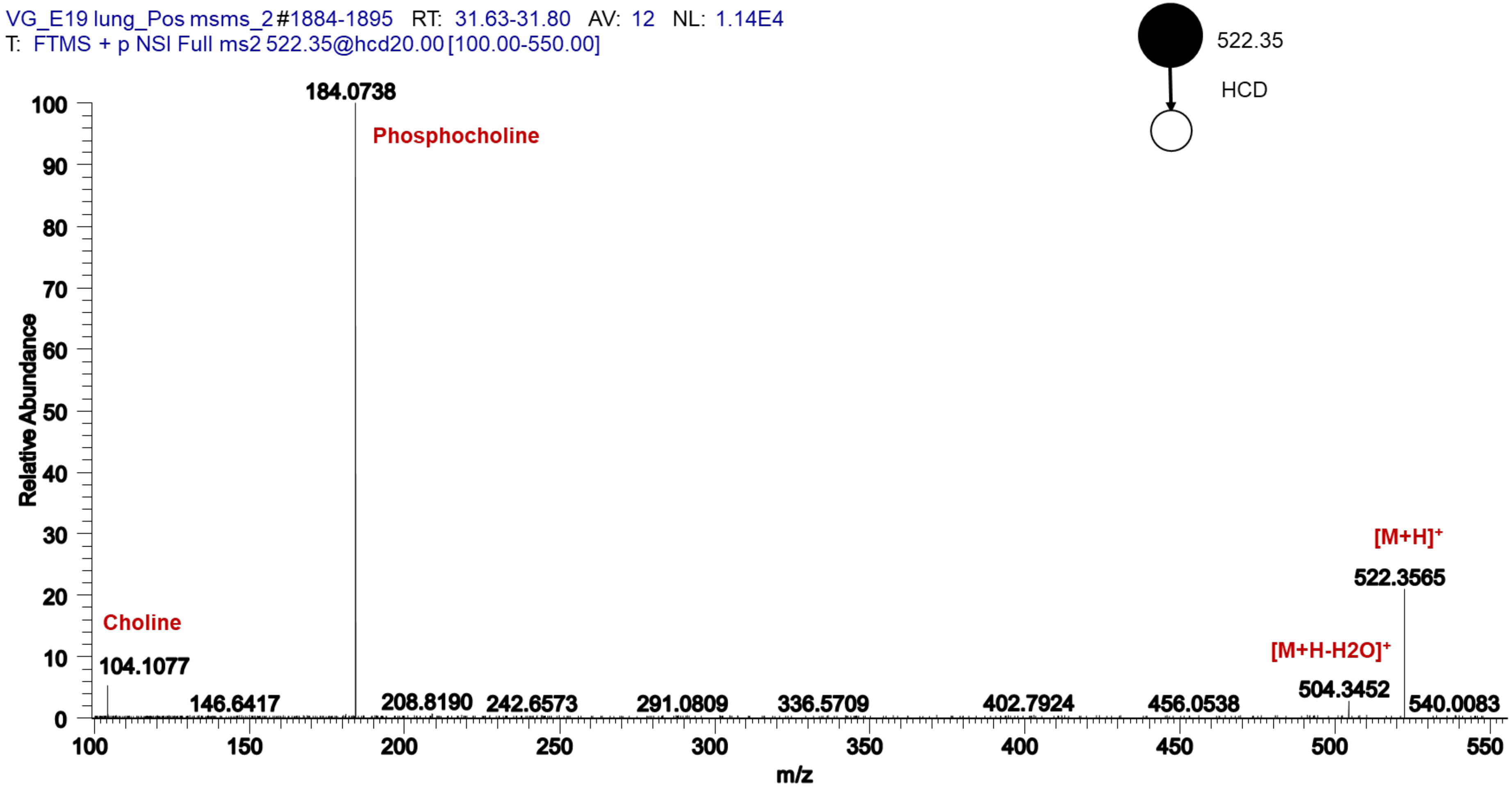

VG_E19 lung_Pos msms_2 #1884-1895 RT: 31.63-31.80 AV: 12 NL: 1.14E4
T: FTMS + p NSI Full ms2 522.35@hcd20.00 [100.00-550.00]
522.35
HCD
184.0738
Phosphocholine
Choline
104.1077
146.6417
208.8190
242.6573
291.0809
336.5709
402.7924
456.0538
[M+H-H2O]+
504.3452
[M+H]+
522.3565
540.0083
Relative Abundance
m/z

[LPC(18:1)+K]+

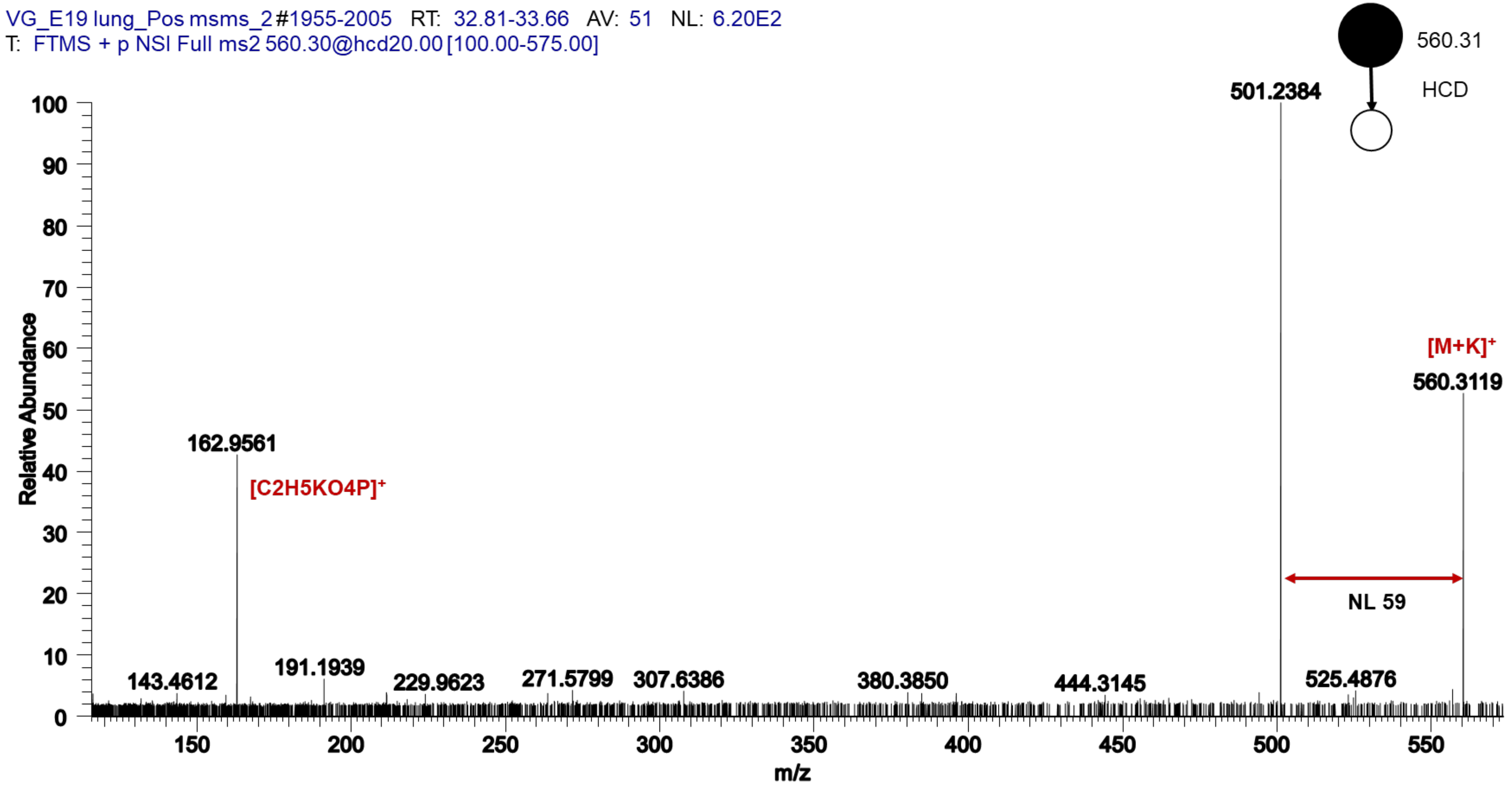
VG_E19 lung_Pos msms_2#1955-2005 RT: 32.81-33.66 AV: 51 NL: 6.20E2
T: FTMS + p NSI Full ms2 560.30@hcd20.00 [100.00-575.00]
560.31
HCD
501.2384
[M+K]+
560.3119
162.9561
[C2H5KO4P]+
NL 59
143.4612
191.1939
229.9623
271.5799
307.6386
380.3850
444.3145
525.4876
Relative Abundance
m/z

[LPC(18:1)+Na]⁺ / [LPC(20:4)+H]⁺

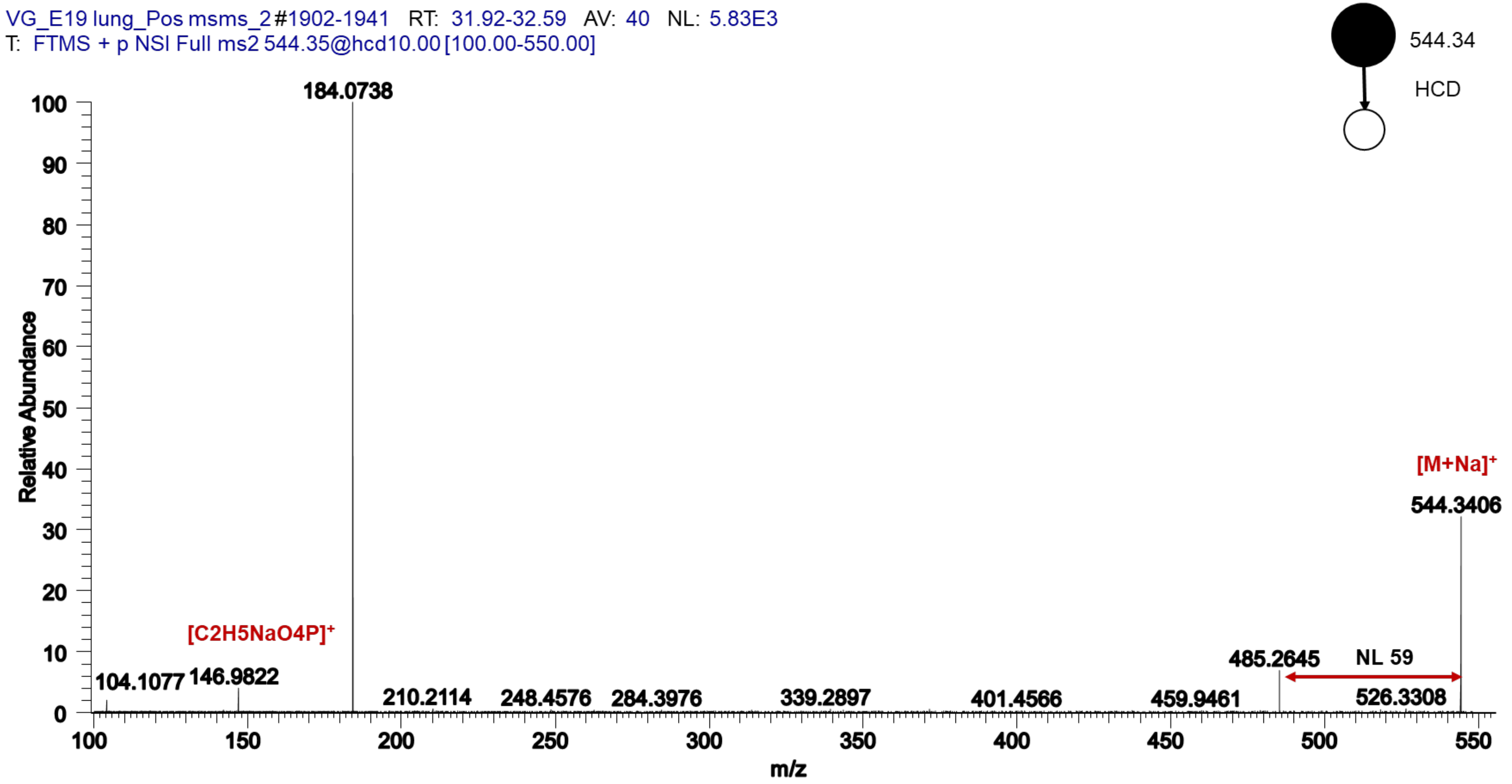

[LPC(20:4)+H]$^+$ / [LPC(18:1)+Na]$^+$

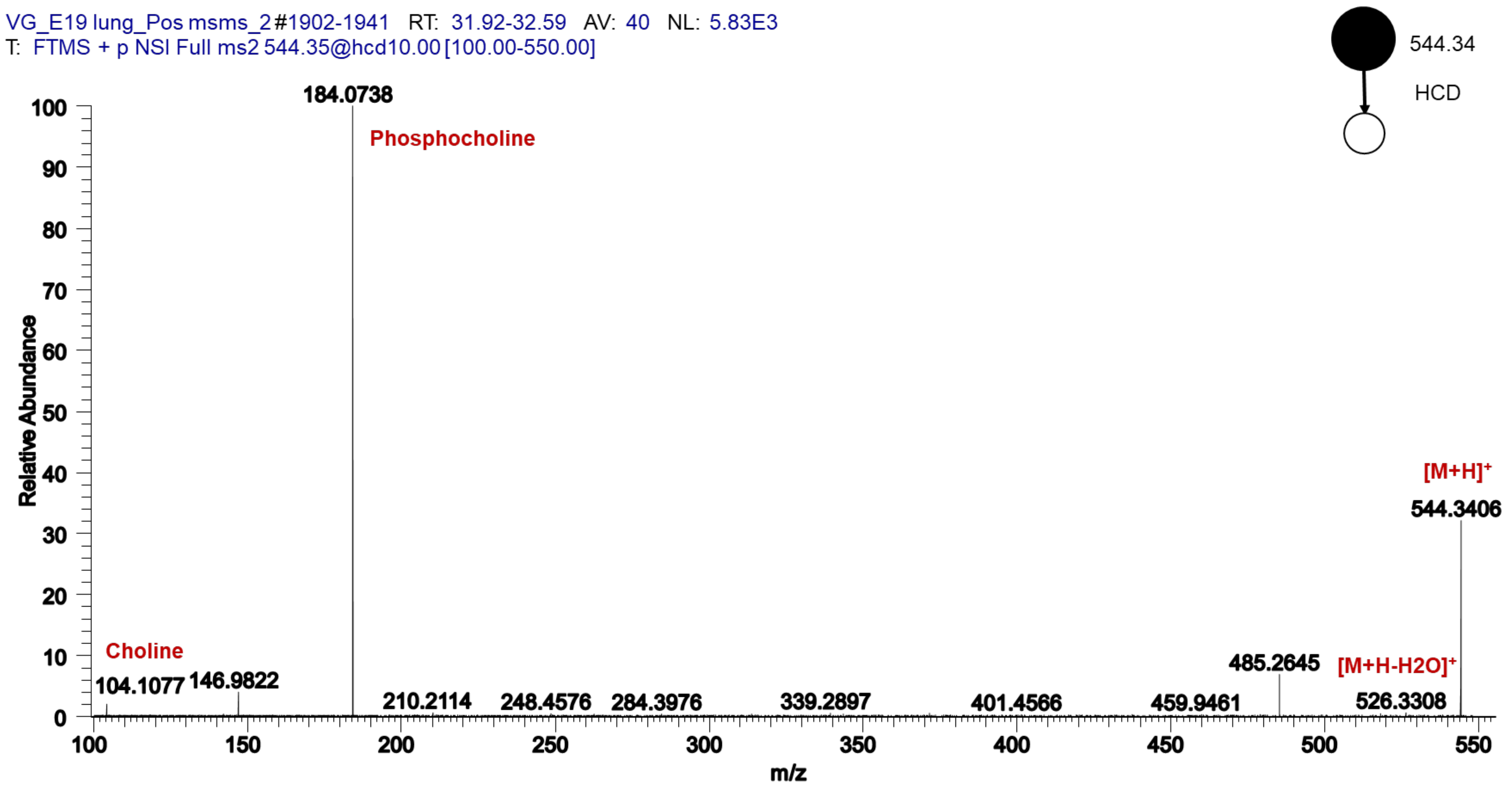

$[PC(O\text{-}34{:}1)+H]^+$ / $[PC(P\text{-}34{:}0)+H]^+$

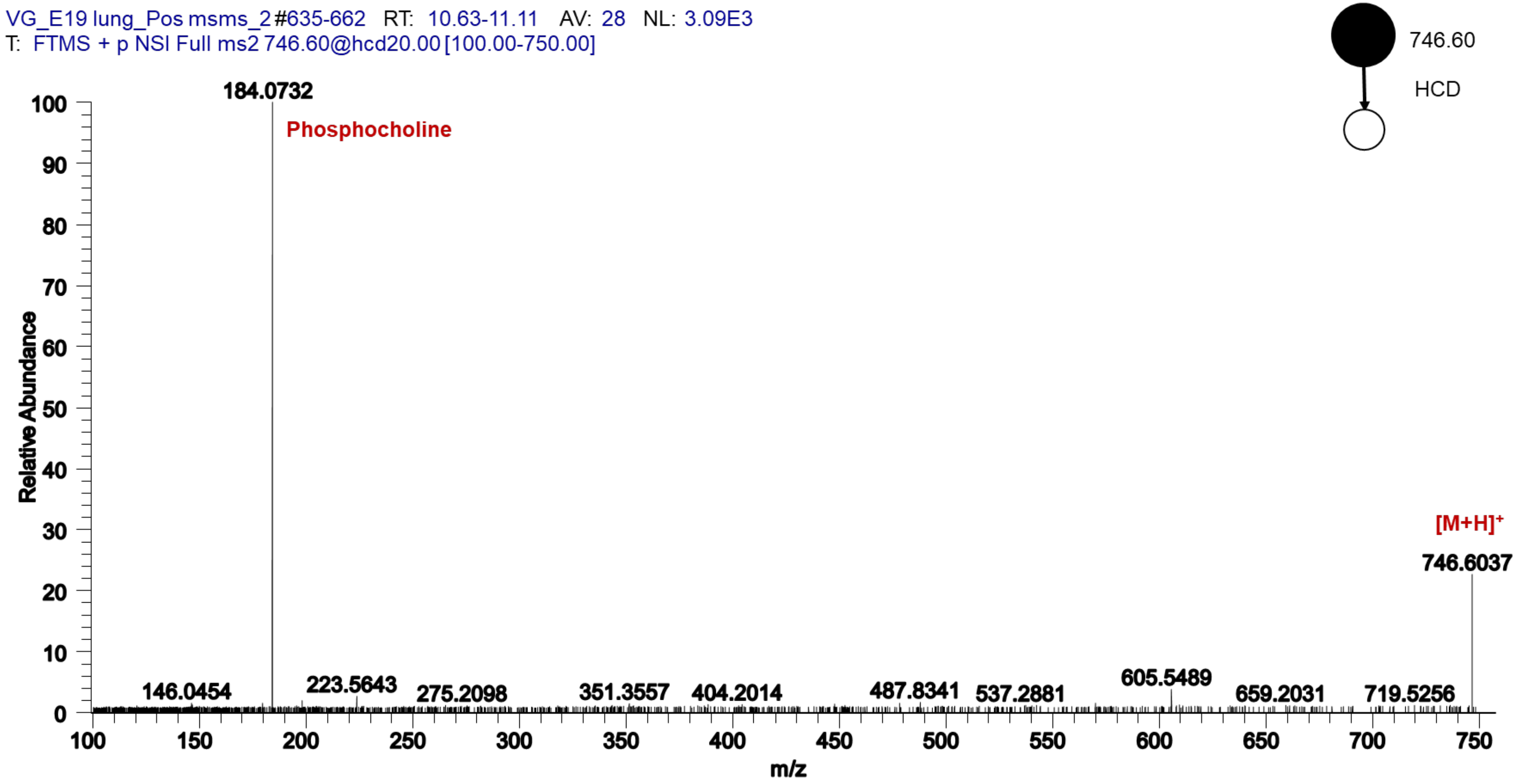

[SM(34:1)+H]+

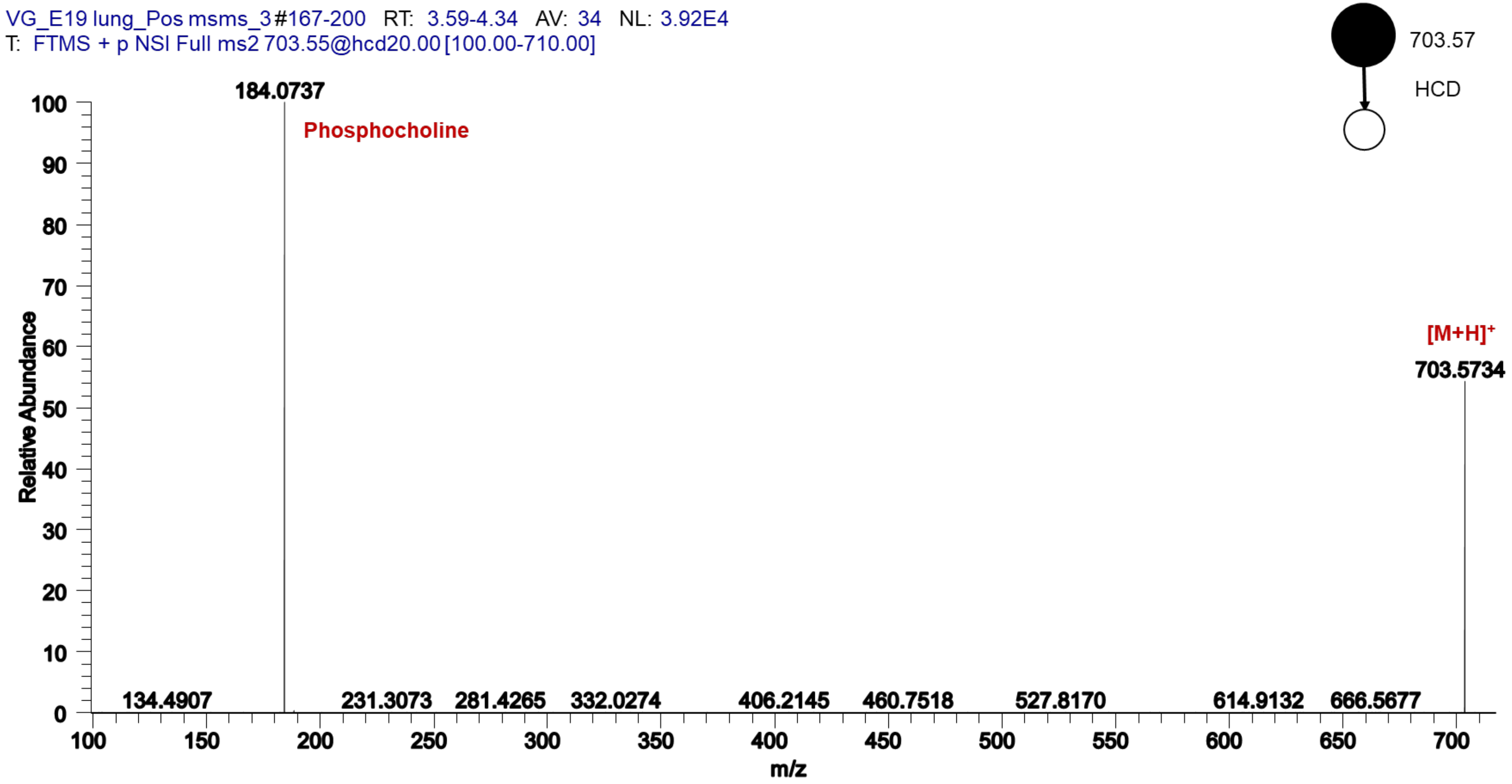
VG_E19 lung_Pos msms_3#167-200 RT: 3.59-4.34 AV: 34 NL: 3.92E4
T: FTMS + p NSI Full ms2 703.55@hcd20.00 [100.00-710.00]
703.57
HCD
184.0737
Phosphocholine
[M+H]+
703.5734
134.4907
231.3073
281.4265
332.0274
406.2145
460.7518
527.8170
614.9132
666.5677
Relative Abundance
m/z

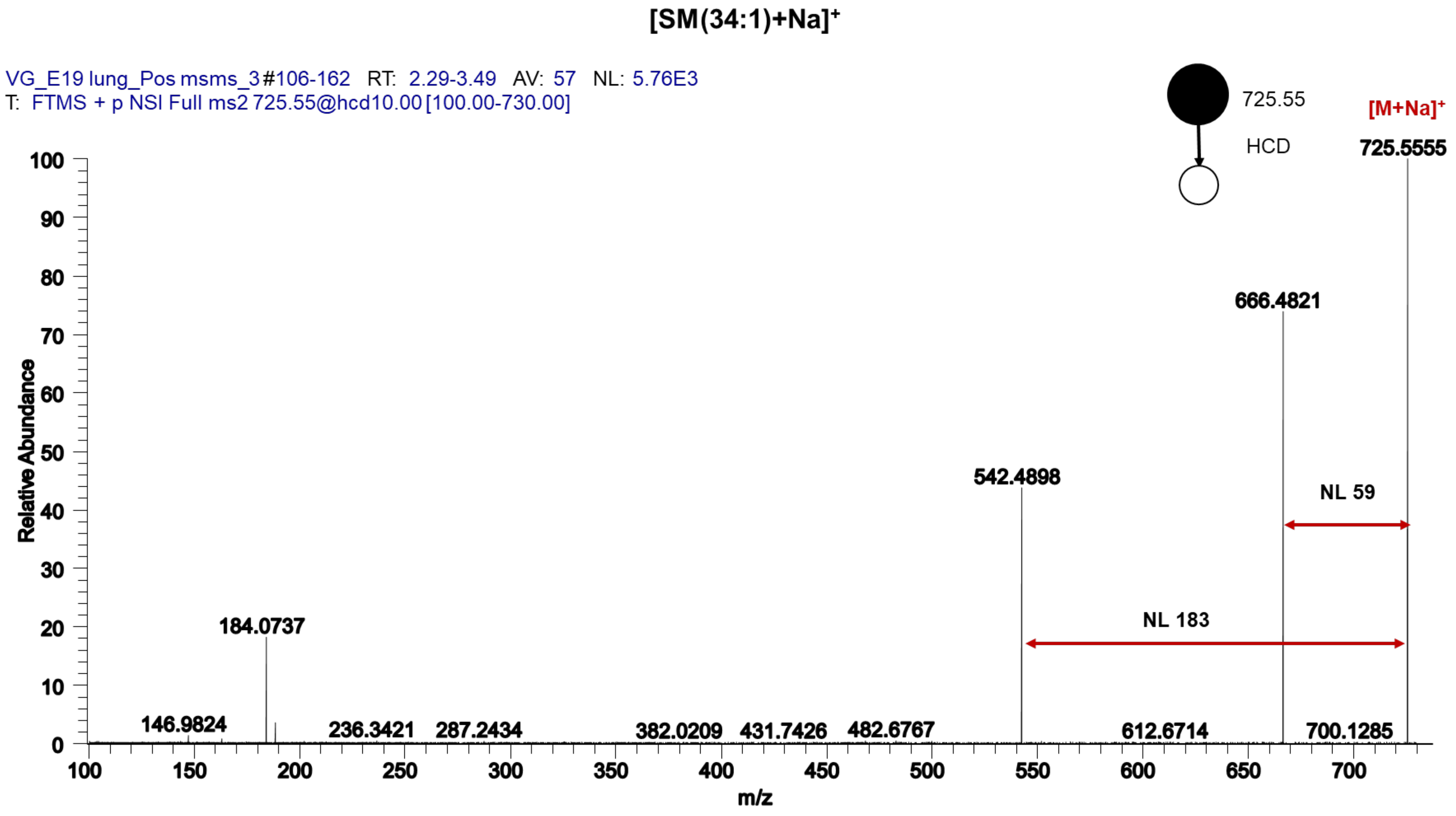
[SM(34:1)+Na]+
VG_E19 lung_Pos msms_3#106-162 RT: 2.29-3.49 AV: 57 NL: 5.76E3
T: FTMS + p NSI Full ms2 725.55@hcd10.00 [100.00-730.00]
725.55
HCD
[M+Na]+
725.5555
666.4821
542.4898
NL 59
NL 183
184.0737
146.9824
236.3421
287.2434
382.0209
431.7426
482.6767
612.6714
700.1285
Relative Abundance
m/z

[SM(34:1)+K]$^{+}$

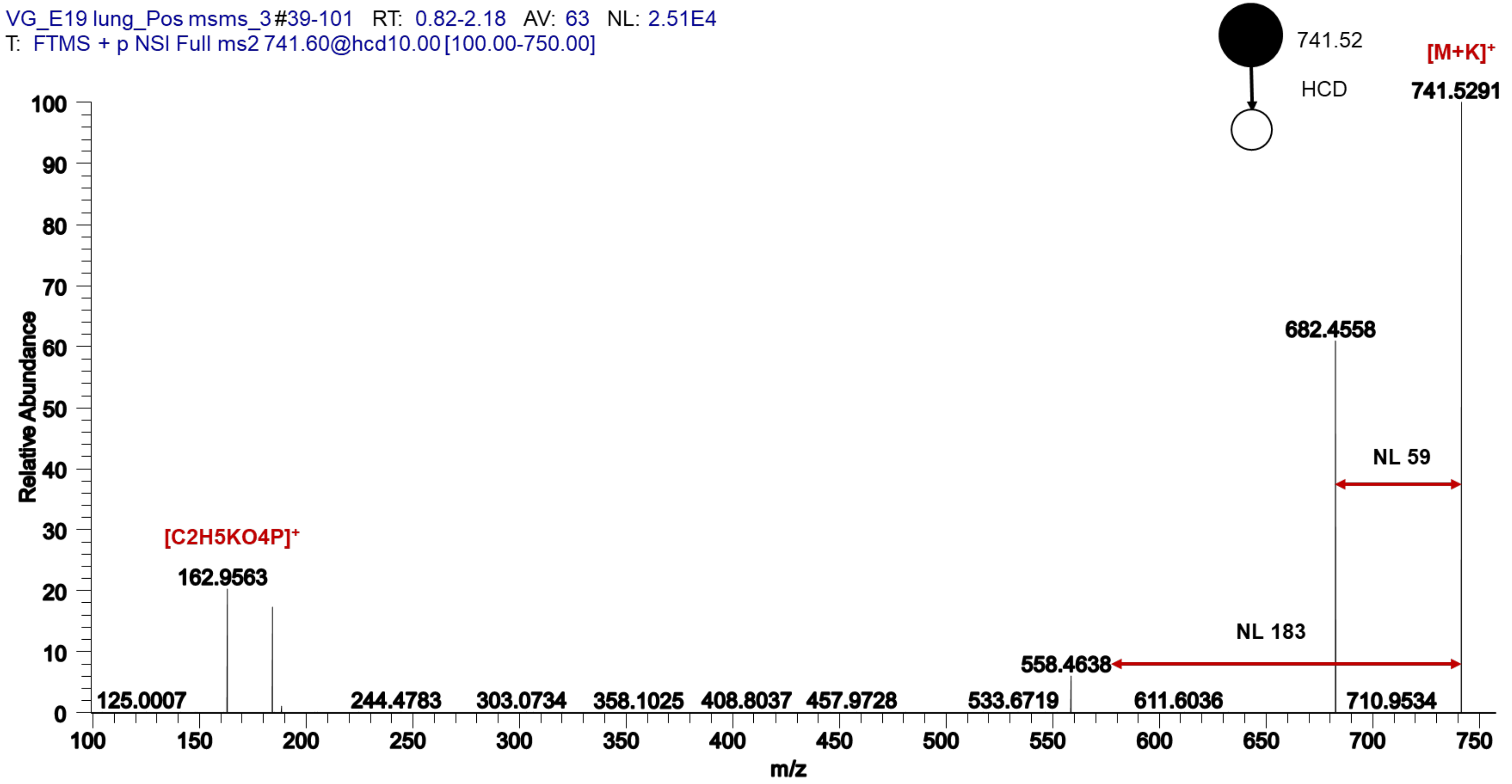

VG_E19 lung_Pos msms_3 #39-101 RT: 0.82-2.18 AV: 63 NL: 2.51E4
T: FTMS + p NSI Full ms2 741.60@hcd10.00 [100.00-750.00]
741.52
HCD
[M+K]+
741.5291
682.4558
NL 59
[C2H5KO4P]+
162.9563
NL 183
558.4638
125.0007
244.4783
303.0734
358.1025
408.8037
457.9728
533.6719
611.6036
710.9534
Relative Abundance
m/z

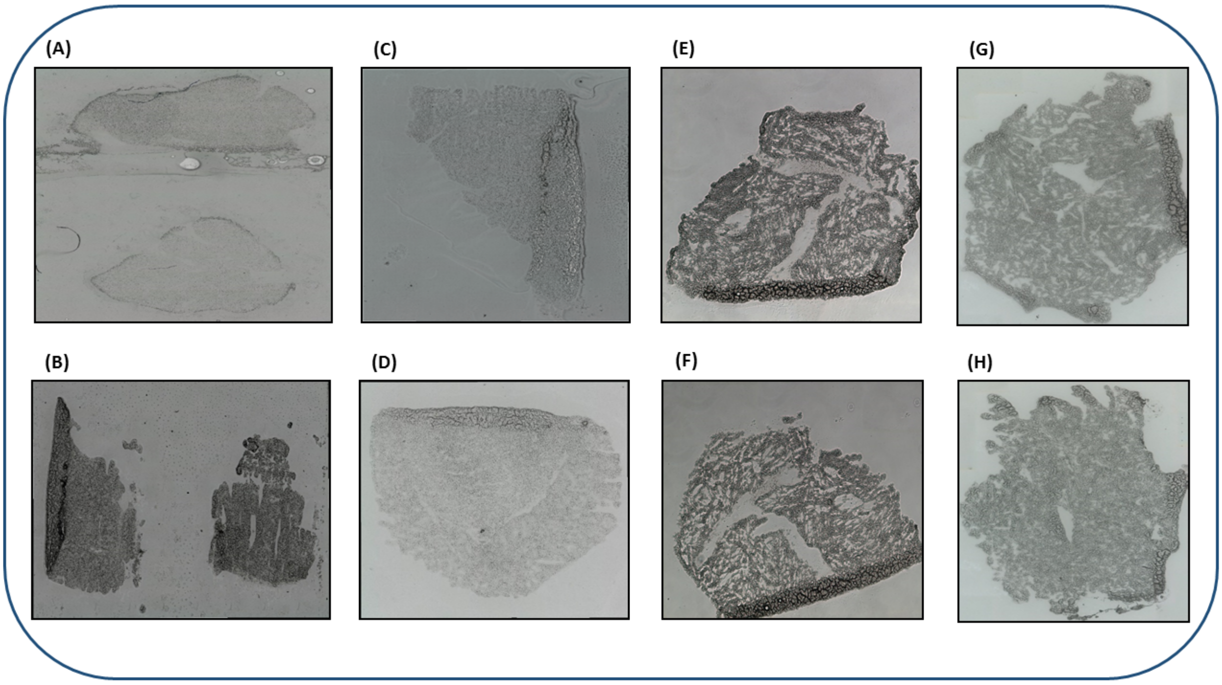

Fig. S1: Optimization of tissue sectioning procedure to obtain sections from fresh snap-frozen wild type (WT) and *Pex11β* knockout (KO) late fetal E19 mouse lungs for mass spectrometry imaging (MSI) experiments. A) Microscopic phase contrast image of paraformaldehyde (PFA)-fixed, optimal cutting temperature (OCT)-embedded WT and KO E19 lung tissue sections mounted on same glass slide. B) Microscopic image of WT and KO E19 lung tissue sections mounted on same glass slide without using any embedding material, problem of different section quality; WT section contains a fold on the left; the KO section on the right side is broken several times in longitudinal direction. C & D) Microscopic images of unequal thickness and folds in KO and WT E19 lung tissue sections mounted on different glass slides. E & F) Microscopic images of the unequal thickness of WT E19 lung tissue sections. G & H) Finally selected microscopic images of equal thickness (≈12 μm) of WT and KO E19 lung tissue sections mounted on different glass slides using deionized water

(ice) as an adhesive. The small artefacts due to compression only at the rim can be omitted by choosing the region of interest in the measurement. The tissue sections, i) with no scratches and no broken parts, ii) no tissue foldings, iii) flat (same focal plane) and uniform thickness throughout the section, iv) no air bubbles and v) no other possible mechanical artefacts were considered as a good quality of sections and used for further MALDI MSI experiments.

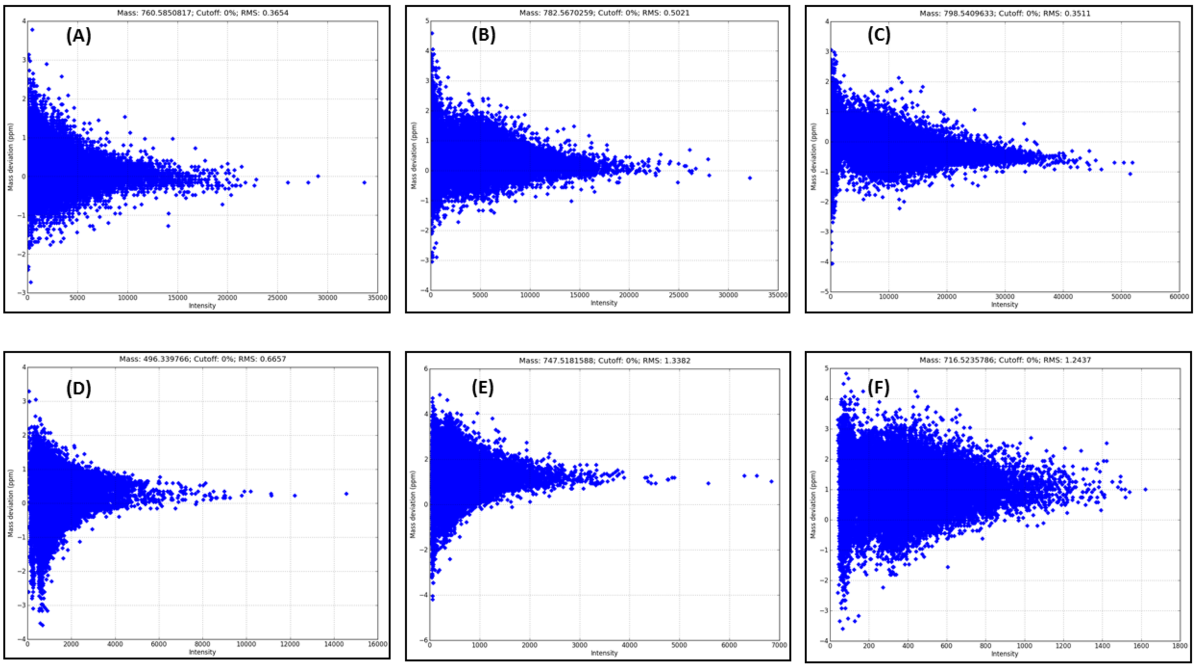

Fig. S2: High mass accuracy mass spectrometry imaging (MSI) data acquisition in positive- (A-D) and negative-ion mode (E-F). Mass deviation of lipid species as a function of intensities, root-mean-square error values (RMSE) of respective lipid species were represented in parts per million (RMSE ≤ 2ppm).

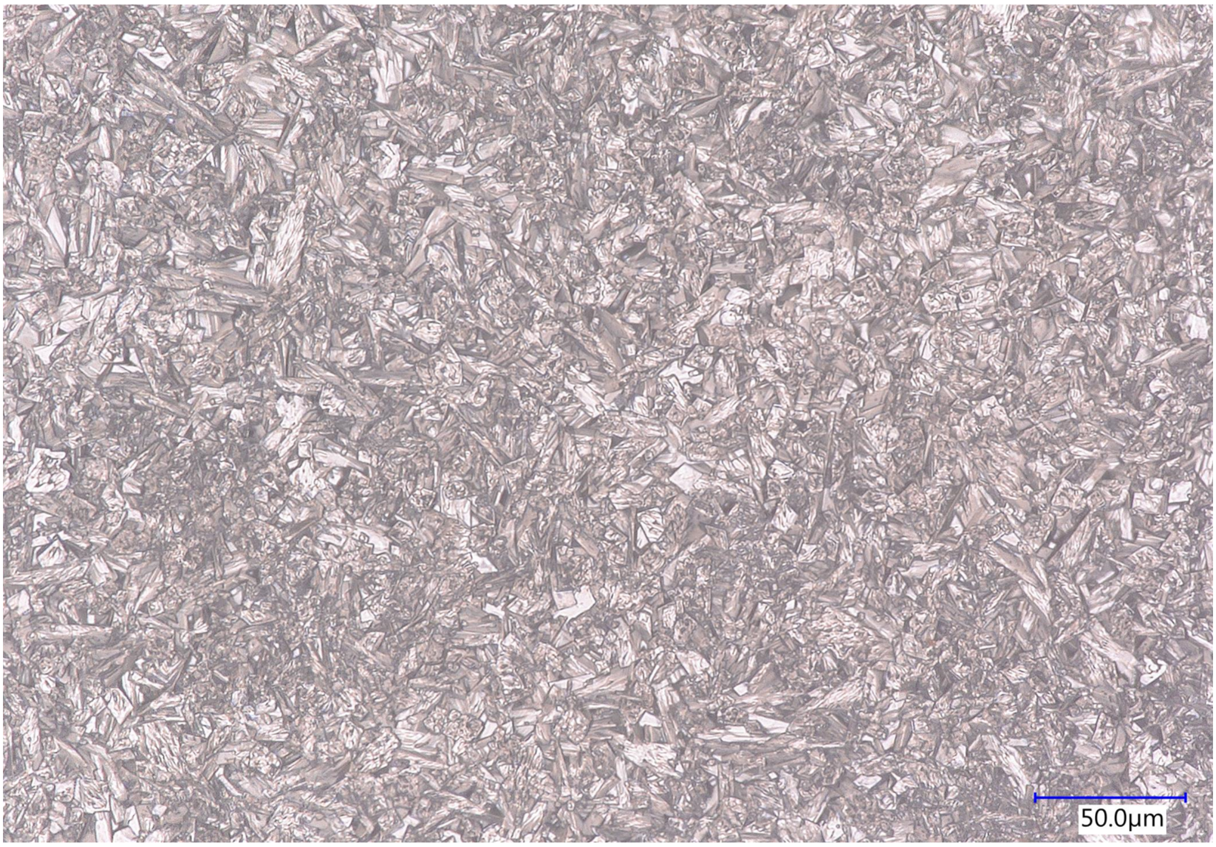
50.0μm

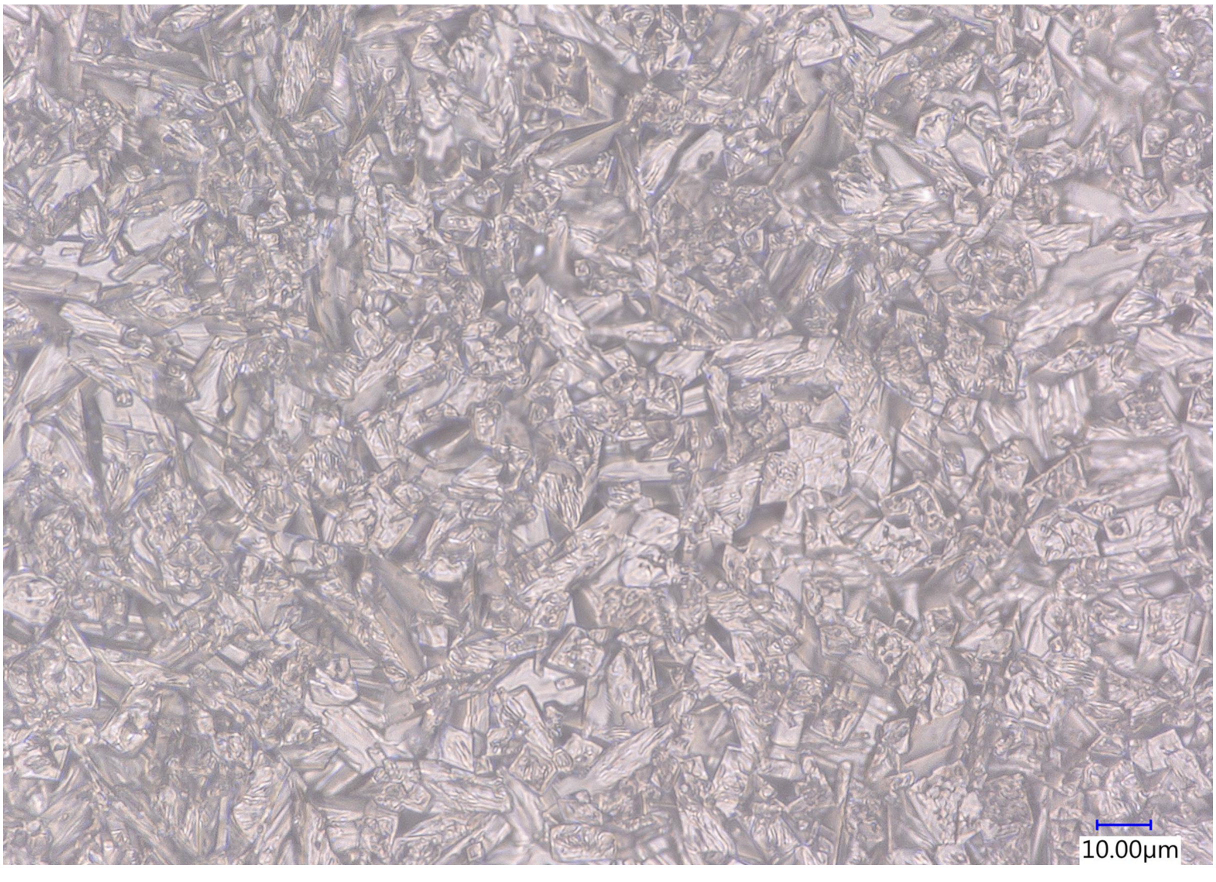
10.00μm

Figure S3: Microscopic optical images of 2,5-Dihydoxybenzoic acid (DHB) matrix crystals deposited on tissue surfaces using "SMALDIPrep" automatic pneumatic ultrafine matrix sprayer (TransMIT GmbH, Giessen, Germany). Optical images were obtained using a Keyence VHX-5000 digital microscope (Keyence Germany GmbH).

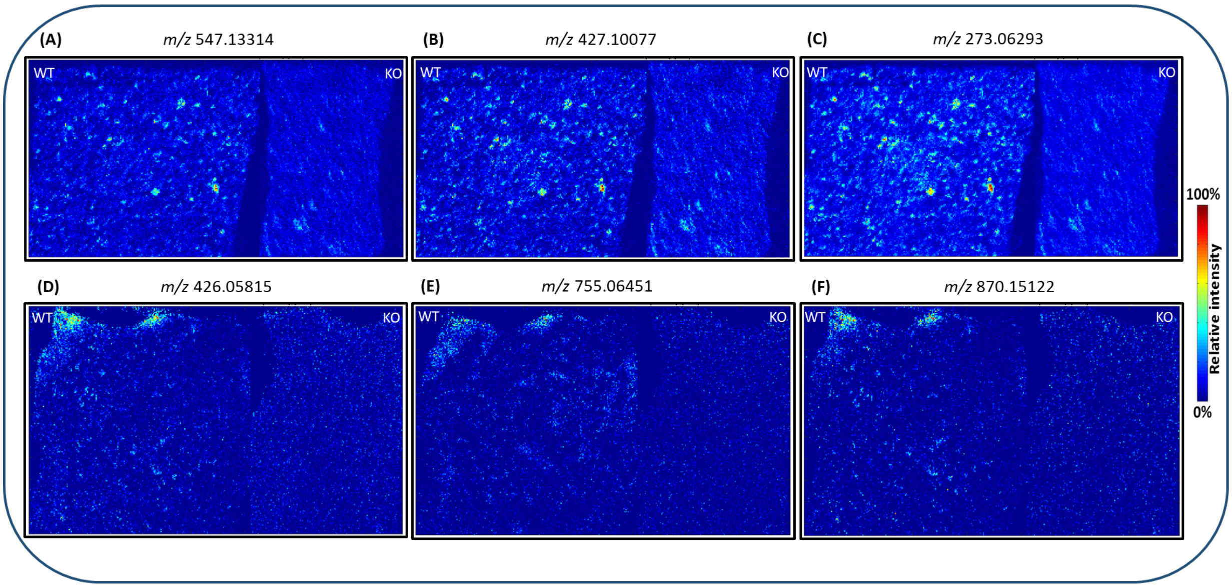

Fig. S4: Distribution of matrix cluster signals in replicate measurement samples of wild type (WT) and *Pex11β* knockout (KO) late fetal E19 mouse lung tissue sections. (A-C) Evenly distributed 4-Nitroaniline (pNA) matrix cluster images in negative-ion mode. (D-F) Evenly distributed 2,5-Dihydroxybenzoic acid (DHB) matrix cluster images in positive-ion mode.

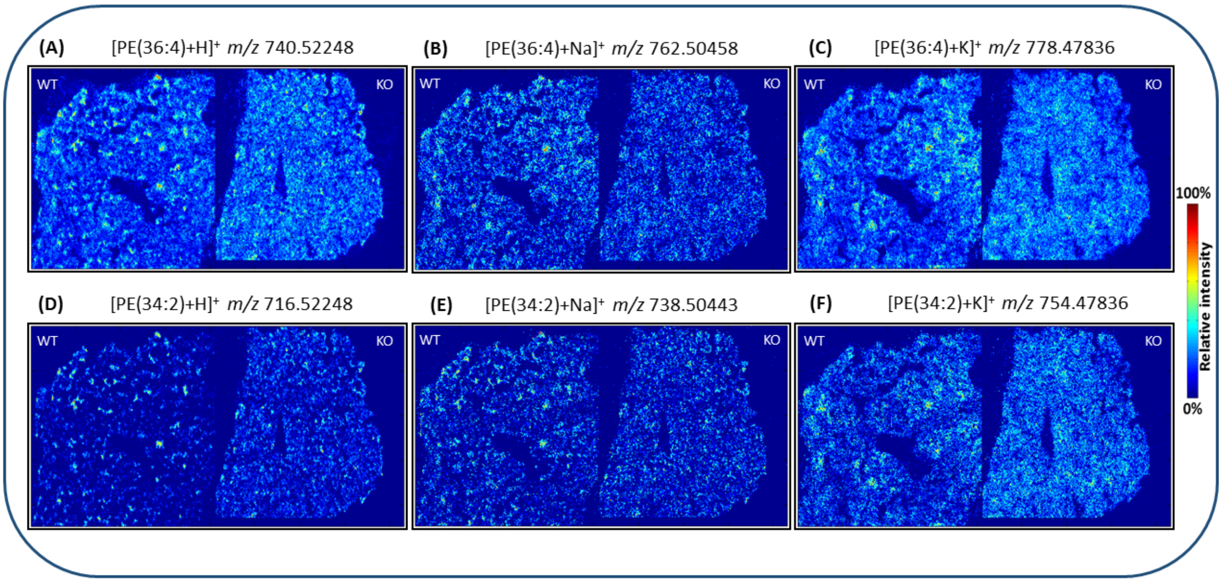

Fig. S5: Homogenous distribution of endogenous molecules with different charge carriers (H^+, Na^+, K^+) in wild type (WT) and *Pex11β* knockout (KO) late fetal E19 mouse lung tissue sections, measured by atmospheric-pressure MALDI mass spectrometry imaging (MSI) experiments. (A-C) Uniform distribution of PE(36:4) with different charge carriers, measured in positive-ion mode. (A) $[PE(36:4)+H]^+$, *m/z* 740.52248, (B) $[PE(36:4)+Na]^+$, *m/z* 762.50458 and (C) $[PE(36:4)+K]^+$,*m/z* 778.47836. (D-F) Uniform distribution of PE(34:2) with different charge carriers, measured in positive-ion mode. (D) $[PE(34:2)+H]^+$, *m/z* 716.52248 (E), $[PE(34:2)+Na]^+$, *m/z* 738.50443 and (F) $[PE(34:2)+K]^+$, *m/z* 754.47836.

Supplementary Table 4: Comparison of mean abundance values and ratios of lipid species between wild type (WT) and *Pex11β* knockout (KO) mice lung tissue sections using different normalization approaches in negative- and positive-ion mode.

**Sample type**	**Mean abundance values and ratios**		
	Without normalization	TIC normalization	Reference peak (matrix) normalization
Negative-ion mode, $[PG(34:1)-H]^-$, *m/z* 747.51815			
Wild type	526.23647	0.00156	1.07913
Knockout	279.68205	0.00036	0.67696
KO/WT	0.53	0.23	0.63
Positive-ion mode, $[SHexCer(t33:1)+K]^+$, *m/z* 820.46415			
Wild type	93.51792	0.00014	0.36839
Knockout	261.84857	0.00061	1.02317
KO/WT	2.80	4.36	2.78

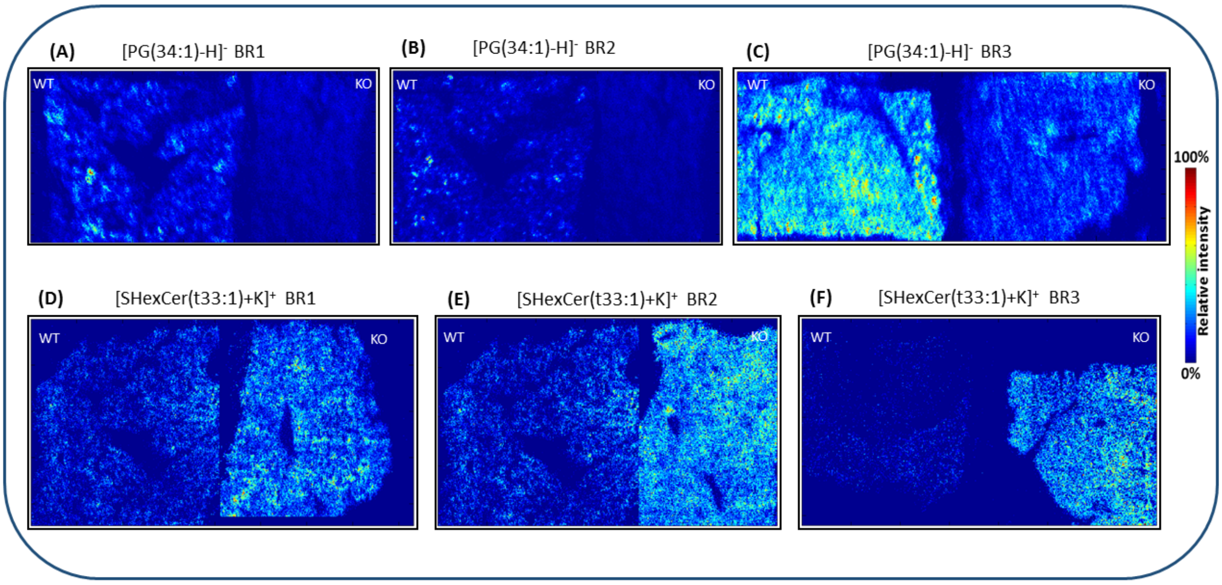

Fig. S6: Confirmation of differential abundance of lipid species in wild type (WT) and *Pex11β* knockout (KO) late fetal E19 mouse lung tissue sections from biological replicates (BR). (A-C) $[PG(34{:}1)\text{-}H]^-$, *m/z* 747.51815, (D-F) $[SHexCer\ (t33{:}1)+K]^+$, *m/z* 820.46415 differential expression, confirmed for biological replicates in negative- and positive-ion mode.
